# Drug repurposing for COVID-19 using machine learning and mechanistic models of signal transduction circuits related to SARS-CoV-2 infection

**DOI:** 10.1038/s41392-020-00417-y

**Published:** 2020-12-11

**Authors:** Carlos Loucera, Marina Esteban-Medina, Kinza Rian, Matías M. Falco, Joaquín Dopazo, María Peña-Chilet

**Affiliations:** 1 Clinical Bioinformatics Area. Fundación Progreso y Salud (FPS). CDCA, Hospital Virgen del Rocio, 41013 Sevilla, Spain; 2 Computational Systems Medicine, Institute of Biomedicine of Seville (IBIS), Hospital Virgen del Rocio, 41013 Sevilla, Spain; 3 Bioinformatics in Rare Diseases (BiER). Centro de Investigación Biomédica en Red de Enfermedades Raras (CIBERER), FPS, Hospital Virgen del Rocío, 41013 Sevilla, Spain; 4 FPS/ELIXIR-es, Hospital Virgen del Rocío, Sevilla, 42013 Spain

**Keywords:** Target identification, Predictive medicine, Infectious diseases

**Dear Editor**,

Drug repurposing is a convenient alternative when the need for new drugs in an unexpected medical scenario is urgent, as is the case of emerging pathogens. In recent years, approaches based on network biology have demonstrated to be superior to gene-centric ones.^1^ Here, we use an innovative methodology that combines mechanistic modeling of the signal transduction circuits related to SARS-CoV-2 infection (the COVID-19 disease map) with a machine-learning algorithm that learns potential causal interactions between proteins, already targets of drugs, and specific signaling circuits in the COVID-19 disease map, to suggest potentially repurposable drugs.

Mechanistic models of pathways provide a natural bridge from variations at the scale of gene activity (transcription) to variations in phenotype (at the level of cells, tissues, or organisms). Actually, mechanistic models of human signaling pathways have been successfully used to uncover specific molecular mechanisms behind different diseases, to reveal modes of action of drugs, and to suggest personalized treatments. However, the most interesting property of mechanistic models is that they can be used to predict the consequences of interventions as, for example, the effect of targeted drugs.^2^ The availability of a COVID-19 disease map^3^ can be used to build a realistic mechanistic model of the SARS-CoV-2 infection and all the downstream functional consequences that occur in the host cells. This disease map is a set of signaling transduction circuits that contain human proteins that interact with viral proteins and their upstream and downstream connections (see Supplementary Results and Supplementary Table S1, with the 277 resulting circuits from a total of 49 KEGG pathways). These affected circuits ultimately trigger cell functionalities, whose perturbation by the virus causes the COVID-19 symptoms or disease hallmarks. The main hallmarks used here to properly fit in the Uniprot annotations that define the circuit functionalities were: (1) host–virus interaction, (2) inflammatory response, (3) immune activity, (4) antiviral defense, (5) endocytosis, (6) replication, and (7) energetics. This disease map will be dynamically updated as new biological knowledge is generated by the Disease Maps community.^3^

Interestingly, the notion of causality provided by the mechanistic model of the COVID-19 disease map can be exploited beyond the own pathways modeled. Actually, machine-learning methods can be used to extrapolate the effect that other proteins, even if these are not part of the disease map modeled, can have over the signaling circuits of the map. We have recently demonstrated that machine learning can be used over a large dataset of gene expression data to learn how the disease hallmarks of Fanconi Anemia, a rare condition caused by defective DNA repair in the cells, could be predicted even from proteins apparently unconnected to its disease map. Such an approach, used for drug repurposing, produced a list of potentially repurposable drugs, some of which (e.g., Gefitinib and Afatinib) were further validated.^4^

Here, we assume that other proteins which, according to the signaling circuit activity estimations of our mechanistic model, have an influence on the status of these hallmarks might be playing some type of upstream regulator role. Consequently, this potential modulator capacity could make them suitable candidates to become therapeutic targets. Since we are interested in drug repurposing, we will only consider as candidates therapeutic targets from drugs that are already approved for other indications. To achieve so, a total of 2045 human proteins, that are known drug targets (KDTs) of a total of 1735 drugs were extracted from DrugBank. Then, a machine-learning procedure is used to “learn”, using a Multi-task Learning model (specifically a Multi-Output Random Forest regressor combined with SHapley Additive exPlanations to determine the influences of KDTs on specific signaling circuits, see Supplementary Methods for details), the relationships between the KDTs and the activities of the COVID-19 circuits that conform the disease map, as estimated by the mechanistic model, as sketched in Fig. 1a. Over 11,000 gene expression experiments representing different organs and conditions taken from the GTEx repository were used for the learning procedure (see Supplementary Methods). The drugs that target proteins with a highly relevant influence over the COVID-19 modeled hallmarks will be the candidates for repurposing.

**Fig. 1:**
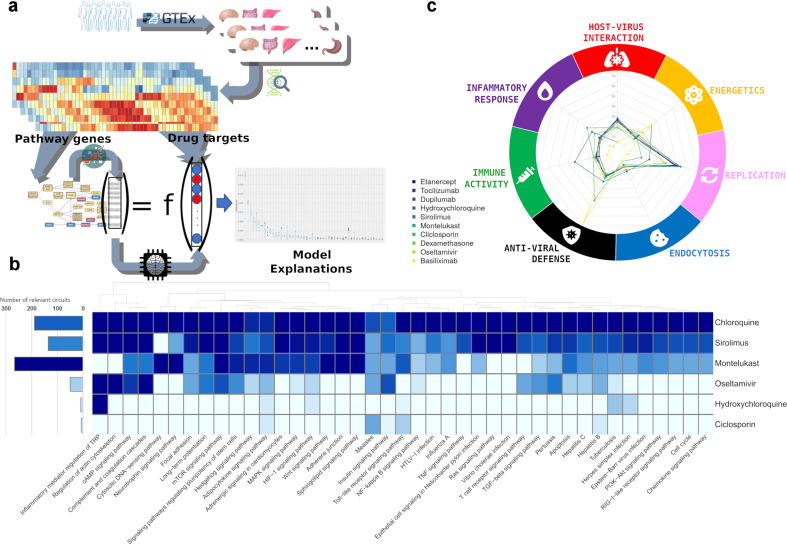
Summary of the drug repurposing strategy and the results obtained. **a** Schema of the procedure followed for finding drug targets that affect the COVID-19 disease map. **b** Two different patterns of circuits affected by six drugs (see text). **c** Radar plot representing the impact of the most relevant drugs listed in Supplementary Table S3 over the different COVID-19 hallmarks, quantified as the number of circuits of the corresponding hallmark are potentially affected

The results showed that 380 out of 2045 original KDTs, targeted by 679 different drugs, out of 1735 tested, have a direct relevant influence on at least one signaling circuit of the COVID-19 disease map (Supplementary Table S2 and Supplementary Fig. S1). Enrichment analysis renders significant Gene Ontology (GO) biological processes related to immune activity, especially to T cell, but also to inflammatory response, and other infectious processes, such as hepatitis, HIV, and papillomavirus infections. Moreover, GO functionalities corresponding to all COVID-19 hallmarks are represented as well. Interestingly, the enrichment in virus-caused perturbations in GEO expression showed enrichment in datasets infected with SARS-CoV and other respiratory-related infections (see Supplementary Fig. S2).

Among the drugs predicted to have a relevant effect, some of them are currently under clinical trials. Interestingly, these drugs define different functional profile templates, which may be useful to speculate similar consequences for other drugs with similar patterns of influence over the signaling circuits that define the disease hallmarks. Thus, some drugs, as Sirolimus, have a strong impact over most of the circuits, while others as Ciclosporin affect only a small number of them (seven circuits) as depicted in Fig. 1b. Indeed, Ciclosporin shares with the well-known hydroxychloroquine circuits within Toll-like and adipocytokine signaling pathways. However, while Ciclosporin is relevant for Insulin signaling, hydroxychloroquine influences Inflammatory mediator regulation of TRP channels and HIF-1 signaling pathways. Chloroquine and ciclosporin are representative of two different modes of action by either affecting massively to almost all COVID-19 disease map circuits or only affecting a few specific ones, respectively. In fact, a recently published compilation of drugs currently in clinical trials allowed to validate many predictions. Actually, most of the cited drugs targeting human proteins were predicted by the model (Supplementary Table S3). A list of the drugs targeting some of the most relevant KDTs can be found in Supplementary Table S4. A detailed functional analysis of the circuits affected by the KDTs corresponding to these drugs, selected by the hallmarks they affect and the SHAP relevance, illustrates the different drug mechanisms of action in terms of how different COVID-19 hallmarks are potentially affected (see Fig. 1c) See Supplementary Fig. S3 and Supplementary Results for a detailed discussion of the findings.

The results presented here, although promising, can be considered only a subset of the potential drug candidates for repurposing, given that the detailed definition of the COVID-19 disease map is still an ongoing effort.^3^ As the map is updated, new reanalysis can render more interesting drug candidates for repurposing. It is worth mentioning that this approach can be used to search for drugs that tailor highly specific interventions over particular disease hallmarks or even particular signaling circuits, which would eventually help in the reduction of undesirable side-effects shown by some drugs.

Finally, the use of mechanistic models for drug repurposing has an extra advantage: it is well-known that the rate of success in drug discovery is of ~10% because many drugs fail in the last phases of clinical trials due to a lack of knowledge of the disease mechanism as well as problems of toxicity and bioavailability.^5^ Repurposing solves the last two problems and mechanistic modeling solves the first one by providing the biological knowledge, in terms of the mechanistic link between the drug and the effect on the disease, that other repurposing methodologies lack.

## Supplementary information

Supplementary Materials

## Data Availability

The datasets used during the current study are available in the GTEx repository, https://www.gtexportal.org/home/, the DrugBank database https://www.drugbankplus.com/, and the KEGG database https://www.genome.jp/kegg/. The COVID-19 Disease Map used here is available at 10.5281/zenodo.3935733, and new versions will be made publicly available at 10.17881/covid19-disease-map. A R/Bioconductor package for the Hipathia method is available at https://www.bioconductor.org/packages/release/bioc/html/hipathia.html.
