# Supplementary material for: Drug repurposing for COVID-19 using machine learning and mechanistic models of signal transduction circuits related to SARS-CoV-2 infection
Source: Signal Transduct Target Ther. 2020 Dec 11;5:290. doi: 10.1038/s41392-020-00417-y (PMC7729141; doi:10.1038/s41392-020-00417-y)
Supplement: Supplementary file 1 — Supplementary Materials [file 41392_2020_417_MOESM1_ESM.docx]

Supplementary Materials for

Drug repurposing for COVID-19 using machine learning and mechanistic models of signal transduction circuits related to SARS-CoV-2 infection

Carlos Loucera, Marina Esteban-Medina, Kinza Rian, Matías M Falco, Joaquín Dopazo, María Peña-Chilet

Correspondence to: [joaquin.dopazo@juntadeandalucia.es](mailto:joaquin.dopazo@juntadeandalucia.es)

**This PDF file includes:**

Materials and Methods

Supplementary Text

Figures. S1 to S3

Tables S1 to S4

Materials and Methods

Data

A list of all human proteins that interact with SARS-CoV-2 proteins has recently been published ^1^. There, the authors cloned, tagged and expressed 26 out of the 29 viral proteins from SARS-CoV-2 in human cells and identified those human proteins physically associated with the viral ones using affinity-purification mass spectrometry, resulting in a total of 332 protein-protein interactions (PPIs).

Gene expression data from 53 tissue sites across nearly 1000 non-diseased individuals, more than 11.000 samples and about 20.000 gene expression measurements each, were downloaded from the GTEx Portal ^2^ (GTEx Analysis V7; dbGaP Accession phs000424.v7.p2).

Genes that are targets of approved drugs were taken from the DrugBank ^3^ database (Version 5.1.4). A total of 2045 known drug target (KDT) proteins targeted by a total of 1735 drugs were considered in this study (see Supplementary Table S1). Some of these gene products may directly participate in COVID-19 related signaling circuits or affect them by means of a yet undescribed way. Their perturbations by the drug will cause quantitatively measurable perturbations in the cell functionalities triggered by the affected circuits.

RNA-seq data processing

A matrix of gene expression was constructed for all samples downloaded from the GTEx portal. The following pipeline was sequentially applied to the matrix: i) Trimmed mean of M values (TMM) normalization (*edgeR* package) ^4^, ii) Logarithm transformation (apply log(matrix+1)), iii) Truncation by the quantile 0.99 (all values greater than quantile 0.99 are truncated to this value, all values lower than quantile 0.01 are truncated to this other value) and finally iv) Quantiles normalization (*preprocessCore* package) ^5^.

Construction of a COVID-19 disease map

The recent publication of an exhaustive catalog of viral-human protein interactions ^6^ has enabled the construction of a first version of a map of the human molecular pathways involved in the COVID-19 viral infection. To do so, the human proteins from the SARS-CoV-2 virus-human interactome were firstly mapped onto the KEGG signaling pathways ^7^. This allows selecting the subset of human signaling circuits in which the virus directly interferes or influences in some way. In this modeling framework, a circuit is defined as the sub-pathway that contains the proteins that compose the chain of signal transduction that connects a receptor protein to an effector protein. This collection of circuits, arranged by pathways membership (for the sake of interpretability) represents a comprehensive map of the direct and indirect interactions of the virus with the cell and the most relevant downstream consequences (mostly, COVID-19 hallmarks). Finally, signaling circuits with at least one UniProt function that fit in one of the following virus-related categories were selected among them: 1) Host-virus interaction, 2) Inflammatory response, 3) Immune activity, 4) Antiviral defense, 5) Endocytosis. This first COVID-19 infection map, composed of 277 circuits, from a total of 49 KEGG pathways (see Supplementary Table S1), is part of an ongoing common international effort, that is generating an increasingly detailed repository of SARS-CoV-2 mechanism ^8^, the COVID-19 Disease Map used here is available ^9^ and new versions will be made public in the COVID-19 disease map consortium portal ^8^. Regarding the functional representation within the COVID-19 map, Synthesis and replication was the functionality triggered by most circuits (138 circuits), followed by Host-virus interaction (61), Antiviral defense (48 circuits), Inflammatory response and Energetics (both 39 circuits), Immune activity (35 circuits) and Endocytosis (17 circuits).

Mechanistic model of the COVID-19 map

The normalized gene expression data was rescaled from the range of variation to 0–1 interval range [max(matrix)=1, min(matrix)=0]. The *Hipathia* mechanistic modeling method ^10^, as implemented in the *Hipathia* Bioconductor package ^11^, was used to estimate signaling circuit activities within the COVID-19 map from the corresponding normalized gene expression values.

The Hipathia algorithm takes a directed graph (in this case the COVID-19 map), and extracts from it the collection of signaling circuits that connect receptor nodes to effector nodes, along with the corresponding activating or inhibiting activities. The signal transduction across such circuits is estimated from gene expression values using the following recursive equation ^10^:

| $S_{n}=\upsilon_{n}\cdot\left( 1-\prod_{s_{a}\in A} \left( 1-s_{a} \right) \right)\cdot\prod_{s_{i}\in I} \left( 1-s_{i} \right)$ | (1) |
| --- | --- |

*S_n_* being the signal intensity for the current node *n*, *v_n_* being its normalized gene expression value, *A* being the set of activating signals (*s_a_*) arriving to the current node from activation edges, *I* being the set of inhibiting signals (*s_i_*) arriving to the node from inhibition edges.

In this way, gene expression profiles can be transformed into estimated signaling activity profiles, in this case corresponding to the 277 circuits that compose the current version of the COVID-19 infection disease map ^9^.

Machine learning

In order to infer the relationships between the KDT proteins and the signaling circuits from the current COVID-19 disease map we will use a Multi-task Learning (MTL) model approach that tries to learn those relationships and the task interdependencies in an all-at-once fashion. In particular, a Multi-Output Random Forest (MORF) regressor, an ensemble of decision trees where each tree is built on a bootstrap sample of the training data ^12^, is used here. This strategy allows capturing both, the inherent relationships among the signaling circuits in the disease map at the output layer, as well as the expectable synergic relationships among KDT proteins at the input layer. Then, to determine the specific influences of KDT proteins on specific circuits within this complex network of relationships, SHapley Additive exPlanations (SHAP) ^13^ were used. SHAP is a novel method to find robust feature attribution scores for any given model, with a specific option to exploit the properties of tree-based models ^14^. Since SHAP values are additive, individual KDT relevance values for each specific circuit, triggering a specific COVID-19 hallmark, can be obtained. Moreover, the computed relevance values can be joined by groups or the whole dataset to find group-specific or global patterns, which is an especially interesting property in drug repurposing, since the SHAP values can be aggregated by drug (one drug can be targeting more than one protein).

In order to trust the model from a data-driven point of view, a sophisticated hyperparameter and feature selection three-steps process, that keeps the number of features low enough to make an interpretable model but still guaranteeing a high and stable predictive power, has been followed. The process i) searches the MORF hyperparameter space by means of the Tree of Parzen Estimators (TPE) optimization algorithm ^15^, by maximizing the R² score across a tenfold cross-validation, ii) asserts the predictive performance of the model by retraining and scoring the model using an independent CV schema and iii) performs a stability analysis of the feature selected by thresholding the SHAP feature ranking ^16^. This procedure constitutes a good compromise between exploration (global optimization) and exploitation (local optimization). Here, feature selection consists of finding a sparse subset of KDT proteins that regulates as many circuits as possible from the whole disease map, selecting those KDT that appear in the top decile of at least one circuit. The per-circuit ranking is obtained by computing the sample-wise mean of the absolute SHAP values. Since the input space is highly correlated, a known source of problems for feature selection and ranking methodologies ^17^, the stability of the solutions proposed is validated by subsampling random partitions of the training dataset as described elsewhere ^16^. Although a precise solution does not exist, as there is a tradeoff between the number of features selected, its stability and the predictive performance, values of the score above 0.4 represents a good enough agreement, as empirically proven ^16^.

All machine learning methods have been implemented on top of python 3.7 and *scikit-learn* general Machine Learning library ^18^, the *hyperopt* ^19^ package for hyperparameter optimization and the *SHAP* package ^13^ for SHAP value computation. More specifically, TreeShap ^14^, an algorithm to compute a SHAP value approximation that exploits the hierarchical structure of the trained trees in order to speed up the computation, was used to infer per-circuit attributions.

Enrichment analysis of most relevant genes

Genes with a relevance confirmed by the feature selection procedure were considered relevant and were used to perform an enrichment analysis to evaluate the nature of the potential causal effect over the circuits of the COVID-19 disease map. The enrichment analysis was carried out with the *enrichR* algorithm using GO Biological Processes ^20^.

Supplementary Text

Exploring the influence that druggable proteins have over the COVID-19 disease map

As sketched in Figure 1A, a Multi-task Learning (MTL), also known as multivariate or multi-output modeling, machine learning strategy was applied to detect KDT proteins whose activity at gene expression level predicts the signaling activity of the circuits of the COVID-19 disease map. The input space was restricted to a total of 2045 proteins, that are known targets of a total of 1735 drugs extracted from DrugBank, and the output space consists of the 277 signaling circuits (Supplementary Table S1) that compose the COVID-19 disease map above mentioned. Both, the predictive performance and the stability of the machine learning model used was measured. Firstly, the predictive power was measured by performing 10 times repeated tenfold cross-validation of the regression model. Here, the R² score, which goes from -∞ (arbitrarily wrong) to 1 (perfect match), being 0 the baseline (predicting the mean), was measured over all the test sets. The model scored a mean R² of 0.92 with a standard deviation (SD) of 0.015, resulting in a highly predictive model. Secondly, in order to measure the robustness of the feature selection technique used, which allows inferring specific KDT-circuit relationship strengths, a novel stability measure ^16^ was applied. The statistical treatment considers the stability of the feature selection method with respect to data resampling and the stochastic nature of the learning process. Therefore, the dataset was divided into 100 random subsamples of half the total sample size of our dataset, defining thus 100 training/test splits, and the feature selection was applied with respect to each partition. In order to have unbiased SHAP values, each training set was further subdivided into a learning/validation split where the MORF was trained in the learning split and the SHAP values were computed using the validation set. In this way, a 95%-interval for the stability measure could be estimated, which resulted in a score of 0.732 with a Confidence interval of [0.729, 0.735]. Note that it is typically advised that a score above 0.7 is extremely good ^16^. Furthermore, the MORF was also retrained over each training set, using only those KDT selected at each iteration, and scored the resulting model against the test set. This provided a new estimation of the predictive performance under such a strenuous splitting schema: a mean R² of 0.82 with a standard deviation of 0.02. Note that, as the training was done with less data (half the sample size against 9/10 of CV) the score is high while preserving a low variance, which is a good indicator of the noise filtering carried out by the feature selection procedure. Therefore, this approach renders a very trustable model.

Subsequently, the model was used to evaluate the impact of a KDT on specific circuits, and thus the corresponding cell functionalities triggered by them, by splitting the whole dataset into a train/test split of size 0.7/0.3 and applying the feature selection procedure based on the TreeSHAP method ^14^. The results showed that 380 out of 2045 original KDTs have direct relevant influence on at least one circuit (Supplementary Table S2, see also Supplementary Figure S1). Interestingly, due to the additivity inherent to SHAP values (see Supplementary Methods), clustering reveals three major KDT categories according to their influences: i) those with global influence on all the circuits, ii) those with strong influence on one or a few circuits and iii) those that influence a set of circuits related by a KEGG pathway (See Supplementary Figure S1).

Enrichment analysis with enrichR renders significant GO biological processes related to immune activity, especially to T-cell, but also to inflammatory response, and other infectious processes, such as hepatitis, HIV and papillomavirus infections. Moreover, GO functionalities corresponding to all COVID-19 hallmarks are represented as well. Interestingly, the enrichment in virus-caused perturbations in GEO expression showed an enrichment in datasets infected with SARS-CoV and other respiratory-related infections (Supplementary Figure S2).

Functional analysis of the relevant KDTs

With the aim of evaluating from a hallmark-centric perspective the potential effect of each KDT, the proportion of circuits affected was estimated for any COVID-19 functional hallmark. As portrayed in Supplementary Figure S3A, there is a group of KDTs relevant to all of the COVID-19 hallmarks. Some examples of these KDTs are *CACNA1B, IL6, MYC, PIK3CB, NTRK1* and *RYR1*, that correspond to KDTs simultaneously affecting most of the circuits that define the COVID-19 map. Additionally, a KDT-centric perspective of the results found was represented in Supplementary Figure S3B. For any KDT, the percentage of circuits that belong to each hallmark from the total number of circuits where that KDT is relevant was calculated. It is remarkable that the most represented cell process is “Replication”, with a cluster of KDTs relevant for circuits highly tagged with this function. Some KDTs within this cluster are *FGFR1*, *PRKCI*, *TOP1* and *BIRC5*, among others. The second most represented function is “Host-virus interaction”. Interestingly, most of the KDTs within this category are also involved in “Replication”. It is worth mentioning that KDT proteins like *mTOR* and *IKBKB* are known to be associated with pathways involved in SARS-CoV-2 infection, among others. Furthermore, under the hallmark “Energetics” we can see another well-defined cluster of highly enriched KDTs, such as *GPRC5A* and *ELOVL4*. The hallmark “Endocytosis” showed an enrichment in KDTs like *FGFR3* and *HNRNPH1* among others. In addition, under the hallmark “Antiviral Defense” we can find enriched KDT proteins like *DPP4*, *BTK*, *SERPING1* and *IL2RA*, as well as *CYP1B* and *NDUFS4*, which are also involved in the “Immune activity” cell function. Finally, the hallmark “Inflammatory response” is enriched in KDTs like *SCN3A*, *ACVR1B* and *MAPK13*, among others.

Most relevant drugs proposed

Among the drugs predicted to have a relevant effect, some of them are currently under clinical trials. Interestingly, these drugs define different functional profile templates, which may be useful to speculate similar consequences for other drugs with similar patterns of influence over the signaling circuits that define the disease hallmarks. Thus, some drugs, such as Chloroquine and Sirolimus, have a strong impact over most of the circuits, while other as Hydroxychloroquine (9 circuits) and Ciclosporin (7 circuits) affect only a small number of circuits (Figure 1B). Indeed, Hydroxychloroquine and Ciclosporin, both share circuits within Toll-like and adipocytokine signaling pathways. Moreover, Hydroxychloroquine is relevant for Inflammatory mediator regulation of TRP channels and HIF-1 signaling pathways, while Insulin signaling is influenced by Ciclosporin. Chloroquine and Ciclosporin are representative of two different modes of action by either affecting massively to almost all COVID-19 disease map circuits or only affecting a few specific ones, respectively. A list of the drugs targeting some of the most relevant KDTs can be found in Table 1. A detailed functional analysis of the circuits affected by the KDTs corresponding to the drugs in Table 1, selected by the hallmarks they affect and the SHAP relevance, illustrates the different drug mechanisms of action in terms of how different COVID-19 hallmarks are potentially affected (see Figure 1C).

Relevant targets and the corresponding drugs

The approach used here discovers KDTs with a relevant influence either in a portion or in the whole COVID-19 disease map. The drugs targeting these proteins are, consequently, potentially repurposable drugs. Interestingly, the most relevant KDT proteins are significantly enriched in functional annotations related to virus infection, but mostly with immune system activation and inflammation response. Moreover, the enrichment analysis using disease-related labels rendered a significant overabundance of annotations that are concordant with observed systemic inflammatory response symptoms ^21^. Furthermore, the immune activity triggered by SARS-CoV-2 infection seems to be over-represented in T-cell mechanisms (See Supplementary Figure S2), in concordance with very recent findings ^22^. Notably, some of the infectious processes enriched in the candidate KDTs are similar to those of SARS-CoV infection, suggesting that they share most of the cell response processes involved, if confirmed, these findings could be extremely helpful in the study of SARS-CoV-2, given that it has been a long way since the SARS-CoV outbreak back in 2001 ^23^.

It is worth noting that there are several targets of drugs that are already ongoing clinical trials for its potential treatment for COVID-19 among the relevant KDTs selected by the model, which provide extra empirical support for the results obtained. Some of the drugs found by this approach as promising candidates deserve a special comment given its current impact in clinical practice and the number of studies evaluating its efficacy as a COVID-19 treatment. Drugs targeting *CES1* gene product, such as oseltamivir, *TLR9* or *TLR7,* like hydroxychloroquine or *NR1L2,* like ritonavir are all currently under clinical trials against the disease and, despite some of them being controversial, with relatively promising results ^24,25^. Moreover, the model also suggests drugs targeting *IL-6*, like tocilizumab, which has also recently been included in clinical trials against SARS-CoV-2 infection and it is currently undergoing phase 2 with satisfactory preliminary results ^26,27^.

However, a further evaluation from a clinical point of view is encouraged, since drugs can have a wide variety of mechanisms of action, side effects and incompatibilities with symptoms that need to be considered in the COVID-19 context.

Interpretability of the results found

The most affected functionalities of the COVID-19 disease map by the relevant drugs were related to *virus replication*, including all the *synthesis machinery* ^28^, followed by *host-virus interaction*, *antiviral defense* and *cell energetics*, such as glucose metabolism (needed to maintain the replication rate of the infected cells). All these functions are characteristic of an infection process ^29^ and drugs affecting these circuits would probably have a general effect against all virus infections, as for example, the *MAP signaling pathway:CDC25B* circuit, which is also affected by the influenza virus ^30^. However, we found a set of targets affecting specifically inflammatory response and innate immune activity, like *NF-kappa B signaling pathway:CXCL8* or *Toll-like receptor signaling pathway:CD40* circuits, that have been established as processes highly deregulated in COVID-19, and suggested to be causative of the disproportionate symptomatology observed in some patients ^31-33^. Therefore, targeting these mechanisms emerge as a promising way of counteracting the disease progression to severe symptoms.

One of the KDTs with a relevant effect over Replication and Host-virus interaction COVID-19 hallmarks is *IKBKB*, targeted by auranofin and fostamatinib. Drugs targeting *IKBKB* are good candidates for drug repositioning, given its involvement in NF-KB signaling pathway and its suggested deregulation in COVID-19 ^34,35^ .

Several of the KDTs obtained by the model (such as *IL6, IL1B, IL4R, IL1R1, IL2R, IL3RA*, among others) are part of the interleukin family, a group of cytokines that trigger immune activity and inflammatory response, processes involved in the worsening of COVID-19 symptoms ^32^. Thus, targeting the action of these processes by targeting interleukin or their receptors could be a promising approach (https://clinicaltrials.gov/ct2/show/NCT04335071) ^31^. We have highlighted the hallmarks of some of the drugs targeting interleukins (such as basiliximab, dupilumab or tocilizumab) all together with some of the drugs already used against COVID-19 (Figure 4).

Use of causality provided by mechanistic models in the context of machine learning

The use of mechanistic modeling of COVID-19 disease map in combination with machine learning methodologies oriented to drug repurposing, highlights the potential of genomic *Big Data* analysis in the field of drug discovery ^36^ and, in particular, for drug repurposing. Interestingly, the vast amount of transcriptomic data available, which ultimately encode information on the relationships between the genes transcribed, can be used to build new biological knowledge over the already modeled cell functionality by using machine learning. Specifically, thousands of gene expression data have been used here by a machine learning method to learn the relationships between the behavior of proteins of interest, KDTs, and the activity status of the different signaling circuits of the COVID-19 map that ultimately trigger the disease hallmarks. Another advantage of this approach is that new, improved versions of the COVID-19 disease map arising in the future ^8^ can easily be modeled and used to recalculate KDTs influences that could render new potential repurposable drugs more precise towards specific COVID-19 hallmarks.

On the other hand, although machine learning is playing an increasingly important role in drug discovery ^36^, to our knowledge, the use of human genomic data aiming to find genes potentially causal of phenotypes have been restricted to a few cases, such as the inference of univariate phenotypes, e.g. the activity status of Ras pathway in cancer ^37^. The approach taken here aims to tackle the pathologic phenotype problem in more detail, trying to capture the complexity of the molecular mechanism of the disease. To achieve so, the signaling circuit activities inferred by mechanistic models have been used as proxies of disease-related cell functionalities, or COVID-19 hallmarks, triggered by them. Then, in the proposed approach, KDT activities (inferred from gene expression values) are linked to COVID-19 hallmarks (inferred by the mechanistic model) in a way that the complex relationships among KDTs and COVID-19 hallmarks are captured. In a conventional regression approach independent KDT-circuit models could have been obtained. However, the resulting conventional regressors would violate the map interconnectivity hypothesis within the pathway, since each model could only account for the circuit it has been trained for, losing crucial information about the rest of the disease map. Here, an MTL regressor was used, with an input space of 2045 KDT proteins and an output space consisting of the 277 signaling circuits that conform the COVID-19 disease map. The MTL regressor used, a Multi-Output Random Forest ^12^ that efficiently accounts for the complex relationships among the input and the output variables, along with the efficient strategy for feature selection, resulted in a reduced collection of 295 KDTs with a relevant effect over the COVID-19 disease map activity (See Supplementary Table S4). Another novel machine learning methodology is SHAP ^13,38^, that can be used on any given model to find robust feature attribution scores, and is especially efficient in exploiting the properties of tree-based models ^14^. SHAP allowed to define the specific circuits over which any particular KDT has significant influence (Supplementary Table S4).

References

1 Gordon, D. E. *et al.* A SARS-CoV-2 protein interaction map reveals targets for drug repurposing. *Nature*, (2020).

2 Lonsdale, J. *et al.* The genotype-tissue expression (GTEx) project. *Nature genetics*. **45**, 580, (2013).

3 Wishart, D. S. *et al.* DrugBank 5.0: a major update to the DrugBank database for 2018. *Nucleic acids research*. **46**, D1074-D1082, (2017).

4 Robinson, M. D., McCarthy, D. J. & Smyth, G. K. edgeR: a Bioconductor package for differential expression analysis of digital gene expression data. *Bioinformatics*. **26**, 139-140, (2010).

5 Bolstad, B. M., Irizarry, R. A., Åstrand, M. & Speed, T. P. A comparison of normalization methods for high density oligonucleotide array data based on variance and bias. *Bioinformatics*. **19**, 185-193, (2003).

6 Gordon, D. E. *et al.* A SARS-CoV-2-Human Protein-Protein Interaction Map Reveals Drug Targets and Potential Drug-Repurposing. *BioRxiv*, (2020).

7 Kanehisa, M. *et al.* KEGG as a reference resource for gene and protein annotation. *Nucleic acids research*. **44**, D457-D462, (2015).

8 Ostaszewski, M. *et al.* COVID-19 Disease Map, building a computational repository of SARS-CoV-2 virus-host interaction mechanisms. *Scientific Data*. **7**, 136, (2020).

9 Loucera, C. *et al.* (Zenodo, 2020).

10 Hidalgo, M. R. *et al.* High throughput estimation of functional cell activities reveals disease mechanisms and predicts relevant clinical outcomes. *Oncotarget*. **8**, 5160-5178, (2017).

11 *HiPathia: High-throughput Pathway Analysis*, <<http://bioconductor.org/packages/release/bioc/html/hipathia.html>> (2019).

12 Segal, M. & Xiao, Y. Multivariate random forests. *Wiley Interdisciplinary Reviews: Data Mining and Knowledge Discovery*. **1**, 80-87, (2011).

13 Lundberg, S. M. & Lee, S.-I. in *Advances in Neural Information Processing Systems.* 4765-4774.

14 Lundberg, S. M. *et al.* From local explanations to global understanding with explainable AI for trees. *Nature machine intelligence*. **2**, 2522-5839, (2020).

15 Bergstra, J. S., Bardenet, R., Bengio, Y. & Kégl, B. in *Advances in neural information processing systems.* 2546-2554.

16 Nogueira, S., Sechidis, K. & Brown, G. On the stability of feature selection algorithms. *The Journal of Machine Learning Research*. **18**, 6345-6398, (2017).

17 Toloşi, L. & Lengauer, T. Classification with correlated features: unreliability of feature ranking and solutions. *Bioinformatics*. **27**, 1986-1994, (2011).

18 Pedregosa, F. *et al.* Scikit-learn: Machine learning in Python. *Journal of machine learning research*. **12**, 2825-2830, (2011).

19 Bergstra, J., Yamins, D. & Cox, D. in *International Conference on Machine Learning.* 115-123.

20 Kuleshov, M. V. *et al.* Enrichr: a comprehensive gene set enrichment analysis web server 2016 update. *Nucleic Acids Research*. **44**, W90-W97, (2016).

21 Fu, L. *et al.* Clinical characteristics of coronavirus disease 2019 (COVID-19) in China: a systematic review and meta-analysis. *Journal of Infection*, (2020).

22 Chen, Y. & Li, L. SARS-CoV-2: virus dynamics and host response. *The Lancet infectious diseases*. **20**, 515-516, (2020).

23 Petersen, E. *et al.* Comparing SARS-CoV-2 with SARS-CoV and influenza pandemics. *The Lancet infectious diseases*, (2020).

24 Rosa, S. G. V. & Santos, W. C. Clinical trials on drug repositioning for COVID-19 treatment. *Revista Panamericana de Salud Pública*. **44**, e40, (2020).

25 Mehra, M. R., Desai, S. S., Ruschitzka, F. & Patel, A. N. Hydroxychloroquine or chloroquine with or without a macrolide for treatment of COVID-19: a multinational registry analysis. *The Lancet*, (2020).

26 Xu, X. *et al.* Effective treatment of severe COVID-19 patients with tocilizumab. *Proceedings of the National Academy of Sciences*. **117**, 10970-10975, (2020).

27 Luo, P. *et al.* Tocilizumab treatment in COVID‐19: A single center experience. *Journal of medical virology*, (2020).

28 Walsh, D. & Mohr, I. Viral subversion of the host protein synthesis machinery. *Nature Reviews Microbiology*. **9**, 860-875, (2011).

29 Sanchez, E. L. & Lagunoff, M. Viral activation of cellular metabolism. *Virology*. **479**, 609-618, (2015).

30 Geiss, G. K. *et al.* Global impact of influenza virus on cellular pathways is mediated by both replication-dependent and-independent events. *Journal of virology*. **75**, 4321-4331, (2001).

31 Jose, R. J. & Manuel, A. COVID-19 cytokine storm: the interplay between inflammation and coagulation. *The Lancet Respiratory Medicine*, (2020).

32 Mehta, P. *et al.* COVID-19: consider cytokine storm syndromes and immunosuppression. *The Lancet*. **395**, 1033-1034, (2020).

33 Sun, X. *et al.* Cytokine storm intervention in the early stages of COVID-19 pneumonia. *Cytokine & growth factor reviews*, (2020).

34 Ziegler, C. G. *et al.* SARS-CoV-2 receptor ACE2 is an interferon-stimulated gene in human airway epithelial cells and is detected in specific cell subsets across tissues. *Cell*, (2020).

35 Utay, N. S. & Douek, D. C. Interferons and HIV infection: the good, the bad, and the ugly. *Pathogens & immunity*. **1**, 107, (2016).

36 Chen, H. *et al.* The rise of deep learning in drug discovery. *Drug discovery today*. **23**, 1241-1250, (2018).

37 Way, G. P. *et al.* Machine learning detects pan-cancer ras pathway activation in the cancer genome atlas. *Cell reports*. **23**, 172-180. e173, (2018).

38 Lundberg, S. M. *et al.* Explainable machine-learning predictions for the prevention of hypoxaemia during surgery. *Nature biomedical engineering*. **2**, 749, (2018).

39 Fragkou, P. C. *et al.* Review of trials currently testing treatment and prevention of COVID-19. *Clinical Microbiology and Infection*, (2020).


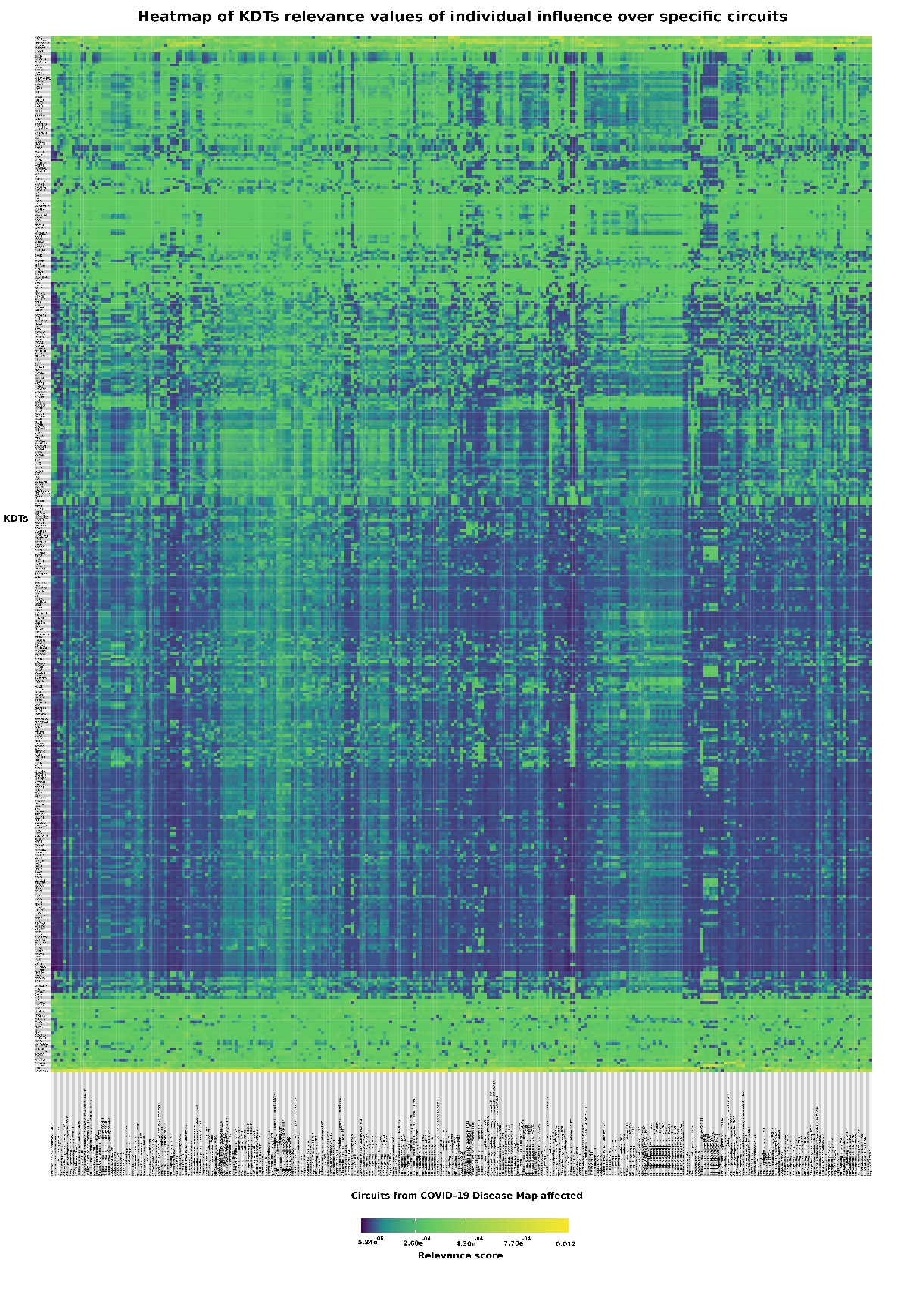


Figure. S1.

Heatmap of KDTs relevance values of individual influence over specific circuits (from Supplementary Table S4).


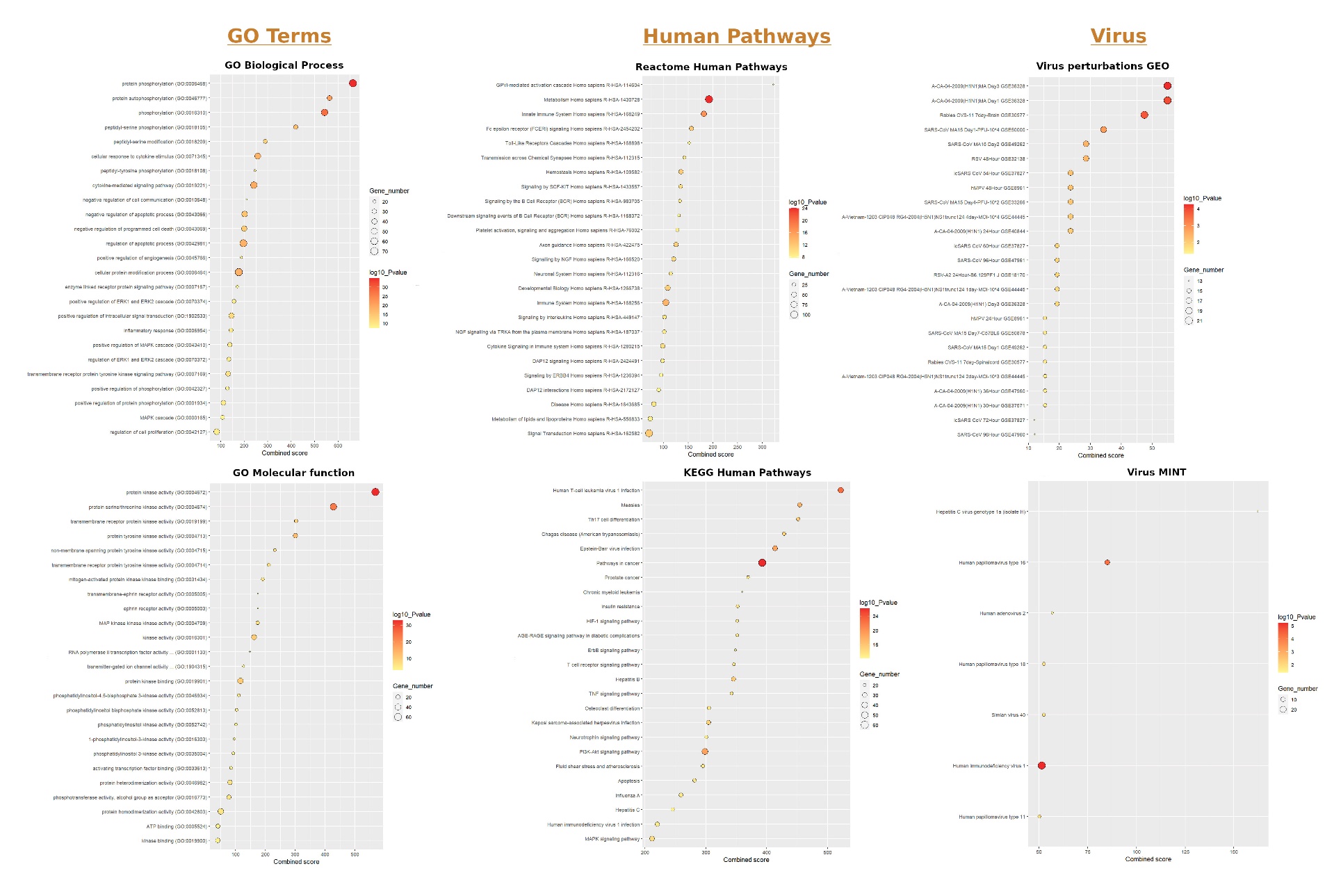


Figure. S2.

Type or paste caption here. Create a page break and paste in the Figure above the caption.


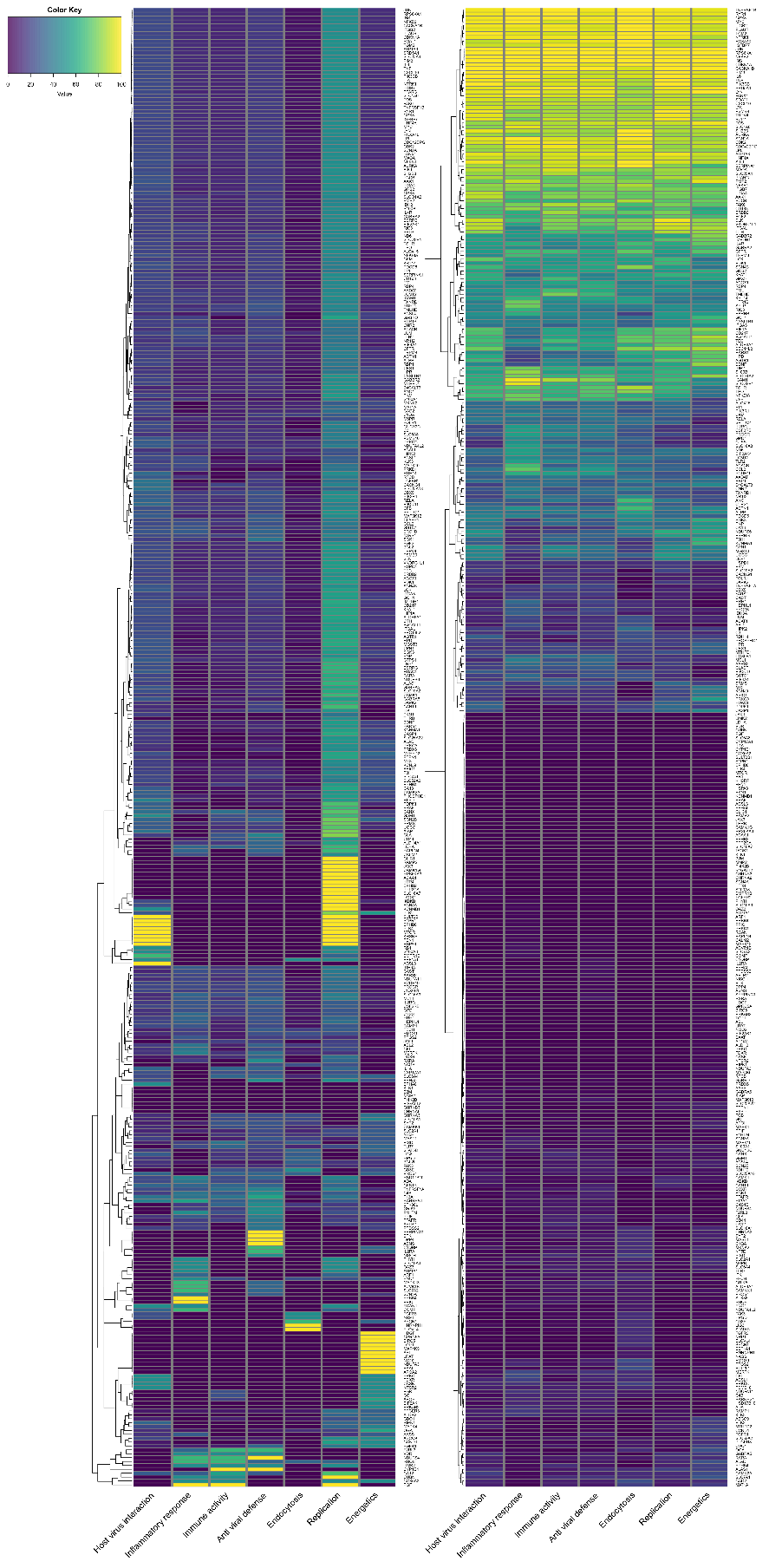


Figure. S3.

Type or paste caption here. Create a page break and paste in the Figure above the caption.

Table S1.

Circuits that conform the version of the COVID-19 disease map used here and the COVID-19 hallmarks they are related to.

| **Circuit** | **Host-virus interaction** | **Inflammatory response** | **Immune activity** | **Anti-viral defense** | **Endocytosis** | **Replication** | **Energetics** |
| --- | --- | --- | --- | --- | --- | --- | --- |
| MAPK signaling pathway: NFKB1 | Y |  |  |  |  | Y |  |
| MAPK signaling pathway: ATF4 |  |  |  |  |  | Y |  |
| MAPK signaling pathway: HSPB1 |  |  |  | Y |  |  |  |
| MAPK signaling pathway: ATF2 |  |  |  | Y |  | Y |  |
| MAPK signaling pathway: ELK1 |  |  |  |  |  | Y |  |
| MAPK signaling pathway: TP53 | Y |  |  |  |  | Y |  |
| MAPK signaling pathway: ELK4 |  |  |  |  |  | Y |  |
| MAPK signaling pathway: DDIT3 |  |  |  | Y |  | Y |  |
| MAPK signaling pathway: MAX |  |  |  |  |  | Y |  |
| MAPK signaling pathway: MEF2C |  |  |  |  |  | Y |  |
| MAPK signaling pathway: CDC25B |  |  |  |  |  | Y |  |
| MAPK signaling pathway: JUN |  |  |  |  |  | Y |  |
| Ras signaling pathway: BCL2L1 |  |  |  |  | Y |  |  |
| Ras signaling pathway: NFKB1 | Y |  |  |  |  | Y |  |
| cAMP signaling pathway: BDNF |  |  |  |  |  | Y |  |
| cAMP signaling pathway: FOS |  | Y |  | Y |  |  |  |
| cAMP signaling pathway: JUN |  |  |  |  |  | Y |  |
| cAMP signaling pathway: CREB3 CREBBP | Y |  |  |  |  |  |  |
| Chemokine signaling pathway: STAT1 | Y |  |  | Y |  | Y |  |
| Chemokine signaling pathway: NFKB1 | Y |  |  |  |  | Y |  |
| Chemokine signaling pathway: GSK3A |  |  |  |  |  | Y | Y |
| NF-kappa B signaling pathway: CFLAR | Y |  |  |  |  |  |  |
| NF-kappa B signaling pathway: BIRC2 |  |  |  |  |  | Y |  |
| NF-kappa B signaling pathway: XIAP |  |  |  |  |  |  |  |
| NF-kappa B signaling pathway: BCL2L1 |  |  |  |  | Y |  |  |
| NF-kappa B signaling pathway: BCL2 |  |  |  |  |  |  |  |
| NF-kappa B signaling pathway: GADD45B |  |  |  |  |  | Y |  |
| NF-kappa B signaling pathway: TRAF1 |  |  |  |  |  |  |  |
| NF-kappa B signaling pathway: BCL2A1 |  |  |  |  |  |  |  |
| NF-kappa B signaling pathway: NFKB2 |  |  |  |  |  | Y |  |
| NF-kappa B signaling pathway: CXCL8 |  | Y |  | Y |  |  |  |
| NF-kappa B signaling pathway: IL1B |  | Y |  | Y |  |  |  |
| NF-kappa B signaling pathway: TNF |  | Y | Y | Y |  |  |  |
| NF-kappa B signaling pathway: TNFAIP3 |  |  |  |  |  |  |  |
| NF-kappa B signaling pathway: NFKBIA | Y |  |  |  |  |  |  |
| NF-kappa B signaling pathway: PTGS2 |  |  |  |  | Y |  |  |
| NF-kappa B signaling pathway: CCL4L1 |  | Y |  | Y |  |  |  |
| NF-kappa B signaling pathway: CXCL2 |  | Y |  | Y |  |  |  |
| NF-kappa B signaling pathway: VCAM1 | Y |  |  |  |  |  |  |
| NF-kappa B signaling pathway: BCL2L1 |  |  |  |  | Y |  |  |
| NF-kappa B signaling pathway: BCL2L1 |  |  |  |  | Y |  |  |
| NF-kappa B signaling pathway: BCL2A1 |  |  |  |  |  |  |  |
| HIF-1 signaling pathway: TIMP1 |  |  |  |  |  | Y | Y |
| HIF-1 signaling pathway: LTBR | Y |  |  |  |  |  |  |
| HIF-1 signaling pathway: EPO |  |  |  |  |  |  |  |
| HIF-1 signaling pathway: TF |  |  |  |  |  |  | Y |
| HIF-1 signaling pathway: TFRC | Y |  |  |  | Y |  |  |
| HIF-1 signaling pathway: VEGFA |  |  |  |  |  | Y |  |
| HIF-1 signaling pathway: FLT1 |  |  |  | Y |  | Y |  |
| HIF-1 signaling pathway: EGF |  |  |  |  |  | Y |  |
| HIF-1 signaling pathway: SERPINE1 |  | Y |  | Y | Y |  |  |
| HIF-1 signaling pathway: ANGPT1 |  |  |  |  |  | Y |  |
| HIF-1 signaling pathway: TEK |  |  |  |  |  |  |  |
| HIF-1 signaling pathway: TIMP1 |  | Y |  |  |  |  | Y |
| HIF-1 signaling pathway: EDN1 |  |  | Y | Y |  |  |  |
| HIF-1 signaling pathway: NOS2 |  |  |  |  |  | Y |  |
| HIF-1 signaling pathway: NOS3 |  |  |  |  |  | Y |  |
| HIF-1 signaling pathway: HMOX1 |  |  |  |  |  |  |  |
| HIF-1 signaling pathway: NPPA |  | Y | Y |  |  | Y |  |
| HIF-1 signaling pathway: SLC2A1 |  |  |  |  |  |  | Y |
| HIF-1 signaling pathway: PDK1 |  |  |  |  |  |  | Y |
| HIF-1 signaling pathway: HK1 |  |  |  |  |  |  | Y |
| HIF-1 signaling pathway: PFKL |  |  |  |  |  |  | Y |
| HIF-1 signaling pathway: GAPDH |  |  |  |  |  |  | Y |
| HIF-1 signaling pathway: ALDOA |  |  |  |  |  |  | Y |
| HIF-1 signaling pathway: PGK1 |  |  |  |  |  |  | Y |
| HIF-1 signaling pathway: PFKFB3 |  |  |  |  |  |  | Y |
| HIF-1 signaling pathway: LDHA |  |  |  |  |  |  | Y |
| HIF-1 signaling pathway: BCL2 |  |  |  |  |  |  |  |
| HIF-1 signaling pathway: CDKN1A |  |  |  |  |  | Y |  |
| HIF-1 signaling pathway: ENO1 |  |  |  |  |  | Y | Y |
| Sphingolipid signaling pathway: TP53 BCL2 | Y |  |  |  |  | Y |  |
| Cell cycle: RB1 | Y |  |  |  |  | Y |  |
| Cell cycle: TFDP1 E2F4 |  |  |  |  |  | Y |  |
| Cell cycle: CDC6 ORC3 ORC5 ORC4 ORC2 ORC1 ORC6 |  |  |  |  |  | Y |  |
| mTOR signaling pathway: EIF4E | Y |  |  |  |  | Y |  |
| mTOR signaling pathway: EIF4B |  |  |  |  |  | Y |  |
| mTOR signaling pathway: RPS6 | Y |  |  |  |  |  |  |
| PI3K-Akt signaling pathway: GYS1 |  |  |  |  |  |  | Y |
| PI3K-Akt signaling pathway: MYC |  |  |  |  |  | Y |  |
| PI3K-Akt signaling pathway: CCND1 |  |  |  |  |  | Y |  |
| PI3K-Akt signaling pathway: BCL2L1 |  |  |  |  | Y |  |  |
| PI3K-Akt signaling pathway: BCL2 |  |  |  |  |  |  |  |
| PI3K-Akt signaling pathway: BCL2L1 |  |  |  |  | Y |  |  |
| PI3K-Akt signaling pathway: MYB |  |  |  |  |  | Y |  |
| PI3K-Akt signaling pathway: BCL2 |  |  |  |  |  |  |  |
| PI3K-Akt signaling pathway: MCL1 |  |  |  |  |  | Y |  |
| PI3K-Akt signaling pathway: BCL2 |  |  |  |  |  |  |  |
| Apoptosis: PARP2 |  |  |  |  |  | Y |  |
| Apoptosis: BAX | Y |  |  |  |  |  |  |
| Apoptosis: BAK1 |  |  |  |  |  |  |  |
| Apoptosis: PTPN13 |  | Y |  |  |  |  |  |
| Apoptosis: CFLAR | Y |  |  |  |  |  |  |
| Apoptosis: BIRC2 |  |  |  |  |  | Y |  |
| Apoptosis: GADD45G |  |  |  | Y |  | Y |  |
| Apoptosis: TRAF1 |  |  |  |  |  |  |  |
| Apoptosis: BCL2L1 |  |  |  |  | Y |  |  |
| Apoptosis: BCL2A1 |  |  |  |  |  |  |  |
| Apoptosis: BCL2 |  |  |  |  |  |  |  |
| Apoptosis: TP53 | Y |  |  |  |  | Y |  |
| Apoptosis: FAS |  |  |  |  | Y |  |  |
| Apoptosis: FASLG |  |  |  |  |  | Y |  |
| Apoptosis: BCL2L11 |  |  |  |  |  |  |  |
| Apoptosis: HRK |  |  |  |  |  |  |  |
| Apoptosis: MCL1 |  |  |  |  |  | Y |  |
| Adrenergic signaling in cardiomyocytes: ATP2A2 PLN |  |  |  |  |  |  | Y |
| Adrenergic signaling in cardiomyocytes: SCN1B |  |  |  |  |  |  | Y |
| Adrenergic signaling in cardiomyocytes: RYR2 |  |  |  |  |  |  | Y |
| Adrenergic signaling in cardiomyocytes: BCL2 |  |  |  |  |  |  |  |
| Wnt signaling pathway: MYC |  |  |  |  |  | Y |  |
| Wnt signaling pathway: JUN |  |  |  |  |  | Y |  |
| Wnt signaling pathway: FOSL1 |  | Y |  | Y |  | Y |  |
| Wnt signaling pathway: CCND1 |  |  |  |  |  | Y |  |
| Wnt signaling pathway: PPARD |  |  |  |  |  | Y |  |
| Wnt signaling pathway: MMP7 |  |  |  |  |  |  |  |
| Hedgehog signaling pathway: GLI1 |  |  |  |  |  | Y |  |
| Hedgehog signaling pathway: PTCH1 |  |  |  |  |  | Y |  |
| Hedgehog signaling pathway: HHIP |  |  |  |  |  | Y |  |
| Hedgehog signaling pathway: CCND1 |  |  |  | Y |  | Y |  |
| Hedgehog signaling pathway: BCL2 |  |  |  |  |  |  |  |
| Hedgehog signaling pathway: BCL2 |  |  |  |  |  |  |  |
| TGF-beta signaling pathway: RPS6KB1 |  |  |  |  |  | Y |  |
| TGF-beta signaling pathway: ROCK1 |  |  |  |  |  |  |  |
| TGF-beta signaling pathway: CDKN2B |  |  |  |  |  | Y |  |
| TGF-beta signaling pathway: SMAD4 |  |  |  |  |  | Y |  |
| TGF-beta signaling pathway: SMAD4 |  |  |  |  |  | Y |  |
| Focal adhesion: VASP ACTB |  |  |  |  | Y | Y |  |
| Focal adhesion: ZYX ACTB | Y |  |  |  |  |  |  |
| Focal adhesion: CCND1 |  |  |  |  |  | Y |  |
| Focal adhesion: BIRC2 |  |  |  |  |  | Y |  |
| Focal adhesion: BCL2 |  |  |  |  |  |  |  |
| Focal adhesion: BAD |  |  |  |  |  |  |  |
| Focal adhesion: VCL TLN1 | Y |  |  |  |  |  |  |
| Focal adhesion: TLN1 ITGB1 ITGA11 | Y |  |  |  |  | Y |  |
| Focal adhesion: VCL ACTN4 |  |  |  |  |  |  | Y |
| Adherens junction: SMAD4 SMAD2 |  |  |  |  |  | Y |  |
| Signaling pathways regulating pluripotency of stem cells: ESRRB |  |  |  |  |  | Y |  |
| Signaling pathways regulating pluripotency of stem cells: HNF1A |  |  |  |  |  | Y |  |
| Signaling pathways regulating pluripotency of stem cells: MYC |  |  |  |  |  | Y |  |
| Signaling pathways regulating pluripotency of stem cells: SMAD2 |  |  |  |  |  | Y |  |
| Signaling pathways regulating pluripotency of stem cells: SMAD2 SMAD4 |  |  |  |  |  | Y |  |
| Signaling pathways regulating pluripotency of stem cells: CTNNB1 |  |  |  |  |  | Y |  |
| Signaling pathways regulating pluripotency of stem cells: MYC |  |  |  |  |  | Y |  |
| Complement and coagulation cascades: C2 |  |  | Y |  |  |  |  |
| Complement and coagulation cascades: C4A |  |  | Y |  |  |  |  |
| Toll-like receptor signaling pathway: CXCL10 |  | Y |  |  |  |  |  |
| Toll-like receptor signaling pathway: CXCL11 |  | Y |  |  |  |  |  |
| Toll-like receptor signaling pathway: CXCL9 |  | Y |  |  |  |  |  |
| Toll-like receptor signaling pathway: IFNB1 |  |  |  | Y |  |  |  |
| Toll-like receptor signaling pathway: TNF |  | Y | Y | Y |  | Y |  |
| Toll-like receptor signaling pathway: IL1B |  | Y |  |  |  |  |  |
| Toll-like receptor signaling pathway: IL6 |  | Y | Y | Y |  |  |  |
| Toll-like receptor signaling pathway: IL12A |  |  | Y | Y |  | Y |  |
| Toll-like receptor signaling pathway: CXCL8 |  | Y |  | Y |  |  |  |
| Toll-like receptor signaling pathway: CCL5 |  | Y |  | Y |  |  |  |
| Toll-like receptor signaling pathway: CCL3L3 |  | Y |  | Y |  |  |  |
| Toll-like receptor signaling pathway: CCL4L1 |  | Y |  | Y |  |  |  |
| Toll-like receptor signaling pathway: NFKB1 |  |  |  |  |  | Y |  |
| Toll-like receptor signaling pathway: CD40 |  |  | Y |  |  |  |  |
| Toll-like receptor signaling pathway: CD80 | Y |  |  |  |  |  |  |
| Toll-like receptor signaling pathway: CD86 | Y |  | Y |  |  |  |  |
| RIG-I-like receptor signaling pathway: NFKB1 | Y |  |  |  |  | Y |  |
| RIG-I-like receptor signaling pathway: CHUK IKBKB IKBKG | Y |  |  |  |  | Y |  |
| RIG-I-like receptor signaling pathway: IRF3 PIN1 | Y |  | Y | Y |  | Y |  |
| Cytosolic DNA-sensing pathway: TBK1 | Y |  | Y | Y |  |  |  |
| Cytosolic DNA-sensing pathway: IKBKE |  |  |  | Y |  |  |  |
| Cytosolic DNA-sensing pathway: NFKB1 | Y |  |  |  |  | Y |  |
| T cell receptor signaling pathway: NFKB1 | Y |  |  |  |  | Y |  |
| T cell receptor signaling pathway: NFATC1 |  |  |  |  |  | Y |  |
| T cell receptor signaling pathway: FOS |  |  |  |  |  | Y |  |
| TNF signaling pathway: CASP7 |  |  |  |  |  |  |  |
| TNF signaling pathway: CASP3 |  |  |  |  |  |  |  |
| TNF signaling pathway: JUN |  |  |  |  |  | Y |  |
| TNF signaling pathway: CREB3 | Y |  |  |  |  | Y |  |
| TNF signaling pathway: NFKB1 | Y |  |  |  |  | Y |  |
| TNF signaling pathway: JUN |  |  |  |  |  | Y |  |
| Long-term potentiation: CREBBP ATF4 | Y |  |  |  |  | Y |  |
| Long-term potentiation: ITPR1 |  |  |  |  |  |  | Y |
| Neurotrophin signaling pathway: GSK3B |  |  |  |  |  | Y | Y |
| Neurotrophin signaling pathway: JUN |  |  |  |  |  | Y |  |
| Neurotrophin signaling pathway: NFKB1 | Y |  |  |  |  | Y |  |
| Inflammatory mediator regulation of TRP channels: TRPM8 |  |  |  |  |  |  | Y |
| Regulation of actin cytoskeleton: MYL12B MYH9 ACTB |  |  |  |  |  |  |  |
| Insulin signaling pathway: EIF4E | Y |  |  |  |  | Y |  |
| Insulin signaling pathway: RPS6 | Y |  |  |  |  |  |  |
| Insulin signaling pathway: ACACA |  |  |  |  | Y |  |  |
| Insulin signaling pathway: FASN |  |  |  |  | Y |  |  |
| Insulin signaling pathway: PKLR |  |  |  |  |  |  | Y |
| Insulin signaling pathway: GCK |  |  | Y | Y |  |  | Y |
| Insulin signaling pathway: D-Glucose |  |  |  |  |  |  | Y |
| Insulin signaling pathway: Starch |  |  |  |  |  |  | Y |
| Insulin signaling pathway: PPARGC1A |  |  |  |  |  | Y |  |
| Insulin signaling pathway: G6PC |  |  |  |  |  |  | Y |
| Insulin signaling pathway: FBP1 |  |  |  |  |  |  | Y |
| Insulin signaling pathway: PCK1 |  |  |  |  |  |  | Y |
| Insulin signaling pathway: LIPE |  |  |  |  | Y |  |  |
| Insulin signaling pathway: BAD |  |  |  |  |  |  |  |
| Insulin signaling pathway: ELK1 |  |  |  |  |  | Y |  |
| Adipocytokine signaling pathway: Fatty acid |  | Y | Y |  | Y |  |  |
| Adipocytokine signaling pathway: Long-chain fatty acid |  | Y |  |  |  | Y | Y |
| Adipocytokine signaling pathway: SLC2A1 |  |  |  |  |  |  | Y |
| Adipocytokine signaling pathway: PCK1 |  |  |  |  |  |  | Y |
| Adipocytokine signaling pathway: G6PC |  |  |  |  |  |  | Y |
| Adipocytokine signaling pathway: PTPN11 |  | Y |  |  |  |  |  |
| Adipocytokine signaling pathway: IRS1 |  |  |  |  |  | Y |  |
| Adipocytokine signaling pathway: AGRP |  |  |  |  |  |  | Y |
| Adipocytokine signaling pathway: NPY |  |  | Y | Y |  |  |  |
| Adipocytokine signaling pathway: POMC |  |  | Y | Y |  |  |  |
| Adipocytokine signaling pathway: PPARGC1A |  |  |  |  |  | Y |  |
| Adipocytokine signaling pathway: NFKBIA | Y |  |  |  |  |  |  |
| Vibrio cholerae infection: H+ |  | Y | Y |  |  |  |  |
| Epithelial cell signaling in Helicobacter pylori infection: CXCL8 |  | Y |  |  |  |  |  |
| Epithelial cell signaling in Helicobacter pylori infection: CXCL1 |  | Y |  |  |  |  |  |
| Epithelial cell signaling in Helicobacter pylori infection: H+ |  | Y | Y |  |  |  |  |
| Epithelial cell signaling in Helicobacter pylori infection: CCL5 |  | Y |  |  |  |  |  |
| Pertussis: FOS |  |  |  |  |  | Y |  |
| Pertussis: IRF3 | Y |  | Y |  |  | Y |  |
| Pertussis: C3 |  | Y | Y |  | Y |  |  |
| Pertussis: C5 |  | Y | Y | Y |  |  |  |
| Tuberculosis: IL10 |  | Y | Y | Y |  |  |  |
| Tuberculosis: TGFB1 |  | Y |  |  |  |  |  |
| Tuberculosis: HLA-DMA |  |  | Y |  |  |  |  |
| Tuberculosis: CD74 |  |  | Y |  |  |  |  |
| Tuberculosis: NOS2 |  |  |  |  |  | Y |  |
| Tuberculosis: TNF |  | Y | Y | Y |  |  |  |
| Tuberculosis: IL6 |  | Y | Y | Y |  |  |  |
| Tuberculosis: IL12A |  |  | Y | Y |  | Y |  |
| Tuberculosis: IL18 |  | Y | Y |  |  | Y |  |
| Tuberculosis: IL23A |  | Y | Y | Y |  |  |  |
| Tuberculosis: IL1A |  | Y |  |  |  |  |  |
| Tuberculosis: CAMP |  |  | Y | Y |  |  |  |
| Tuberculosis: CASP3 |  |  |  |  |  |  |  |
| Hepatitis C: IRF3 | Y |  | Y | Y |  | Y |  |
| Hepatitis C: IKBKG CHUK IKBKB | Y |  |  |  |  | Y |  |
| Hepatitis C: STAT3 | Y |  |  |  |  | Y |  |
| Hepatitis C: EIF3E |  |  |  |  |  | Y |  |
| Hepatitis C: GSK3B |  |  |  |  |  | Y | Y |
| Hepatitis B: STAT1 | Y |  |  | Y |  | Y |  |
| Hepatitis B: JUN |  |  |  |  |  | Y |  |
| Hepatitis B: STAT3 | Y |  |  |  |  | Y |  |
| Hepatitis B: CASP3 |  |  |  |  |  |  |  |
| Hepatitis B: STAT3 | Y |  |  |  |  | Y |  |
| Hepatitis B: NFKBIA | Y |  |  |  |  |  |  |
| Hepatitis B: MMP9 |  |  |  |  |  |  |  |
| Hepatitis B: BCL2 |  |  |  |  |  |  |  |
| Hepatitis B: CDKN1A |  |  |  |  |  | Y |  |
| Hepatitis B: MYC |  |  |  |  |  | Y |  |
| Hepatitis B: IFNB1 |  |  |  | Y |  |  |  |
| Measles: IRF3 |  |  |  | Y |  | Y |  |
| Measles: GSK3B |  |  |  |  |  | Y | Y |
| Measles: STAT3 | Y |  |  |  |  | Y |  |
| Influenza A: NFKB1 | Y |  |  |  |  | Y |  |
| Influenza A: IRF3 | Y |  | Y | Y |  | Y |  |
| Influenza A: ATF2 |  |  |  | Y |  | Y |  |
| Influenza A: GSK3B |  |  |  |  |  | Y | Y |
| Influenza A: DNAJC3 HSPA1A | Y |  |  | Y |  | Y |  |
| HTLV-I infection: SLC2A1 |  |  |  |  |  |  | Y |
| HTLV-I infection: CHUK IKBKG | Y |  |  |  |  | Y |  |
| HTLV-I infection: NFKB1 | Y |  |  |  |  | Y |  |
| HTLV-I infection: DLG1 CTNNB1 APC2 | Y |  |  |  |  | Y |  |
| HTLV-I infection: JUN |  |  |  |  |  | Y |  |
| HTLV-I infection: CREBBP TP53 | Y |  |  |  |  | Y |  |
| HTLV-I infection: TP53 TBPL2 | Y |  |  |  |  | Y |  |
| HTLV-I infection: STAT5A |  |  |  |  |  | Y |  |
| Herpes simplex infection: FOS |  |  |  |  |  | Y |  |
| Herpes simplex infection: IKBKG | Y |  |  |  |  | Y |  |
| Herpes simplex infection: IRF3 | Y |  | Y | Y |  | Y |  |
| Herpes simplex infection: NFKB1 | Y |  |  |  |  | Y |  |
| Herpes simplex infection: CHUK |  | Y | Y | Y |  |  |  |
| Herpes simplex infection: EIF2S1 |  |  |  |  |  | Y |  |
| Epstein-Barr virus infection: RB1 | Y |  |  |  |  | Y |  |
| Epstein-Barr virus infection: NFKB1 | Y |  |  |  |  | Y |  |
| Epstein-Barr virus infection: GSK3B |  |  |  |  |  | Y | Y |
| Epstein-Barr virus infection: ATF2 |  |  |  | Y |  | Y |  |
| Epstein-Barr virus infection: NFKB1 | Y |  |  |  |  | Y |  |
| Epstein-Barr virus infection: IRF3 | Y |  | Y | Y |  | Y |  |
| Epstein-Barr virus infection: STAT3 | Y |  |  |  |  | Y |  |

Table S2.

List of drugs targeting highly relevant KDTs.

| **DrugBank ID** | **Drug name** | **Target** | **Total circuits** | **Host-virus interaction** | **Inflammatory response** | **Immune activity** | **Antiviral defense** | **Endocytosis** | **Replication** | **Energetics** |
| --- | --- | --- | --- | --- | --- | --- | --- | --- | --- | --- |
|  |  |  | **All = 277** | **61** | **35** | **32** | **48** | **16** | **137** | **36** |
| [DB00570](https://www.drugbank.ca/drugs/DB00570) | Vinblastine | JUN | 277 | 61 | 35 | 32 | 48 | 16 | 137 | 36 |
| [DB01029](https://www.drugbank.ca/drugs/DB01029) | Irbesartan | JUN | 277 | 61 | 35 | 32 | 48 | 16 | 137 | 36 |
| [DB01169](https://www.drugbank.ca/drugs/DB01169) | Arsenic trioxide | JUN | 277 | 61 | 35 | 32 | 48 | 16 | 137 | 36 |
| [DB01593](https://www.drugbank.ca/drugs/DB01593) | Zinc | INS | 277 | 61 | 35 | 32 | 48 | 16 | 137 | 36 |
| [DB12010](https://www.drugbank.ca/drugs/DB12010) | Fostamatinib | RPS6KA1 | 277 | 61 | 35 | 32 | 48 | 16 | 137 | 36 |
| [DB14487](https://www.drugbank.ca/drugs/DB14487) | Zinc acetate | INS | 277 | 61 | 35 | 32 | 48 | 16 | 137 | 36 |
| [DB14533](https://www.drugbank.ca/drugs/DB14533) | Zinc chloride | INS | 277 | 61 | 35 | 32 | 48 | 16 | 137 | 36 |
| [DB01296](https://www.drugbank.ca/drugs/DB01296) | Glucosamine | NFKB2 | 276 | 61 | 35 | 32 | 48 | 16 | 137 | 36 |
| [DB13751](https://www.drugbank.ca/drugs/DB13751) | Glycyrrhizic acid | NFKB2 | 276 | 61 | 35 | 32 | 48 | 16 | 137 | 36 |
| [DB13961](https://www.drugbank.ca/drugs/DB13961) | Fish oil | NFKB2 | 276 | 61 | 35 | 32 | 48 | 16 | 137 | 36 |
| [DB00381](https://www.drugbank.ca/drugs/DB00381) | Amlodipine | CACNA1B | 275 | 61 | 35 | 31 | 48 | 15 | 137 | 36 |
| [DB00661](https://www.drugbank.ca/drugs/DB00661) | Verapamil | CACNA1B | 275 | 61 | 35 | 31 | 48 | 15 | 137 | 36 |
| [DB00996](https://www.drugbank.ca/drugs/DB00996) | Gabapentin | CACNA1B | 275 | 61 | 35 | 31 | 48 | 15 | 137 | 36 |
| [DB01202](https://www.drugbank.ca/drugs/DB01202) | Levetiracetam | CACNA1B | 275 | 61 | 35 | 31 | 48 | 15 | 137 | 36 |
| [DB00005](https://www.drugbank.ca/drugs/DB00005) | Etanercept | TNFRSF1B | 271 | 59 | 35 | 32 | 47 | 16 | 133 | 34 |
| [DB11626](https://www.drugbank.ca/drugs/DB11626) | Tasonermin | TNFRSF1B | 271 | 59 | 35 | 32 | 47 | 16 | 133 | 34 |
| [DB00201](https://www.drugbank.ca/drugs/DB00201) | Caffeine | RYR1 | 270 | 59 | 35 | 32 | 46 | 16 | 132 | 34 |
| [DB00945](https://www.drugbank.ca/drugs/DB00945) | Acetylsalicylic acid | MYC | 270 | 59 | 35 | 30 | 47 | 16 | 132 | 35 |
| [DB01219](https://www.drugbank.ca/drugs/DB01219) | Dantrolene | RYR1 | 270 | 59 | 35 | 32 | 46 | 16 | 132 | 34 |
| [DB08813](https://www.drugbank.ca/drugs/DB08813) | Nadroparin | MYC | 270 | 59 | 35 | 30 | 47 | 16 | 132 | 35 |
| [DB09085](https://www.drugbank.ca/drugs/DB09085) | Tetracaine | RYR1 | 270 | 59 | 35 | 32 | 46 | 16 | 132 | 34 |
| [DB00367](https://www.drugbank.ca/drugs/DB00367) | Levonorgestrel | SRD5A1 | 269 | 58 | 34 | 32 | 47 | 13 | 134 | 35 |
| [DB01126](https://www.drugbank.ca/drugs/DB01126) | Dutasteride | SRD5A1 | 269 | 58 | 34 | 32 | 47 | 13 | 134 | 35 |
| [DB01169](https://www.drugbank.ca/drugs/DB01169) | Arsenic trioxide | CDKN1A | 269 | 61 | 34 | 32 | 46 | 16 | 136 | 35 |
| [DB01216](https://www.drugbank.ca/drugs/DB01216) | Finasteride | SRD5A1 | 269 | 58 | 34 | 32 | 47 | 13 | 134 | 35 |
| [DB09389](https://www.drugbank.ca/drugs/DB09389) | Norgestrel | SRD5A1 | 269 | 58 | 34 | 32 | 47 | 13 | 134 | 35 |
| [DB00143](https://www.drugbank.ca/drugs/DB00143) | Glutathione | GPX8 | 268 | 59 | 35 | 32 | 47 | 16 | 130 | 33 |
| [DB01404](https://www.drugbank.ca/drugs/DB01404) | Ginseng | IL6 | 268 | 60 | 35 | 30 | 44 | 15 | 136 | 34 |
| [DB09036](https://www.drugbank.ca/drugs/DB09036) | Siltuximab | IL6 | 268 | 60 | 35 | 30 | 44 | 15 | 136 | 34 |
| [DB10770](https://www.drugbank.ca/drugs/DB10770) | Foreskin fibroblast (neonatal) | IL6 | 268 | 60 | 35 | 30 | 44 | 15 | 136 | 34 |
| [DB10772](https://www.drugbank.ca/drugs/DB10772) | Foreskin keratinocyte (neonatal) | IL6 | 268 | 60 | 35 | 30 | 44 | 15 | 136 | 34 |
| [DB11967](https://www.drugbank.ca/drugs/DB11967) | Binimetinib | IL6 | 268 | 60 | 35 | 30 | 44 | 15 | 136 | 34 |
| [DB00005](https://www.drugbank.ca/drugs/DB00005) | Etanercept | TNF | 267 | 59 | 35 | 30 | 46 | 14 | 135 | 33 |
| [DB00051](https://www.drugbank.ca/drugs/DB00051) | Adalimumab | TNF | 267 | 59 | 35 | 30 | 46 | 14 | 135 | 33 |
| [DB00065](https://www.drugbank.ca/drugs/DB00065) | Infliximab | TNF | 267 | 59 | 35 | 30 | 46 | 14 | 135 | 33 |
| [DB00608](https://www.drugbank.ca/drugs/DB00608) | Chloroquine | TNF | 267 | 59 | 35 | 30 | 46 | 14 | 135 | 33 |
| [DB00668](https://www.drugbank.ca/drugs/DB00668) | Epinephrine | TNF | 267 | 59 | 35 | 30 | 46 | 14 | 135 | 33 |
| [DB00852](https://www.drugbank.ca/drugs/DB00852) | Pseudoephedrine | TNF | 267 | 59 | 35 | 30 | 46 | 14 | 135 | 33 |
| [DB01296](https://www.drugbank.ca/drugs/DB01296) | Glucosamine | TNF | 267 | 59 | 35 | 30 | 46 | 14 | 135 | 33 |
| [DB01407](https://www.drugbank.ca/drugs/DB01407) | Clenbuterol | TNF | 267 | 59 | 35 | 30 | 46 | 14 | 135 | 33 |
| [DB01427](https://www.drugbank.ca/drugs/DB01427) | Amrinone | TNF | 267 | 59 | 35 | 30 | 46 | 14 | 135 | 33 |
| [DB02325](https://www.drugbank.ca/drugs/DB02325) | Isopropyl alcohol | TNF | 267 | 59 | 35 | 30 | 46 | 14 | 135 | 33 |
| [DB05676](https://www.drugbank.ca/drugs/DB05676) | Apremilast | TNF | 267 | 59 | 35 | 30 | 46 | 14 | 135 | 33 |
| [DB06674](https://www.drugbank.ca/drugs/DB06674) | Golimumab | TNF | 267 | 59 | 35 | 30 | 46 | 14 | 135 | 33 |
| [DB08904](https://www.drugbank.ca/drugs/DB08904) | Certolizumab pegol | TNF | 267 | 59 | 35 | 30 | 46 | 14 | 135 | 33 |
| [DB08910](https://www.drugbank.ca/drugs/DB08910) | Pomalidomide | TNF | 267 | 59 | 35 | 30 | 46 | 14 | 135 | 33 |
| [DB10770](https://www.drugbank.ca/drugs/DB10770) | Foreskin fibroblast (neonatal) | TNF | 267 | 59 | 35 | 30 | 46 | 14 | 135 | 33 |
| [DB10772](https://www.drugbank.ca/drugs/DB10772) | Foreskin keratinocyte (neonatal) | TNF | 267 | 59 | 35 | 30 | 46 | 14 | 135 | 33 |
| [DB11254](https://www.drugbank.ca/drugs/DB11254) | Hexylresorcinol | TGM2 | 267 | 60 | 33 | 30 | 46 | 16 | 135 | 34 |
| [DB11967](https://www.drugbank.ca/drugs/DB11967) | Binimetinib | TNF | 267 | 59 | 35 | 30 | 46 | 14 | 135 | 33 |
| [DB13751](https://www.drugbank.ca/drugs/DB13751) | Glycyrrhizic acid | TNF | 267 | 59 | 35 | 30 | 46 | 14 | 135 | 33 |
| [DB01254](https://www.drugbank.ca/drugs/DB01254) | Dasatinib | LYN | 266 | 58 | 34 | 30 | 46 | 14 | 130 | 36 |
| [DB06616](https://www.drugbank.ca/drugs/DB06616) | Bosutinib | LYN | 266 | 58 | 34 | 30 | 46 | 14 | 130 | 36 |
| [DB08901](https://www.drugbank.ca/drugs/DB08901) | Ponatinib | LYN | 266 | 58 | 34 | 30 | 46 | 14 | 130 | 36 |
| [DB09079](https://www.drugbank.ca/drugs/DB09079) | Nintedanib | LYN | 266 | 58 | 34 | 30 | 46 | 14 | 130 | 36 |
| [DB12010](https://www.drugbank.ca/drugs/DB12010) | Fostamatinib | LYN | 266 | 58 | 34 | 30 | 46 | 14 | 130 | 36 |
| [DB12010](https://www.drugbank.ca/drugs/DB12010) | Fostamatinib | PIM3 | 266 | 61 | 34 | 31 | 44 | 15 | 135 | 34 |
| [DB00009](https://www.drugbank.ca/drugs/DB00009) | Alteplase | PLAUR | 264 | 58 | 33 | 31 | 45 | 16 | 131 | 33 |
| [DB00015](https://www.drugbank.ca/drugs/DB00015) | Reteplase | PLAUR | 264 | 58 | 33 | 31 | 45 | 16 | 131 | 33 |
| [DB00029](https://www.drugbank.ca/drugs/DB00029) | Anistreplase | PLAUR | 264 | 58 | 33 | 31 | 45 | 16 | 131 | 33 |
| [DB00031](https://www.drugbank.ca/drugs/DB00031) | Tenecteplase | PLAUR | 264 | 58 | 33 | 31 | 45 | 16 | 131 | 33 |
| [DB00641](https://www.drugbank.ca/drugs/DB00641) | Simvastatin | ITGB2 | 264 | 59 | 34 | 31 | 45 | 16 | 131 | 33 |
| [DB00321](https://www.drugbank.ca/drugs/DB00321) | Amitriptyline | NTRK1 | 262 | 59 | 34 | 31 | 47 | 16 | 134 | 31 |
| [DB00619](https://www.drugbank.ca/drugs/DB00619) | Imatinib | NTRK1 | 262 | 59 | 34 | 31 | 47 | 16 | 134 | 31 |
| [DB01092](https://www.drugbank.ca/drugs/DB01092) | Ouabain | ATP1A3 | 262 | 58 | 32 | 30 | 46 | 15 | 132 | 33 |
| [DB08896](https://www.drugbank.ca/drugs/DB08896) | Regorafenib | NTRK1 | 262 | 59 | 34 | 31 | 47 | 16 | 134 | 31 |
| [DB12010](https://www.drugbank.ca/drugs/DB12010) | Fostamatinib | NTRK1 | 262 | 59 | 34 | 31 | 47 | 16 | 134 | 31 |
| [DB13926](https://www.drugbank.ca/drugs/DB13926) | Cenegermin | NTRK1 | 262 | 59 | 34 | 31 | 47 | 16 | 134 | 31 |
| [DB14723](https://www.drugbank.ca/drugs/DB14723) | Larotrectinib | NTRK1 | 262 | 59 | 34 | 31 | 47 | 16 | 134 | 31 |
| [DB00201](https://www.drugbank.ca/drugs/DB00201) | Caffeine | PIK3CB | 261 | 60 | 32 | 30 | 44 | 14 | 135 | 33 |
| [DB00071](https://www.drugbank.ca/drugs/DB00071) | Insulin Pork | IGFBP7 | 259 | 56 | 32 | 31 | 45 | 16 | 126 | 33 |
| [DB03017](https://www.drugbank.ca/drugs/DB03017) | Lauric acid | HNF4A | 258 | 56 | 31 | 30 | 44 | 15 | 127 | 32 |
| [DB08889](https://www.drugbank.ca/drugs/DB08889) | Carfilzomib | PSMB9 | 256 | 59 | 28 | 28 | 41 | 14 | 135 | 32 |
| [DB00136](https://www.drugbank.ca/drugs/DB00136) | Calcitriol | HOXA10 | 255 | 54 | 34 | 30 | 47 | 16 | 125 | 33 |
| [DB08912](https://www.drugbank.ca/drugs/DB08912) | Dabrafenib | SIK1 | 255 | 59 | 29 | 30 | 43 | 16 | 128 | 32 |
| [DB12010](https://www.drugbank.ca/drugs/DB12010) | Fostamatinib | SIK1 | 255 | 59 | 29 | 30 | 43 | 16 | 128 | 32 |
| [DB09061](https://www.drugbank.ca/drugs/DB09061) | Cannabidiol | TRPV4 | 249 | 58 | 30 | 28 | 41 | 14 | 132 | 31 |
| [DB01032](https://www.drugbank.ca/drugs/DB01032) | Probenecid | PANX1 | 248 | 58 | 35 | 30 | 46 | 13 | 123 | 30 |
| [DB06616](https://www.drugbank.ca/drugs/DB06616) | Bosutinib | CDK2 | 248 | 57 | 34 | 31 | 45 | 15 | 118 | 32 |
| [DB00973](https://www.drugbank.ca/drugs/DB00973) | Ezetimibe | SOAT1 | 247 | 57 | 31 | 28 | 43 | 14 | 124 | 32 |
| [DB08813](https://www.drugbank.ca/drugs/DB08813) | Nadroparin | FOS | 247 | 51 | 32 | 28 | 44 | 15 | 124 | 35 |
| [DB00005](https://www.drugbank.ca/drugs/DB00005) | Etanercept | LTA | 246 | 57 | 31 | 27 | 41 | 13 | 127 | 32 |
| [DB00555](https://www.drugbank.ca/drugs/DB00555) | Lamotrigine | SCN2A | 246 | 58 | 35 | 30 | 44 | 16 | 122 | 30 |
| [DB00818](https://www.drugbank.ca/drugs/DB00818) | Propofol | SCN2A | 246 | 58 | 35 | 30 | 44 | 16 | 122 | 30 |
| [DB00909](https://www.drugbank.ca/drugs/DB00909) | Zonisamide | SCN2A | 246 | 58 | 35 | 30 | 44 | 16 | 122 | 30 |
| [DB01050](https://www.drugbank.ca/drugs/DB01050) | Ibuprofen | BCL2 | 245 | 58 | 27 | 29 | 41 | 14 | 133 | 30 |
| [DB01229](https://www.drugbank.ca/drugs/DB01229) | Paclitaxel | BCL2 | 245 | 58 | 27 | 29 | 41 | 14 | 133 | 30 |
| [DB01248](https://www.drugbank.ca/drugs/DB01248) | Docetaxel | BCL2 | 245 | 58 | 27 | 29 | 41 | 14 | 133 | 30 |
| [DB01367](https://www.drugbank.ca/drugs/DB01367) | Rasagiline | BCL2 | 245 | 58 | 27 | 29 | 41 | 14 | 133 | 30 |
| [DB06756](https://www.drugbank.ca/drugs/DB06756) | Glycine betaine | BCL2 | 245 | 58 | 27 | 29 | 41 | 14 | 133 | 30 |
| [DB08871](https://www.drugbank.ca/drugs/DB08871) | Eribulin | BCL2 | 245 | 58 | 27 | 29 | 41 | 14 | 133 | 30 |
| [DB09213](https://www.drugbank.ca/drugs/DB09213) | Dexibuprofen | BCL2 | 245 | 58 | 27 | 29 | 41 | 14 | 133 | 30 |
| [DB09401](https://www.drugbank.ca/drugs/DB09401) | Isosorbide | BCL2 | 245 | 58 | 27 | 29 | 41 | 14 | 133 | 30 |
| [DB11581](https://www.drugbank.ca/drugs/DB11581) | Venetoclax | BCL2 | 245 | 58 | 27 | 29 | 41 | 14 | 133 | 30 |
| [DB06439](https://www.drugbank.ca/drugs/DB06439) | Tyloxapol | LPL | 244 | 51 | 32 | 29 | 44 | 15 | 117 | 29 |
| [DB09568](https://www.drugbank.ca/drugs/DB09568) | Omega-3-carboxylic acids | LPL | 244 | 51 | 32 | 29 | 44 | 15 | 117 | 29 |
| [DB13751](https://www.drugbank.ca/drugs/DB13751) | Glycyrrhizic acid | LPL | 244 | 51 | 32 | 29 | 44 | 15 | 117 | 29 |
| [DB11338](https://www.drugbank.ca/drugs/DB11338) | Clove oil | CXCL10 | 241 | 54 | 32 | 29 | 42 | 14 | 123 | 32 |
| [DB12010](https://www.drugbank.ca/drugs/DB12010) | Fostamatinib | CDC42BPG | 239 | 52 | 33 | 31 | 43 | 16 | 114 | 32 |
| [DB12010](https://www.drugbank.ca/drugs/DB12010) | Fostamatinib | SLK | 238 | 56 | 24 | 26 | 38 | 13 | 132 | 30 |
| [DB00132](https://www.drugbank.ca/drugs/DB00132) | Alpha-Linolenic Acid | PPARG | 237 | 53 | 28 | 24 | 40 | 12 | 119 | 32 |
| [DB00159](https://www.drugbank.ca/drugs/DB00159) | Icosapent | PPARG | 237 | 53 | 28 | 24 | 40 | 12 | 119 | 32 |
| [DB00244](https://www.drugbank.ca/drugs/DB00244) | Mesalazine | PPARG | 237 | 53 | 28 | 24 | 40 | 12 | 119 | 32 |
| [DB00313](https://www.drugbank.ca/drugs/DB00313) | Valproic Acid | PPARG | 237 | 53 | 28 | 24 | 40 | 12 | 119 | 32 |
| [DB00328](https://www.drugbank.ca/drugs/DB00328) | Indometacin | PPARG | 237 | 53 | 28 | 24 | 40 | 12 | 119 | 32 |
| [DB00412](https://www.drugbank.ca/drugs/DB00412) | Rosiglitazone | PPARG | 237 | 53 | 28 | 24 | 40 | 12 | 119 | 32 |
| [DB00573](https://www.drugbank.ca/drugs/DB00573) | Fenoprofen | PPARG | 237 | 53 | 28 | 24 | 40 | 12 | 119 | 32 |
| [DB00731](https://www.drugbank.ca/drugs/DB00731) | Nateglinide | PPARG | 237 | 53 | 28 | 24 | 40 | 12 | 119 | 32 |
| [DB00795](https://www.drugbank.ca/drugs/DB00795) | Sulfasalazine | PPARG | 237 | 53 | 28 | 24 | 40 | 12 | 119 | 32 |
| [DB00912](https://www.drugbank.ca/drugs/DB00912) | Repaglinide | PPARG | 237 | 53 | 28 | 24 | 40 | 12 | 119 | 32 |
| [DB00966](https://www.drugbank.ca/drugs/DB00966) | Telmisartan | PPARG | 237 | 53 | 28 | 24 | 40 | 12 | 119 | 32 |
| [DB01014](https://www.drugbank.ca/drugs/DB01014) | Balsalazide | PPARG | 237 | 53 | 28 | 24 | 40 | 12 | 119 | 32 |
| [DB01039](https://www.drugbank.ca/drugs/DB01039) | Fenofibrate | PPARG | 237 | 53 | 28 | 24 | 40 | 12 | 119 | 32 |
| [DB01050](https://www.drugbank.ca/drugs/DB01050) | Ibuprofen | PPARG | 237 | 53 | 28 | 24 | 40 | 12 | 119 | 32 |
| [DB01067](https://www.drugbank.ca/drugs/DB01067) | Glipizide | PPARG | 237 | 53 | 28 | 24 | 40 | 12 | 119 | 32 |
| [DB01118](https://www.drugbank.ca/drugs/DB01118) | Amiodarone | PPARG | 237 | 53 | 28 | 24 | 40 | 12 | 119 | 32 |
| [DB01132](https://www.drugbank.ca/drugs/DB01132) | Pioglitazone | PPARG | 237 | 53 | 28 | 24 | 40 | 12 | 119 | 32 |
| [DB01393](https://www.drugbank.ca/drugs/DB01393) | Bezafibrate | PPARG | 237 | 53 | 28 | 24 | 40 | 12 | 119 | 32 |
| [DB02266](https://www.drugbank.ca/drugs/DB02266) | Flufenamic Acid | PPARG | 237 | 53 | 28 | 24 | 40 | 12 | 119 | 32 |
| [DB02709](https://www.drugbank.ca/drugs/DB02709) | Resveratrol | PPARG | 237 | 53 | 28 | 24 | 40 | 12 | 119 | 32 |
| [DB03756](https://www.drugbank.ca/drugs/DB03756) | Doconexent | PPARG | 237 | 53 | 28 | 24 | 40 | 12 | 119 | 32 |
| [DB04224](https://www.drugbank.ca/drugs/DB04224) | Oleic Acid | PPARG | 237 | 53 | 28 | 24 | 40 | 12 | 119 | 32 |
| [DB06589](https://www.drugbank.ca/drugs/DB06589) | Pazopanib | SH2B3 | 237 | 58 | 31 | 28 | 40 | 16 | 118 | 31 |
| [DB08604](https://www.drugbank.ca/drugs/DB08604) | Triclosan | PPARG | 237 | 53 | 28 | 24 | 40 | 12 | 119 | 32 |
| [DB09061](https://www.drugbank.ca/drugs/DB09061) | Cannabidiol | PPARG | 237 | 53 | 28 | 24 | 40 | 12 | 119 | 32 |
| [DB09213](https://www.drugbank.ca/drugs/DB09213) | Dexibuprofen | PPARG | 237 | 53 | 28 | 24 | 40 | 12 | 119 | 32 |
| [DB11133](https://www.drugbank.ca/drugs/DB11133) | Omega-3 fatty acids | PPARG | 237 | 53 | 28 | 24 | 40 | 12 | 119 | 32 |
| [DB11672](https://www.drugbank.ca/drugs/DB11672) | Curcumin | PPARG | 237 | 53 | 28 | 24 | 40 | 12 | 119 | 32 |
| [DB13873](https://www.drugbank.ca/drugs/DB13873) | Fenofibric acid | PPARG | 237 | 53 | 28 | 24 | 40 | 12 | 119 | 32 |
| [DB13961](https://www.drugbank.ca/drugs/DB13961) | Fish oil | PPARG | 237 | 53 | 28 | 24 | 40 | 12 | 119 | 32 |
| [DB00182](https://www.drugbank.ca/drugs/DB00182) | Amphetamine | MAOA | 235 | 54 | 30 | 30 | 41 | 13 | 116 | 28 |
| [DB00191](https://www.drugbank.ca/drugs/DB00191) | Phentermine | MAOA | 235 | 54 | 30 | 30 | 41 | 13 | 116 | 28 |
| [DB00721](https://www.drugbank.ca/drugs/DB00721) | Procaine | MAOA | 235 | 54 | 30 | 30 | 41 | 13 | 116 | 28 |
| [DB00752](https://www.drugbank.ca/drugs/DB00752) | Tranylcypromine | MAOA | 235 | 54 | 30 | 30 | 41 | 13 | 116 | 28 |
| [DB00780](https://www.drugbank.ca/drugs/DB00780) | Phenelzine | MAOA | 235 | 54 | 30 | 30 | 41 | 13 | 116 | 28 |
| [DB00805](https://www.drugbank.ca/drugs/DB00805) | Minaprine | MAOA | 235 | 54 | 30 | 30 | 41 | 13 | 116 | 28 |
| [DB00909](https://www.drugbank.ca/drugs/DB00909) | Zonisamide | MAOA | 235 | 54 | 30 | 30 | 41 | 13 | 116 | 28 |
| [DB01037](https://www.drugbank.ca/drugs/DB01037) | Selegiline | MAOA | 235 | 54 | 30 | 30 | 41 | 13 | 116 | 28 |
| [DB01168](https://www.drugbank.ca/drugs/DB01168) | Procarbazine | MAOA | 235 | 54 | 30 | 30 | 41 | 13 | 116 | 28 |
| [DB01171](https://www.drugbank.ca/drugs/DB01171) | Moclobemide | MAOA | 235 | 54 | 30 | 30 | 41 | 13 | 116 | 28 |
| [DB01171](https://www.drugbank.ca/drugs/DB01171) | Moclobemide | MAOA | 235 | 54 | 30 | 30 | 41 | 13 | 116 | 28 |
| [DB01247](https://www.drugbank.ca/drugs/DB01247) | Isocarboxazid | MAOA | 235 | 54 | 30 | 30 | 41 | 13 | 116 | 28 |
| [DB01577](https://www.drugbank.ca/drugs/DB01577) | Metamfetamine | MAOA | 235 | 54 | 30 | 30 | 41 | 13 | 116 | 28 |
| [DB01626](https://www.drugbank.ca/drugs/DB01626) | Pargyline | MAOA | 235 | 54 | 30 | 30 | 41 | 13 | 116 | 28 |
| [DB03147](https://www.drugbank.ca/drugs/DB03147) | Flavin adenine dinucleotide | MAOA | 235 | 54 | 30 | 30 | 41 | 13 | 116 | 28 |
| [DB00142](https://www.drugbank.ca/drugs/DB00142) | Glutamic Acid | SLC1A6 | 234 | 49 | 33 | 28 | 42 | 14 | 116 | 33 |
| [DB00170](https://www.drugbank.ca/drugs/DB00170) | Menadione | VKORC1L1 | 234 | 54 | 21 | 25 | 36 | 13 | 128 | 30 |
| [DB00720](https://www.drugbank.ca/drugs/DB00720) | Clodronic acid | SLC25A4 | 230 | 51 | 29 | 28 | 36 | 12 | 115 | 28 |
| [DB01593](https://www.drugbank.ca/drugs/DB01593) | Zinc | SERPINA1 | 227 | 52 | 31 | 29 | 45 | 16 | 110 | 24 |
| [DB09130](https://www.drugbank.ca/drugs/DB09130) | Copper | SERPINA1 | 227 | 52 | 31 | 29 | 45 | 16 | 110 | 24 |
| [DB12010](https://www.drugbank.ca/drugs/DB12010) | Fostamatinib | AURKA | 227 | 52 | 27 | 27 | 40 | 16 | 114 | 31 |
| [DB14487](https://www.drugbank.ca/drugs/DB14487) | Zinc acetate | SERPINA1 | 227 | 52 | 31 | 29 | 45 | 16 | 110 | 24 |
| [DB14533](https://www.drugbank.ca/drugs/DB14533) | Zinc chloride | SERPINA1 | 227 | 52 | 31 | 29 | 45 | 16 | 110 | 24 |
| [DB00095](https://www.drugbank.ca/drugs/DB00095) | Efalizumab | ITGAL | 224 | 54 | 16 | 25 | 32 | 13 | 126 | 31 |
| [DB00098](https://www.drugbank.ca/drugs/DB00098) | Antithymocyte immunoglobulin (rabbit) | ITGAL | 224 | 54 | 16 | 25 | 32 | 13 | 126 | 31 |
| [DB00227](https://www.drugbank.ca/drugs/DB00227) | Lovastatin | ITGAL | 224 | 54 | 16 | 25 | 32 | 13 | 126 | 31 |
| [DB00641](https://www.drugbank.ca/drugs/DB00641) | Simvastatin | ITGAL | 224 | 54 | 16 | 25 | 32 | 13 | 126 | 31 |
| [DB11611](https://www.drugbank.ca/drugs/DB11611) | Lifitegrast | ITGAL | 224 | 54 | 16 | 25 | 32 | 13 | 126 | 31 |
| [DB03796](https://www.drugbank.ca/drugs/DB03796) | Palmitic Acid | PMP2 | 223 | 46 | 32 | 24 | 40 | 13 | 102 | 34 |
| [DB04224](https://www.drugbank.ca/drugs/DB04224) | Oleic Acid | PMP2 | 223 | 46 | 32 | 24 | 40 | 13 | 102 | 34 |
| [DB09033](https://www.drugbank.ca/drugs/DB09033) | Vedolizumab | ITGB7 | 217 | 50 | 29 | 22 | 37 | 13 | 109 | 28 |
| [DB00108](https://www.drugbank.ca/drugs/DB00108) | Natalizumab | ITGA4 | 216 | 54 | 24 | 25 | 38 | 12 | 109 | 28 |
| [DB06822](https://www.drugbank.ca/drugs/DB06822) | Tinzaparin | ITGA4 | 216 | 54 | 24 | 25 | 38 | 12 | 109 | 28 |
| [DB09033](https://www.drugbank.ca/drugs/DB09033) | Vedolizumab | ITGA4 | 216 | 54 | 24 | 25 | 38 | 12 | 109 | 28 |
| [DB00072](https://www.drugbank.ca/drugs/DB00072) | Trastuzumab | ERBB2 | 213 | 42 | 22 | 27 | 35 | 12 | 109 | 23 |
| [DB01259](https://www.drugbank.ca/drugs/DB01259) | Lapatinib | ERBB2 | 213 | 42 | 22 | 27 | 35 | 12 | 109 | 23 |
| [DB05773](https://www.drugbank.ca/drugs/DB05773) | Trastuzumab emtansine | ERBB2 | 213 | 42 | 22 | 27 | 35 | 12 | 109 | 23 |
| [DB06366](https://www.drugbank.ca/drugs/DB06366) | Pertuzumab | ERBB2 | 213 | 42 | 22 | 27 | 35 | 12 | 109 | 23 |
| [DB08916](https://www.drugbank.ca/drugs/DB08916) | Afatinib | ERBB2 | 213 | 42 | 22 | 27 | 35 | 12 | 109 | 23 |
| [DB12010](https://www.drugbank.ca/drugs/DB12010) | Fostamatinib | ERBB2 | 213 | 42 | 22 | 27 | 35 | 12 | 109 | 23 |
| [DB12267](https://www.drugbank.ca/drugs/DB12267) | Brigatinib | ERBB2 | 213 | 42 | 22 | 27 | 35 | 12 | 109 | 23 |
| [DB12010](https://www.drugbank.ca/drugs/DB12010) | Fostamatinib | PAK6 | 211 | 44 | 28 | 28 | 41 | 11 | 108 | 26 |
| [DB00114](https://www.drugbank.ca/drugs/DB00114) | Pyridoxal phosphate | CTH | 207 | 49 | 19 | 22 | 33 | 10 | 118 | 30 |
| [DB00151](https://www.drugbank.ca/drugs/DB00151) | L-Cysteine | CTH | 207 | 49 | 19 | 22 | 33 | 10 | 118 | 30 |
| [DB12010](https://www.drugbank.ca/drugs/DB12010) | Fostamatinib | AAK1 | 207 | 54 | 25 | 26 | 38 | 12 | 106 | 26 |
| [DB08814](https://www.drugbank.ca/drugs/DB08814) | Triflusal | NFKB1 | 206 | 51 | 29 | 26 | 38 | 14 | 101 | 25 |
| [DB00121](https://www.drugbank.ca/drugs/DB00121) | Biotin | PCCB | 198 | 49 | 23 | 21 | 33 | 14 | 101 | 28 |
| [DB00161](https://www.drugbank.ca/drugs/DB00161) | L-Valine | PCCB | 198 | 49 | 23 | 21 | 33 | 14 | 101 | 28 |
| [DB00163](https://www.drugbank.ca/drugs/DB00163) | Vitamin E | SEC14L2 | 198 | 47 | 10 | 21 | 27 | 13 | 117 | 31 |
| [DB03796](https://www.drugbank.ca/drugs/DB03796) | Palmitic Acid | SEC14L2 | 198 | 47 | 10 | 21 | 27 | 13 | 117 | 31 |
| [DB14001](https://www.drugbank.ca/drugs/DB14001) | alpha-Tocopherol succinate | SEC14L2 | 198 | 47 | 10 | 21 | 27 | 13 | 117 | 31 |
| [DB14002](https://www.drugbank.ca/drugs/DB14002) | D-alpha-Tocopherol acetate | SEC14L2 | 198 | 47 | 10 | 21 | 27 | 13 | 117 | 31 |
| [DB05389](https://www.drugbank.ca/drugs/DB05389) | Tetrachlorodecaoxide | CD163 | 197 | 43 | 29 | 24 | 39 | 10 | 96 | 21 |
| [DB00108](https://www.drugbank.ca/drugs/DB00108) | Natalizumab | ICAM1 | 193 | 38 | 34 | 28 | 39 | 10 | 94 | 17 |
| [DB08818](https://www.drugbank.ca/drugs/DB08818) | Hyaluronic acid | ICAM1 | 193 | 38 | 34 | 28 | 39 | 10 | 94 | 17 |
| [DB00024](https://www.drugbank.ca/drugs/DB00024) | Thyrotropin alfa | TSHR | 191 | 42 | 26 | 21 | 35 | 14 | 91 | 14 |
| [DB01045](https://www.drugbank.ca/drugs/DB01045) | Rifampicin | SLCO1A2 | 190 | 39 | 28 | 21 | 34 | 10 | 88 | 30 |
| [DB00364](https://www.drugbank.ca/drugs/DB00364) | Sucralfate | FGF2 | 189 | 45 | 22 | 24 | 30 | 10 | 102 | 25 |
| [DB00389](https://www.drugbank.ca/drugs/DB00389) | Carbimazole | TPO | 189 | 40 | 24 | 21 | 35 | 13 | 90 | 14 |
| [DB00509](https://www.drugbank.ca/drugs/DB00509) | Dextrothyroxine | TPO | 189 | 40 | 24 | 21 | 35 | 13 | 90 | 14 |
| [DB00550](https://www.drugbank.ca/drugs/DB00550) | Propylthiouracil | TPO | 189 | 40 | 24 | 21 | 35 | 13 | 90 | 14 |
| [DB00686](https://www.drugbank.ca/drugs/DB00686) | Pentosan Polysulfate | FGF2 | 189 | 45 | 22 | 24 | 30 | 10 | 102 | 25 |
| [DB00763](https://www.drugbank.ca/drugs/DB00763) | Methimazole | TPO | 189 | 40 | 24 | 21 | 35 | 13 | 90 | 14 |
| [DB00877](https://www.drugbank.ca/drugs/DB00877) | Sirolimus | FGF2 | 189 | 45 | 22 | 24 | 30 | 10 | 102 | 25 |
| [DB01109](https://www.drugbank.ca/drugs/DB01109) | Heparin | FGF2 | 189 | 45 | 22 | 24 | 30 | 10 | 102 | 25 |
| [DB08604](https://www.drugbank.ca/drugs/DB08604) | Triclosan | TPO | 189 | 40 | 24 | 21 | 35 | 13 | 90 | 14 |
| [DB11085](https://www.drugbank.ca/drugs/DB11085) | Resorcinol | TPO | 189 | 40 | 24 | 21 | 35 | 13 | 90 | 14 |
| [DB11496](https://www.drugbank.ca/drugs/DB11496) | 2-mercaptobenzothiazole | TPO | 189 | 40 | 24 | 21 | 35 | 13 | 90 | 14 |
| [DB00768](https://www.drugbank.ca/drugs/DB00768) | Olopatadine | S100B | 187 | 35 | 29 | 18 | 30 | 9 | 90 | 23 |
| [DB01373](https://www.drugbank.ca/drugs/DB01373) | Calcium | S100B | 187 | 35 | 29 | 18 | 30 | 9 | 90 | 23 |
| [DB11093](https://www.drugbank.ca/drugs/DB11093) | Calcium Citrate | S100B | 187 | 35 | 29 | 18 | 30 | 9 | 90 | 23 |
| [DB11348](https://www.drugbank.ca/drugs/DB11348) | Calcium Phosphate | S100B | 187 | 35 | 29 | 18 | 30 | 9 | 90 | 23 |
| [DB12010](https://www.drugbank.ca/drugs/DB12010) | Fostamatinib | TEC | 187 | 42 | 18 | 22 | 28 | 12 | 101 | 29 |
| [DB14481](https://www.drugbank.ca/drugs/DB14481) | Calcium phosphate dihydrate | S100B | 187 | 35 | 29 | 18 | 30 | 9 | 90 | 23 |
| [DB01045](https://www.drugbank.ca/drugs/DB01045) | Rifampicin | SLCO2B1 | 182 | 39 | 33 | 21 | 32 | 7 | 83 | 19 |
| [DB13874](https://www.drugbank.ca/drugs/DB13874) | Enasidenib | IDH2 | 182 | 38 | 27 | 20 | 31 | 10 | 85 | 23 |
| [DB00887](https://www.drugbank.ca/drugs/DB00887) | Bumetanide | CFTR | 179 | 34 | 24 | 22 | 29 | 12 | 78 | 26 |
| [DB01016](https://www.drugbank.ca/drugs/DB01016) | Glyburide | CFTR | 179 | 34 | 24 | 22 | 29 | 12 | 78 | 26 |
| [DB01050](https://www.drugbank.ca/drugs/DB01050) | Ibuprofen | CFTR | 179 | 34 | 24 | 22 | 29 | 12 | 78 | 26 |
| [DB04941](https://www.drugbank.ca/drugs/DB04941) | Crofelemer | CFTR | 179 | 34 | 24 | 22 | 29 | 12 | 78 | 26 |
| [DB08820](https://www.drugbank.ca/drugs/DB08820) | Ivacaftor | CFTR | 179 | 34 | 24 | 22 | 29 | 12 | 78 | 26 |
| [DB09213](https://www.drugbank.ca/drugs/DB09213) | Dexibuprofen | CFTR | 179 | 34 | 24 | 22 | 29 | 12 | 78 | 26 |
| [DB09280](https://www.drugbank.ca/drugs/DB09280) | Lumacaftor | CFTR | 179 | 34 | 24 | 22 | 29 | 12 | 78 | 26 |
| [DB11712](https://www.drugbank.ca/drugs/DB11712) | Tezacaftor | CFTR | 179 | 34 | 24 | 22 | 29 | 12 | 78 | 26 |
| [DB00141](https://www.drugbank.ca/drugs/DB00141) | N-Acetylglucosamine | B4GALT1 | 178 | 41 | 11 | 19 | 25 | 12 | 99 | 31 |
| [DB12159](https://www.drugbank.ca/drugs/DB12159) | Dupilumab | IL4R | 178 | 35 | 22 | 15 | 27 | 11 | 84 | 27 |
| [DB01136](https://www.drugbank.ca/drugs/DB01136) | Carvedilol | HIF1A | 177 | 44 | 13 | 19 | 24 | 11 | 104 | 29 |
| [DB00157](https://www.drugbank.ca/drugs/DB00157) | NADH | ALDH6A1 | 175 | 41 | 16 | 17 | 29 | 10 | 103 | 26 |
| [DB00945](https://www.drugbank.ca/drugs/DB00945) | Acetylsalicylic acid | NFKBIA | 172 | 42 | 27 | 20 | 34 | 10 | 81 | 13 |
| [DB00177](https://www.drugbank.ca/drugs/DB00177) | Valsartan | AGTR1 | 170 | 41 | 6 | 16 | 21 | 10 | 101 | 27 |
| [DB00275](https://www.drugbank.ca/drugs/DB00275) | Olmesartan | AGTR1 | 170 | 41 | 6 | 16 | 21 | 10 | 101 | 27 |
| [DB00678](https://www.drugbank.ca/drugs/DB00678) | Losartan | AGTR1 | 170 | 41 | 6 | 16 | 21 | 10 | 101 | 27 |
| [DB00796](https://www.drugbank.ca/drugs/DB00796) | Candesartan cilexetil | AGTR1 | 170 | 41 | 6 | 16 | 21 | 10 | 101 | 27 |
| [DB00876](https://www.drugbank.ca/drugs/DB00876) | Eprosartan | AGTR1 | 170 | 41 | 6 | 16 | 21 | 10 | 101 | 27 |
| [DB00966](https://www.drugbank.ca/drugs/DB00966) | Telmisartan | AGTR1 | 170 | 41 | 6 | 16 | 21 | 10 | 101 | 27 |
| [DB01029](https://www.drugbank.ca/drugs/DB01029) | Irbesartan | AGTR1 | 170 | 41 | 6 | 16 | 21 | 10 | 101 | 27 |
| [DB08822](https://www.drugbank.ca/drugs/DB08822) | Azilsartan medoxomil | AGTR1 | 170 | 41 | 6 | 16 | 21 | 10 | 101 | 27 |
| [DB11842](https://www.drugbank.ca/drugs/DB11842) | Angiotensin II | AGTR1 | 170 | 41 | 6 | 16 | 21 | 10 | 101 | 27 |
| [DB00075](https://www.drugbank.ca/drugs/DB00075) | Muromonab | CD247 | 169 | 43 | 14 | 19 | 27 | 11 | 101 | 26 |
| [DB00181](https://www.drugbank.ca/drugs/DB00181) | Baclofen | GABBR2 | 168 | 35 | 19 | 18 | 26 | 9 | 84 | 29 |
| [DB00996](https://www.drugbank.ca/drugs/DB00996) | Gabapentin | GABBR2 | 168 | 35 | 19 | 18 | 26 | 9 | 84 | 29 |
| [DB02530](https://www.drugbank.ca/drugs/DB02530) | gamma-Aminobutyric acid | GABBR2 | 168 | 35 | 19 | 18 | 26 | 9 | 84 | 29 |
| [DB01254](https://www.drugbank.ca/drugs/DB01254) | Dasatinib | LCK | 163 | 46 | 20 | 17 | 27 | 9 | 92 | 13 |
| [DB08901](https://www.drugbank.ca/drugs/DB08901) | Ponatinib | LCK | 163 | 46 | 20 | 17 | 27 | 9 | 92 | 13 |
| [DB09079](https://www.drugbank.ca/drugs/DB09079) | Nintedanib | LCK | 163 | 46 | 20 | 17 | 27 | 9 | 92 | 13 |
| [DB12010](https://www.drugbank.ca/drugs/DB12010) | Fostamatinib | LCK | 163 | 46 | 20 | 17 | 27 | 9 | 92 | 13 |
| [DB00157](https://www.drugbank.ca/drugs/DB00157) | NADH | IMPDH1 | 162 | 36 | 17 | 16 | 26 | 9 | 99 | 28 |
| [DB00231](https://www.drugbank.ca/drugs/DB00231) | Temazepam | GABRA2 | 162 | 34 | 18 | 13 | 23 | 10 | 78 | 24 |
| [DB00241](https://www.drugbank.ca/drugs/DB00241) | Butalbital | GABRA2 | 162 | 34 | 18 | 13 | 23 | 10 | 78 | 24 |
| [DB00306](https://www.drugbank.ca/drugs/DB00306) | Talbutal | GABRA2 | 162 | 34 | 18 | 13 | 23 | 10 | 78 | 24 |
| [DB00312](https://www.drugbank.ca/drugs/DB00312) | Pentobarbital | GABRA2 | 162 | 34 | 18 | 13 | 23 | 10 | 78 | 24 |
| [DB00371](https://www.drugbank.ca/drugs/DB00371) | Meprobamate | GABRA2 | 162 | 34 | 18 | 13 | 23 | 10 | 78 | 24 |
| [DB00402](https://www.drugbank.ca/drugs/DB00402) | Eszopiclone | GABRA2 | 162 | 34 | 18 | 13 | 23 | 10 | 78 | 24 |
| [DB00404](https://www.drugbank.ca/drugs/DB00404) | Alprazolam | GABRA2 | 162 | 34 | 18 | 13 | 23 | 10 | 78 | 24 |
| [DB00418](https://www.drugbank.ca/drugs/DB00418) | Secobarbital | GABRA2 | 162 | 34 | 18 | 13 | 23 | 10 | 78 | 24 |
| [DB00425](https://www.drugbank.ca/drugs/DB00425) | Zolpidem | GABRA2 | 162 | 34 | 18 | 13 | 23 | 10 | 78 | 24 |
| [DB00599](https://www.drugbank.ca/drugs/DB00599) | Thiopental | GABRA2 | 162 | 34 | 18 | 13 | 23 | 10 | 78 | 24 |
| [DB00683](https://www.drugbank.ca/drugs/DB00683) | Midazolam | GABRA2 | 162 | 34 | 18 | 13 | 23 | 10 | 78 | 24 |
| [DB00688](https://www.drugbank.ca/drugs/DB00688) | Mycophenolate mofetil | IMPDH1 | 162 | 36 | 17 | 16 | 26 | 9 | 99 | 28 |
| [DB00690](https://www.drugbank.ca/drugs/DB00690) | Flurazepam | GABRA2 | 162 | 34 | 18 | 13 | 23 | 10 | 78 | 24 |
| [DB00794](https://www.drugbank.ca/drugs/DB00794) | Primidone | GABRA2 | 162 | 34 | 18 | 13 | 23 | 10 | 78 | 24 |
| [DB00811](https://www.drugbank.ca/drugs/DB00811) | Ribavirin | IMPDH1 | 162 | 36 | 17 | 16 | 26 | 9 | 99 | 28 |
| [DB00829](https://www.drugbank.ca/drugs/DB00829) | Diazepam | GABRA2 | 162 | 34 | 18 | 13 | 23 | 10 | 78 | 24 |
| [DB00842](https://www.drugbank.ca/drugs/DB00842) | Oxazepam | GABRA2 | 162 | 34 | 18 | 13 | 23 | 10 | 78 | 24 |
| [DB00849](https://www.drugbank.ca/drugs/DB00849) | Methylphenobarbital | GABRA2 | 162 | 34 | 18 | 13 | 23 | 10 | 78 | 24 |
| [DB00897](https://www.drugbank.ca/drugs/DB00897) | Triazolam | GABRA2 | 162 | 34 | 18 | 13 | 23 | 10 | 78 | 24 |
| [DB00898](https://www.drugbank.ca/drugs/DB00898) | Ethanol | GABRA2 | 162 | 34 | 18 | 13 | 23 | 10 | 78 | 24 |
| [DB01024](https://www.drugbank.ca/drugs/DB01024) | Mycophenolic acid | IMPDH1 | 162 | 36 | 17 | 16 | 26 | 9 | 99 | 28 |
| [DB01033](https://www.drugbank.ca/drugs/DB01033) | Mercaptopurine | IMPDH1 | 162 | 36 | 17 | 16 | 26 | 9 | 99 | 28 |
| [DB01198](https://www.drugbank.ca/drugs/DB01198) | Zopiclone | GABRA2 | 162 | 34 | 18 | 13 | 23 | 10 | 78 | 24 |
| [DB01215](https://www.drugbank.ca/drugs/DB01215) | Estazolam | GABRA2 | 162 | 34 | 18 | 13 | 23 | 10 | 78 | 24 |
| [DB01346](https://www.drugbank.ca/drugs/DB01346) | Quinidine barbiturate | GABRA2 | 162 | 34 | 18 | 13 | 23 | 10 | 78 | 24 |
| [DB01351](https://www.drugbank.ca/drugs/DB01351) | Amobarbital | GABRA2 | 162 | 34 | 18 | 13 | 23 | 10 | 78 | 24 |
| [DB01353](https://www.drugbank.ca/drugs/DB01353) | Butobarbital | GABRA2 | 162 | 34 | 18 | 13 | 23 | 10 | 78 | 24 |
| [DB01544](https://www.drugbank.ca/drugs/DB01544) | Flunitrazepam | GABRA2 | 162 | 34 | 18 | 13 | 23 | 10 | 78 | 24 |
| [DB01558](https://www.drugbank.ca/drugs/DB01558) | Bromazepam | GABRA2 | 162 | 34 | 18 | 13 | 23 | 10 | 78 | 24 |
| [DB01559](https://www.drugbank.ca/drugs/DB01559) | Clotiazepam | GABRA2 | 162 | 34 | 18 | 13 | 23 | 10 | 78 | 24 |
| [DB01588](https://www.drugbank.ca/drugs/DB01588) | Prazepam | GABRA2 | 162 | 34 | 18 | 13 | 23 | 10 | 78 | 24 |
| [DB01589](https://www.drugbank.ca/drugs/DB01589) | Quazepam | GABRA2 | 162 | 34 | 18 | 13 | 23 | 10 | 78 | 24 |
| [DB01595](https://www.drugbank.ca/drugs/DB01595) | Nitrazepam | GABRA2 | 162 | 34 | 18 | 13 | 23 | 10 | 78 | 24 |
| [DB08809](https://www.drugbank.ca/drugs/DB08809) | Dichloroacetic acid | PDK1 | 162 | 34 | 21 | 17 | 29 | 9 | 93 | 17 |
| [DB13872](https://www.drugbank.ca/drugs/DB13872) | Lormetazepam | GABRA2 | 162 | 34 | 18 | 13 | 23 | 10 | 78 | 24 |
| [DB00825](https://www.drugbank.ca/drugs/DB00825) | Levomenthol | TRPM8 | 161 | 30 | 24 | 19 | 26 | 10 | 69 | 21 |
| [DB01744](https://www.drugbank.ca/drugs/DB01744) | Camphor | TRPM8 | 161 | 30 | 24 | 19 | 26 | 10 | 69 | 21 |
| [DB09061](https://www.drugbank.ca/drugs/DB09061) | Cannabidiol | TRPM8 | 161 | 30 | 24 | 19 | 26 | 10 | 69 | 21 |
| [DB00117](https://www.drugbank.ca/drugs/DB00117) | Histidine | HARS | 158 | 32 | 8 | 10 | 19 | 10 | 85 | 27 |
| [DB00255](https://www.drugbank.ca/drugs/DB00255) | Diethylstilbestrol | ESRRB | 158 | 31 | 22 | 16 | 22 | 3 | 75 | 20 |
| [DB07776](https://www.drugbank.ca/drugs/DB07776) | Flavone | ESRRB | 158 | 31 | 22 | 16 | 22 | 3 | 75 | 20 |
| [DB00143](https://www.drugbank.ca/drugs/DB00143) | Glutathione | GPX3 | 156 | 39 | 17 | 22 | 23 | 9 | 75 | 21 |
| [DB00061](https://www.drugbank.ca/drugs/DB00061) | Pegademase | GRB2 | 154 | 36 | 14 | 18 | 28 | 8 | 76 | 23 |
| [DB00114](https://www.drugbank.ca/drugs/DB00114) | Pyridoxal phosphate | CCBL1 | 152 | 39 | 11 | 18 | 22 | 9 | 89 | 21 |
| [DB01169](https://www.drugbank.ca/drugs/DB01169) | Arsenic trioxide | PML | 151 | 32 | 21 | 22 | 29 | 8 | 72 | 18 |
| [DB00909](https://www.drugbank.ca/drugs/DB00909) | Zonisamide | SCN4B | 150 | 41 | 19 | 18 | 28 | 13 | 78 | 14 |
| [DB01109](https://www.drugbank.ca/drugs/DB01109) | Heparin | SELP | 149 | 29 | 27 | 15 | 22 | 7 | 70 | 17 |
| [DB06779](https://www.drugbank.ca/drugs/DB06779) | Dalteparin | SELP | 149 | 29 | 27 | 15 | 22 | 7 | 70 | 17 |
| [DB08813](https://www.drugbank.ca/drugs/DB08813) | Nadroparin | SELP | 149 | 29 | 27 | 15 | 22 | 7 | 70 | 17 |
| [DB09130](https://www.drugbank.ca/drugs/DB09130) | Copper | BDNF | 148 | 29 | 9 | 16 | 17 | 7 | 82 | 30 |
| [DB09301](https://www.drugbank.ca/drugs/DB09301) | Chondroitin sulfate | BDNF | 148 | 29 | 9 | 16 | 17 | 7 | 82 | 30 |
| [DB11823](https://www.drugbank.ca/drugs/DB11823) | Esketamine | BDNF | 148 | 29 | 9 | 16 | 17 | 7 | 82 | 30 |
| [DB03147](https://www.drugbank.ca/drugs/DB03147) | Flavin adenine dinucleotide | ACOX1 | 142 | 33 | 17 | 19 | 29 | 7 | 76 | 14 |
| [DB01593](https://www.drugbank.ca/drugs/DB01593) | Zinc | KRT14 | 141 | 34 | 23 | 17 | 28 | 9 | 60 | 13 |
| [DB14487](https://www.drugbank.ca/drugs/DB14487) | Zinc acetate | KRT14 | 141 | 34 | 23 | 17 | 28 | 9 | 60 | 13 |
| [DB14533](https://www.drugbank.ca/drugs/DB14533) | Zinc chloride | KRT14 | 141 | 34 | 23 | 17 | 28 | 9 | 60 | 13 |
| [DB01593](https://www.drugbank.ca/drugs/DB01593) | Zinc | CFB | 139 | 36 | 27 | 23 | 31 | 11 | 66 | 6 |
| [DB14487](https://www.drugbank.ca/drugs/DB14487) | Zinc acetate | CFB | 139 | 36 | 27 | 23 | 31 | 11 | 66 | 6 |
| [DB14533](https://www.drugbank.ca/drugs/DB14533) | Zinc chloride | CFB | 139 | 36 | 27 | 23 | 31 | 11 | 66 | 6 |
| [DB00277](https://www.drugbank.ca/drugs/DB00277) | Theophylline | RIC3 | 138 | 28 | 23 | 15 | 19 | 6 | 67 | 17 |
| [DB00755](https://www.drugbank.ca/drugs/DB00755) | Tretinoin | RBP4 | 138 | 29 | 18 | 17 | 30 | 8 | 65 | 16 |
| [DB00157](https://www.drugbank.ca/drugs/DB00157) | NADH | NDUFS6 | 137 | 32 | 4 | 7 | 15 | 7 | 86 | 22 |
| [DB00348](https://www.drugbank.ca/drugs/DB00348) | Nitisinone | HPD | 137 | 37 | 7 | 12 | 17 | 9 | 83 | 26 |
| [DB00471](https://www.drugbank.ca/drugs/DB00471) | Montelukast | CYSLTR1 | 136 | 27 | 15 | 14 | 24 | 5 | 71 | 23 |
| [DB00549](https://www.drugbank.ca/drugs/DB00549) | Zafirlukast | CYSLTR1 | 136 | 27 | 15 | 14 | 24 | 5 | 71 | 23 |
| [DB00716](https://www.drugbank.ca/drugs/DB00716) | Nedocromil | CYSLTR1 | 136 | 27 | 15 | 14 | 24 | 5 | 71 | 23 |
| [DB00216](https://www.drugbank.ca/drugs/DB00216) | Eletriptan | HTR1B | 131 | 26 | 27 | 15 | 25 | 9 | 53 | 16 |
| [DB00246](https://www.drugbank.ca/drugs/DB00246) | Ziprasidone | HTR1B | 131 | 26 | 27 | 15 | 25 | 9 | 53 | 16 |
| [DB00247](https://www.drugbank.ca/drugs/DB00247) | Methysergide | HTR1B | 131 | 26 | 27 | 15 | 25 | 9 | 53 | 16 |
| [DB00248](https://www.drugbank.ca/drugs/DB00248) | Cabergoline | HTR1B | 131 | 26 | 27 | 15 | 25 | 9 | 53 | 16 |
| [DB00315](https://www.drugbank.ca/drugs/DB00315) | Zolmitriptan | HTR1B | 131 | 26 | 27 | 15 | 25 | 9 | 53 | 16 |
| [DB00320](https://www.drugbank.ca/drugs/DB00320) | Dihydroergotamine | HTR1B | 131 | 26 | 27 | 15 | 25 | 9 | 53 | 16 |
| [DB00321](https://www.drugbank.ca/drugs/DB00321) | Amitriptyline | HTR1B | 131 | 26 | 27 | 15 | 25 | 9 | 53 | 16 |
| [DB00363](https://www.drugbank.ca/drugs/DB00363) | Clozapine | HTR1B | 131 | 26 | 27 | 15 | 25 | 9 | 53 | 16 |
| [DB00408](https://www.drugbank.ca/drugs/DB00408) | Loxapine | HTR1B | 131 | 26 | 27 | 15 | 25 | 9 | 53 | 16 |
| [DB00543](https://www.drugbank.ca/drugs/DB00543) | Amoxapine | HTR1B | 131 | 26 | 27 | 15 | 25 | 9 | 53 | 16 |
| [DB00571](https://www.drugbank.ca/drugs/DB00571) | Propranolol | HTR1B | 131 | 26 | 27 | 15 | 25 | 9 | 53 | 16 |
| [DB00589](https://www.drugbank.ca/drugs/DB00589) | Lisuride | HTR1B | 131 | 26 | 27 | 15 | 25 | 9 | 53 | 16 |
| [DB00669](https://www.drugbank.ca/drugs/DB00669) | Sumatriptan | HTR1B | 131 | 26 | 27 | 15 | 25 | 9 | 53 | 16 |
| [DB00696](https://www.drugbank.ca/drugs/DB00696) | Ergotamine | HTR1B | 131 | 26 | 27 | 15 | 25 | 9 | 53 | 16 |
| [DB00714](https://www.drugbank.ca/drugs/DB00714) | Apomorphine | HTR1B | 131 | 26 | 27 | 15 | 25 | 9 | 53 | 16 |
| [DB00904](https://www.drugbank.ca/drugs/DB00904) | Ondansetron | HTR1B | 131 | 26 | 27 | 15 | 25 | 9 | 53 | 16 |
| [DB00918](https://www.drugbank.ca/drugs/DB00918) | Almotriptan | HTR1B | 131 | 26 | 27 | 15 | 25 | 9 | 53 | 16 |
| [DB00952](https://www.drugbank.ca/drugs/DB00952) | Naratriptan | HTR1B | 131 | 26 | 27 | 15 | 25 | 9 | 53 | 16 |
| [DB00953](https://www.drugbank.ca/drugs/DB00953) | Rizatriptan | HTR1B | 131 | 26 | 27 | 15 | 25 | 9 | 53 | 16 |
| [DB00960](https://www.drugbank.ca/drugs/DB00960) | Pindolol | HTR1B | 131 | 26 | 27 | 15 | 25 | 9 | 53 | 16 |
| [DB00998](https://www.drugbank.ca/drugs/DB00998) | Frovatriptan | HTR1B | 131 | 26 | 27 | 15 | 25 | 9 | 53 | 16 |
| [DB01200](https://www.drugbank.ca/drugs/DB01200) | Bromocriptine | HTR1B | 131 | 26 | 27 | 15 | 25 | 9 | 53 | 16 |
| [DB01224](https://www.drugbank.ca/drugs/DB01224) | Quetiapine | HTR1B | 131 | 26 | 27 | 15 | 25 | 9 | 53 | 16 |
| [DB01238](https://www.drugbank.ca/drugs/DB01238) | Aripiprazole | HTR1B | 131 | 26 | 27 | 15 | 25 | 9 | 53 | 16 |
| [DB01359](https://www.drugbank.ca/drugs/DB01359) | Penbutolol | HTR1B | 131 | 26 | 27 | 15 | 25 | 9 | 53 | 16 |
| [DB01392](https://www.drugbank.ca/drugs/DB01392) | Yohimbine | HTR1B | 131 | 26 | 27 | 15 | 25 | 9 | 53 | 16 |
| [DB02325](https://www.drugbank.ca/drugs/DB02325) | Isopropyl alcohol | GCH1 | 131 | 29 | 15 | 14 | 21 | 6 | 73 | 24 |
| [DB06153](https://www.drugbank.ca/drugs/DB06153) | Pizotifen | HTR1B | 131 | 26 | 27 | 15 | 25 | 9 | 53 | 16 |
| [DB06216](https://www.drugbank.ca/drugs/DB06216) | Asenapine | HTR1B | 131 | 26 | 27 | 15 | 25 | 9 | 53 | 16 |
| [DB09068](https://www.drugbank.ca/drugs/DB09068) | Vortioxetine | HTR1B | 131 | 26 | 27 | 15 | 25 | 9 | 53 | 16 |
| [DB14185](https://www.drugbank.ca/drugs/DB14185) | Aripiprazole lauroxil | HTR1B | 131 | 26 | 27 | 15 | 25 | 9 | 53 | 16 |
| [DB00126](https://www.drugbank.ca/drugs/DB00126) | Ascorbic acid | TMLHE | 129 | 27 | 21 | 25 | 32 | 7 | 57 | 15 |
| [DB00139](https://www.drugbank.ca/drugs/DB00139) | Succinic acid | TMLHE | 129 | 27 | 21 | 25 | 32 | 7 | 57 | 15 |
| [DB01373](https://www.drugbank.ca/drugs/DB01373) | Calcium | ALPP | 123 | 25 | 14 | 14 | 17 | 9 | 48 | 18 |
| [DB06773](https://www.drugbank.ca/drugs/DB06773) | Human calcitonin | ACTN1 | 121 | 24 | 18 | 13 | 18 | 11 | 53 | 17 |
| [DB09130](https://www.drugbank.ca/drugs/DB09130) | Copper | ACTN1 | 121 | 24 | 18 | 13 | 18 | 11 | 53 | 17 |
| [DB00255](https://www.drugbank.ca/drugs/DB00255) | Diethylstilbestrol | ESRRG | 120 | 30 | 5 | 10 | 16 | 6 | 78 | 18 |
| [DB00675](https://www.drugbank.ca/drugs/DB00675) | Tamoxifen | ESRRG | 120 | 30 | 5 | 10 | 16 | 6 | 78 | 18 |
| [DB00783](https://www.drugbank.ca/drugs/DB00783) | Estradiol | ESRRG | 120 | 30 | 5 | 10 | 16 | 6 | 78 | 18 |
| [DB02659](https://www.drugbank.ca/drugs/DB02659) | Cholic Acid | ESRRG | 120 | 30 | 5 | 10 | 16 | 6 | 78 | 18 |
| [DB13952](https://www.drugbank.ca/drugs/DB13952) | Estradiol acetate | ESRRG | 120 | 30 | 5 | 10 | 16 | 6 | 78 | 18 |
| [DB13953](https://www.drugbank.ca/drugs/DB13953) | Estradiol benzoate | ESRRG | 120 | 30 | 5 | 10 | 16 | 6 | 78 | 18 |
| [DB13954](https://www.drugbank.ca/drugs/DB13954) | Estradiol cypionate | ESRRG | 120 | 30 | 5 | 10 | 16 | 6 | 78 | 18 |
| [DB13955](https://www.drugbank.ca/drugs/DB13955) | Estradiol dienanthate | ESRRG | 120 | 30 | 5 | 10 | 16 | 6 | 78 | 18 |
| [DB13956](https://www.drugbank.ca/drugs/DB13956) | Estradiol valerate | ESRRG | 120 | 30 | 5 | 10 | 16 | 6 | 78 | 18 |
| [DB01593](https://www.drugbank.ca/drugs/DB01593) | Zinc | CPN1 | 119 | 32 | 8 | 15 | 20 | 3 | 68 | 16 |
| [DB14487](https://www.drugbank.ca/drugs/DB14487) | Zinc acetate | CPN1 | 119 | 32 | 8 | 15 | 20 | 3 | 68 | 16 |
| [DB14533](https://www.drugbank.ca/drugs/DB14533) | Zinc chloride | CPN1 | 119 | 32 | 8 | 15 | 20 | 3 | 68 | 16 |
| [DB02709](https://www.drugbank.ca/drugs/DB02709) | Resveratrol | ITGA5 | 114 | 24 | 16 | 10 | 22 | 5 | 64 | 19 |
| [DB00157](https://www.drugbank.ca/drugs/DB00157) | NADH | MT-ND6 | 113 | 22 | 20 | 12 | 23 | 5 | 53 | 15 |
| [DB01373](https://www.drugbank.ca/drugs/DB01373) | Calcium | PDCD6 | 112 | 28 | 16 | 9 | 22 | 8 | 48 | 11 |
| [DB01593](https://www.drugbank.ca/drugs/DB01593) | Zinc | PDCD6 | 112 | 28 | 16 | 9 | 22 | 8 | 48 | 11 |
| [DB11093](https://www.drugbank.ca/drugs/DB11093) | Calcium Citrate | PDCD6 | 112 | 28 | 16 | 9 | 22 | 8 | 48 | 11 |
| [DB11348](https://www.drugbank.ca/drugs/DB11348) | Calcium Phosphate | PDCD6 | 112 | 28 | 16 | 9 | 22 | 8 | 48 | 11 |
| [DB14481](https://www.drugbank.ca/drugs/DB14481) | Calcium phosphate dihydrate | PDCD6 | 112 | 28 | 16 | 9 | 22 | 8 | 48 | 11 |
| [DB14487](https://www.drugbank.ca/drugs/DB14487) | Zinc acetate | PDCD6 | 112 | 28 | 16 | 9 | 22 | 8 | 48 | 11 |
| [DB14533](https://www.drugbank.ca/drugs/DB14533) | Zinc chloride | PDCD6 | 112 | 28 | 16 | 9 | 22 | 8 | 48 | 11 |
| [DB12010](https://www.drugbank.ca/drugs/DB12010) | Fostamatinib | SGK3 | 110 | 29 | 5 | 13 | 13 | 8 | 61 | 23 |
| [DB12010](https://www.drugbank.ca/drugs/DB12010) | Fostamatinib | EIF2AK4 | 108 | 22 | 20 | 13 | 15 | 5 | 54 | 13 |
| [DB00143](https://www.drugbank.ca/drugs/DB00143) | Glutathione | MGST3 | 107 | 28 | 6 | 16 | 18 | 3 | 66 | 16 |
| [DB01406](https://www.drugbank.ca/drugs/DB01406) | Danazol | CCL2 | 107 | 24 | 26 | 15 | 26 | 7 | 44 | 5 |
| [DB09301](https://www.drugbank.ca/drugs/DB09301) | Chondroitin sulfate | CCL2 | 107 | 24 | 26 | 15 | 26 | 7 | 44 | 5 |
| [DB00141](https://www.drugbank.ca/drugs/DB00141) | N-Acetylglucosamine | B4GALT3 | 106 | 12 | 13 | 12 | 14 | 5 | 56 | 17 |
| [DB00162](https://www.drugbank.ca/drugs/DB00162) | Vitamin A | RBP1 | 106 | 23 | 12 | 12 | 13 | 7 | 43 | 13 |
| [DB00459](https://www.drugbank.ca/drugs/DB00459) | Acitretin | RBP1 | 106 | 23 | 12 | 12 | 13 | 7 | 43 | 13 |
| [DB04941](https://www.drugbank.ca/drugs/DB04941) | Crofelemer | ANO1 | 106 | 16 | 11 | 13 | 14 | 11 | 55 | 8 |
| [DB00100](https://www.drugbank.ca/drugs/DB00100) | Coagulation Factor IX (Recombinant) | F11 | 105 | 21 | 13 | 10 | 19 | 5 | 44 | 8 |
| [DB00121](https://www.drugbank.ca/drugs/DB00121) | Biotin | ACACB | 105 | 16 | 24 | 9 | 23 | 5 | 42 | 10 |
| [DB00148](https://www.drugbank.ca/drugs/DB00148) | Creatine | CKM | 105 | 21 | 18 | 12 | 21 | 5 | 47 | 6 |
| [DB00173](https://www.drugbank.ca/drugs/DB00173) | Adenine | ACACB | 105 | 16 | 24 | 9 | 23 | 5 | 42 | 10 |
| [DB06404](https://www.drugbank.ca/drugs/DB06404) | Human C1-esterase inhibitor | F11 | 105 | 21 | 13 | 10 | 19 | 5 | 44 | 8 |
| [DB09228](https://www.drugbank.ca/drugs/DB09228) | Conestat alfa | F11 | 105 | 21 | 13 | 10 | 19 | 5 | 44 | 8 |
| [DB11300](https://www.drugbank.ca/drugs/DB11300) | Thrombin | F11 | 105 | 21 | 13 | 10 | 19 | 5 | 44 | 8 |
| [DB11571](https://www.drugbank.ca/drugs/DB11571) | Human Thrombin | F11 | 105 | 21 | 13 | 10 | 19 | 5 | 44 | 8 |
| [DB11572](https://www.drugbank.ca/drugs/DB11572) | Thrombin alfa | F11 | 105 | 21 | 13 | 10 | 19 | 5 | 44 | 8 |
| [DB13152](https://www.drugbank.ca/drugs/DB13152) | Coagulation Factor IX Human | F11 | 105 | 21 | 13 | 10 | 19 | 5 | 44 | 8 |
| [DB00025](https://www.drugbank.ca/drugs/DB00025) | Antihemophilic factor, human recombinant | LRP1 | 104 | 27 | 4 | 9 | 16 | 3 | 68 | 15 |
| [DB00031](https://www.drugbank.ca/drugs/DB00031) | Tenecteplase | LRP1 | 104 | 27 | 4 | 9 | 16 | 3 | 68 | 15 |
| [DB00100](https://www.drugbank.ca/drugs/DB00100) | Coagulation Factor IX (Recombinant) | LRP1 | 104 | 27 | 4 | 9 | 16 | 3 | 68 | 15 |
| [DB13152](https://www.drugbank.ca/drugs/DB13152) | Coagulation Factor IX Human | LRP1 | 104 | 27 | 4 | 9 | 16 | 3 | 68 | 15 |
| [DB13998](https://www.drugbank.ca/drugs/DB13998) | Lonoctocog alfa | LRP1 | 104 | 27 | 4 | 9 | 16 | 3 | 68 | 15 |
| [DB13999](https://www.drugbank.ca/drugs/DB13999) | Moroctocog alfa | LRP1 | 104 | 27 | 4 | 9 | 16 | 3 | 68 | 15 |
| [DB02709](https://www.drugbank.ca/drugs/DB02709) | Resveratrol | ALOX15 | 103 | 21 | 16 | 10 | 18 | 4 | 50 | 7 |
| [DB00419](https://www.drugbank.ca/drugs/DB00419) | Miglustat | UGCG | 102 | 29 | 4 | 13 | 17 | 5 | 74 | 13 |
| [DB09039](https://www.drugbank.ca/drugs/DB09039) | Eliglustat | UGCG | 102 | 29 | 4 | 13 | 17 | 5 | 74 | 13 |
| [DB00356](https://www.drugbank.ca/drugs/DB00356) | Chlorzoxazone | KCNMA1 | 101 | 18 | 3 | 7 | 12 | 2 | 57 | 24 |
| [DB00436](https://www.drugbank.ca/drugs/DB00436) | Bendroflumethiazide | KCNMA1 | 101 | 18 | 3 | 7 | 12 | 2 | 57 | 24 |
| [DB00721](https://www.drugbank.ca/drugs/DB00721) | Procaine | KCNMA1 | 101 | 18 | 3 | 7 | 12 | 2 | 57 | 24 |
| [DB00774](https://www.drugbank.ca/drugs/DB00774) | Hydroflumethiazide | KCNMA1 | 101 | 18 | 3 | 7 | 12 | 2 | 57 | 24 |
| [DB00999](https://www.drugbank.ca/drugs/DB00999) | Hydrochlorothiazide | KCNMA1 | 101 | 18 | 3 | 7 | 12 | 2 | 57 | 24 |
| [DB01003](https://www.drugbank.ca/drugs/DB01003) | Cromoglicic acid | KCNMA1 | 101 | 18 | 3 | 7 | 12 | 2 | 57 | 24 |
| [DB01110](https://www.drugbank.ca/drugs/DB01110) | Miconazole | KCNMA1 | 101 | 18 | 3 | 7 | 12 | 2 | 57 | 24 |
| [DB01119](https://www.drugbank.ca/drugs/DB01119) | Diazoxide | KCNMA1 | 101 | 18 | 3 | 7 | 12 | 2 | 57 | 24 |
| [DB01159](https://www.drugbank.ca/drugs/DB01159) | Halothane | KCNMA1 | 101 | 18 | 3 | 7 | 12 | 2 | 57 | 24 |
| [DB04209](https://www.drugbank.ca/drugs/DB04209) | Dequalinium | KCNMA1 | 101 | 18 | 3 | 7 | 12 | 2 | 57 | 24 |
| [DB09089](https://www.drugbank.ca/drugs/DB09089) | Trimebutine | KCNMA1 | 101 | 18 | 3 | 7 | 12 | 2 | 57 | 24 |
| [DB00149](https://www.drugbank.ca/drugs/DB00149) | L-Leucine | LCMT2 | 99 | 18 | 18 | 12 | 21 | 7 | 49 | 10 |
| [DB01373](https://www.drugbank.ca/drugs/DB01373) | Calcium | PCDH19 | 98 | 21 | 22 | 18 | 26 | 5 | 35 | 8 |
| [DB00242](https://www.drugbank.ca/drugs/DB00242) | Cladribine | PNP | 95 | 24 | 8 | 11 | 14 | 6 | 49 | 19 |
| [DB00900](https://www.drugbank.ca/drugs/DB00900) | Didanosine | PNP | 95 | 24 | 8 | 11 | 14 | 6 | 49 | 19 |
| [DB00126](https://www.drugbank.ca/drugs/DB00126) | Ascorbic acid | P3H1 | 93 | 20 | 4 | 11 | 14 | 3 | 48 | 23 |
| [DB00139](https://www.drugbank.ca/drugs/DB00139) | Succinic acid | P3H1 | 93 | 20 | 4 | 11 | 14 | 3 | 48 | 23 |
| [DB00172](https://www.drugbank.ca/drugs/DB00172) | Proline | P3H1 | 93 | 20 | 4 | 11 | 14 | 3 | 48 | 23 |
| [DB11601](https://www.drugbank.ca/drugs/DB11601) | Tuberculin Purified Protein Derivative | TLR2 | 91 | 16 | 20 | 15 | 21 | 6 | 38 | 10 |
| [DB00470](https://www.drugbank.ca/drugs/DB00470) | Dronabinol | CNR2 | 90 | 17 | 16 | 8 | 18 | 7 | 36 | 9 |
| [DB00486](https://www.drugbank.ca/drugs/DB00486) | Nabilone | CNR2 | 90 | 17 | 16 | 8 | 18 | 7 | 36 | 9 |
| [DB06202](https://www.drugbank.ca/drugs/DB06202) | Lasofoxifene | CNR2 | 90 | 17 | 16 | 8 | 18 | 7 | 36 | 9 |
| [DB09061](https://www.drugbank.ca/drugs/DB09061) | Cannabidiol | CNR2 | 90 | 17 | 16 | 8 | 18 | 7 | 36 | 9 |
| [DB00909](https://www.drugbank.ca/drugs/DB00909) | Zonisamide | CA13 | 88 | 25 | 3 | 4 | 8 | 8 | 52 | 20 |
| [DB00114](https://www.drugbank.ca/drugs/DB00114) | Pyridoxal phosphate | AADAT | 86 | 18 | 14 | 13 | 18 | 6 | 41 | 13 |
| [DB00142](https://www.drugbank.ca/drugs/DB00142) | Glutamic Acid | AADAT | 86 | 18 | 14 | 13 | 18 | 6 | 41 | 13 |
| [DB01064](https://www.drugbank.ca/drugs/DB01064) | Isoprenaline | PIK3R1 | 86 | 25 | 14 | 13 | 20 | 4 | 44 | 8 |
| [DB00163](https://www.drugbank.ca/drugs/DB00163) | Vitamin E | NR1I2 | 85 | 12 | 16 | 12 | 16 | 8 | 33 | 9 |
| [DB00239](https://www.drugbank.ca/drugs/DB00239) | Oxiconazole | NR1I2 | 85 | 12 | 16 | 12 | 16 | 8 | 33 | 9 |
| [DB00252](https://www.drugbank.ca/drugs/DB00252) | Phenytoin | NR1I2 | 85 | 12 | 16 | 12 | 16 | 8 | 33 | 9 |
| [DB00255](https://www.drugbank.ca/drugs/DB00255) | Diethylstilbestrol | NR1I2 | 85 | 12 | 16 | 12 | 16 | 8 | 33 | 9 |
| [DB00257](https://www.drugbank.ca/drugs/DB00257) | Clotrimazole | NR1I2 | 85 | 12 | 16 | 12 | 16 | 8 | 33 | 9 |
| [DB00312](https://www.drugbank.ca/drugs/DB00312) | Pentobarbital | NR1I2 | 85 | 12 | 16 | 12 | 16 | 8 | 33 | 9 |
| [DB00421](https://www.drugbank.ca/drugs/DB00421) | Spironolactone | NR1I2 | 85 | 12 | 16 | 12 | 16 | 8 | 33 | 9 |
| [DB00499](https://www.drugbank.ca/drugs/DB00499) | Flutamide | NR1I2 | 85 | 12 | 16 | 12 | 16 | 8 | 33 | 9 |
| [DB00503](https://www.drugbank.ca/drugs/DB00503) | Ritonavir | NR1I2 | 85 | 12 | 16 | 12 | 16 | 8 | 33 | 9 |
| [DB00530](https://www.drugbank.ca/drugs/DB00530) | Erlotinib | NR1I2 | 85 | 12 | 16 | 12 | 16 | 8 | 33 | 9 |
| [DB00531](https://www.drugbank.ca/drugs/DB00531) | Cyclophosphamide | NR1I2 | 85 | 12 | 16 | 12 | 16 | 8 | 33 | 9 |
| [DB00564](https://www.drugbank.ca/drugs/DB00564) | Carbamazepine | NR1I2 | 85 | 12 | 16 | 12 | 16 | 8 | 33 | 9 |
| [DB00675](https://www.drugbank.ca/drugs/DB00675) | Tamoxifen | NR1I2 | 85 | 12 | 16 | 12 | 16 | 8 | 33 | 9 |
| [DB00682](https://www.drugbank.ca/drugs/DB00682) | Warfarin | NR1I2 | 85 | 12 | 16 | 12 | 16 | 8 | 33 | 9 |
| [DB00754](https://www.drugbank.ca/drugs/DB00754) | Ethotoin | NR1I2 | 85 | 12 | 16 | 12 | 16 | 8 | 33 | 9 |
| [DB00783](https://www.drugbank.ca/drugs/DB00783) | Estradiol | NR1I2 | 85 | 12 | 16 | 12 | 16 | 8 | 33 | 9 |
| [DB00834](https://www.drugbank.ca/drugs/DB00834) | Mifepristone | NR1I2 | 85 | 12 | 16 | 12 | 16 | 8 | 33 | 9 |
| [DB00849](https://www.drugbank.ca/drugs/DB00849) | Methylphenobarbital | NR1I2 | 85 | 12 | 16 | 12 | 16 | 8 | 33 | 9 |
| [DB00977](https://www.drugbank.ca/drugs/DB00977) | Ethinylestradiol | NR1I2 | 85 | 12 | 16 | 12 | 16 | 8 | 33 | 9 |
| [DB01026](https://www.drugbank.ca/drugs/DB01026) | Ketoconazole | NR1I2 | 85 | 12 | 16 | 12 | 16 | 8 | 33 | 9 |
| [DB01039](https://www.drugbank.ca/drugs/DB01039) | Fenofibrate | NR1I2 | 85 | 12 | 16 | 12 | 16 | 8 | 33 | 9 |
| [DB01045](https://www.drugbank.ca/drugs/DB01045) | Rifampicin | NR1I2 | 85 | 12 | 16 | 12 | 16 | 8 | 33 | 9 |
| [DB01068](https://www.drugbank.ca/drugs/DB01068) | Clonazepam | NR1I2 | 85 | 12 | 16 | 12 | 16 | 8 | 33 | 9 |
| [DB01110](https://www.drugbank.ca/drugs/DB01110) | Miconazole | NR1I2 | 85 | 12 | 16 | 12 | 16 | 8 | 33 | 9 |
| [DB01115](https://www.drugbank.ca/drugs/DB01115) | Nifedipine | NR1I2 | 85 | 12 | 16 | 12 | 16 | 8 | 33 | 9 |
| [DB01127](https://www.drugbank.ca/drugs/DB01127) | Econazole | NR1I2 | 85 | 12 | 16 | 12 | 16 | 8 | 33 | 9 |
| [DB01138](https://www.drugbank.ca/drugs/DB01138) | Sulfinpyrazone | NR1I2 | 85 | 12 | 16 | 12 | 16 | 8 | 33 | 9 |
| [DB01174](https://www.drugbank.ca/drugs/DB01174) | Phenobarbital | NR1I2 | 85 | 12 | 16 | 12 | 16 | 8 | 33 | 9 |
| [DB01181](https://www.drugbank.ca/drugs/DB01181) | Ifosfamide | NR1I2 | 85 | 12 | 16 | 12 | 16 | 8 | 33 | 9 |
| [DB01220](https://www.drugbank.ca/drugs/DB01220) | Rifaximin | NR1I2 | 85 | 12 | 16 | 12 | 16 | 8 | 33 | 9 |
| [DB01229](https://www.drugbank.ca/drugs/DB01229) | Paclitaxel | NR1I2 | 85 | 12 | 16 | 12 | 16 | 8 | 33 | 9 |
| [DB01234](https://www.drugbank.ca/drugs/DB01234) | Dexamethasone | NR1I2 | 85 | 12 | 16 | 12 | 16 | 8 | 33 | 9 |
| [DB01248](https://www.drugbank.ca/drugs/DB01248) | Docetaxel | NR1I2 | 85 | 12 | 16 | 12 | 16 | 8 | 33 | 9 |
| [DB01393](https://www.drugbank.ca/drugs/DB01393) | Bezafibrate | NR1I2 | 85 | 12 | 16 | 12 | 16 | 8 | 33 | 9 |
| [DB01708](https://www.drugbank.ca/drugs/DB01708) | Prasterone | NR1I2 | 85 | 12 | 16 | 12 | 16 | 8 | 33 | 9 |
| [DB02709](https://www.drugbank.ca/drugs/DB02709) | Resveratrol | NR1I2 | 85 | 12 | 16 | 12 | 16 | 8 | 33 | 9 |
| [DB02789](https://www.drugbank.ca/drugs/DB02789) | Pregnenolone | NR1I2 | 85 | 12 | 16 | 12 | 16 | 8 | 33 | 9 |
| [DB04930](https://www.drugbank.ca/drugs/DB04930) | Permethrin | NR1I2 | 85 | 12 | 16 | 12 | 16 | 8 | 33 | 9 |
| [DB06777](https://www.drugbank.ca/drugs/DB06777) | Chenodeoxycholic acid | NR1I2 | 85 | 12 | 16 | 12 | 16 | 8 | 33 | 9 |
| [DB08604](https://www.drugbank.ca/drugs/DB08604) | Triclosan | NR1I2 | 85 | 12 | 16 | 12 | 16 | 8 | 33 | 9 |
| [DB08864](https://www.drugbank.ca/drugs/DB08864) | Rilpivirine | NR1I2 | 85 | 12 | 16 | 12 | 16 | 8 | 33 | 9 |
| [DB11087](https://www.drugbank.ca/drugs/DB11087) | Pyrethrum extract | NR1I2 | 85 | 12 | 16 | 12 | 16 | 8 | 33 | 9 |
| [DB11605](https://www.drugbank.ca/drugs/DB11605) | Myrrh | NR1I2 | 85 | 12 | 16 | 12 | 16 | 8 | 33 | 9 |
| [DB13179](https://www.drugbank.ca/drugs/DB13179) | Troleandomycin | NR1I2 | 85 | 12 | 16 | 12 | 16 | 8 | 33 | 9 |
| [DB13873](https://www.drugbank.ca/drugs/DB13873) | Fenofibric acid | NR1I2 | 85 | 12 | 16 | 12 | 16 | 8 | 33 | 9 |
| [DB13952](https://www.drugbank.ca/drugs/DB13952) | Estradiol acetate | NR1I2 | 85 | 12 | 16 | 12 | 16 | 8 | 33 | 9 |
| [DB13953](https://www.drugbank.ca/drugs/DB13953) | Estradiol benzoate | NR1I2 | 85 | 12 | 16 | 12 | 16 | 8 | 33 | 9 |
| [DB13954](https://www.drugbank.ca/drugs/DB13954) | Estradiol cypionate | NR1I2 | 85 | 12 | 16 | 12 | 16 | 8 | 33 | 9 |
| [DB13955](https://www.drugbank.ca/drugs/DB13955) | Estradiol dienanthate | NR1I2 | 85 | 12 | 16 | 12 | 16 | 8 | 33 | 9 |
| [DB13956](https://www.drugbank.ca/drugs/DB13956) | Estradiol valerate | NR1I2 | 85 | 12 | 16 | 12 | 16 | 8 | 33 | 9 |
| [DB14001](https://www.drugbank.ca/drugs/DB14001) | alpha-Tocopherol succinate | NR1I2 | 85 | 12 | 16 | 12 | 16 | 8 | 33 | 9 |
| [DB14002](https://www.drugbank.ca/drugs/DB14002) | D-alpha-Tocopherol acetate | NR1I2 | 85 | 12 | 16 | 12 | 16 | 8 | 33 | 9 |
| [DB00114](https://www.drugbank.ca/drugs/DB00114) | Pyridoxal phosphate | SPTLC2 | 84 | 23 | 13 | 11 | 17 | 6 | 44 | 2 |
| [DB00133](https://www.drugbank.ca/drugs/DB00133) | Serine | SPTLC2 | 84 | 23 | 13 | 11 | 17 | 6 | 44 | 2 |
| [DB11328](https://www.drugbank.ca/drugs/DB11328) | Tetradecyl hydrogen sulfate (ester) | PROCR | 84 | 18 | 20 | 16 | 21 | 4 | 31 | 3 |
| [DB00945](https://www.drugbank.ca/drugs/DB00945) | Acetylsalicylic acid | CASP1 | 80 | 15 | 1 | 1 | 6 | 4 | 49 | 17 |
| [DB01017](https://www.drugbank.ca/drugs/DB01017) | Minocycline | CASP1 | 80 | 15 | 1 | 1 | 6 | 4 | 49 | 17 |
| [DB00020](https://www.drugbank.ca/drugs/DB00020) | Sargramostim | CSF2RB | 74 | 15 | 20 | 13 | 19 | 4 | 21 | 6 |
| [DB08908](https://www.drugbank.ca/drugs/DB08908) | Dimethyl fumarate | RELA | 74 | 25 | 13 | 10 | 19 | 6 | 36 | 4 |
| [DB00004](https://www.drugbank.ca/drugs/DB00004) | Denileukin diftitox | IL2RG | 73 | 13 | 19 | 15 | 22 | 5 | 21 | 6 |
| [DB00041](https://www.drugbank.ca/drugs/DB00041) | Aldesleukin | IL2RG | 73 | 13 | 19 | 15 | 22 | 5 | 21 | 6 |
| [DB01169](https://www.drugbank.ca/drugs/DB01169) | Arsenic trioxide | TXNRD1 | 73 | 14 | 14 | 9 | 18 | 5 | 33 | 7 |
| [DB03147](https://www.drugbank.ca/drugs/DB03147) | Flavin adenine dinucleotide | TXNRD1 | 73 | 14 | 14 | 9 | 18 | 5 | 33 | 7 |
| [DB00130](https://www.drugbank.ca/drugs/DB00130) | L-Glutamine | CTPS1 | 68 | 20 | 9 | 7 | 9 | 9 | 41 | 9 |
| [DB00144](https://www.drugbank.ca/drugs/DB00144) | Phosphatidyl serine | PRKCA | 68 | 10 | 1 | 3 | 6 | 1 | 35 | 20 |
| [DB00163](https://www.drugbank.ca/drugs/DB00163) | Vitamin E | PRKCA | 68 | 10 | 1 | 3 | 6 | 1 | 35 | 20 |
| [DB00675](https://www.drugbank.ca/drugs/DB00675) | Tamoxifen | PRKCA | 68 | 10 | 1 | 3 | 6 | 1 | 35 | 20 |
| [DB01593](https://www.drugbank.ca/drugs/DB01593) | Zinc | MT3 | 68 | 13 | 4 | 6 | 8 | 0 | 33 | 15 |
| [DB05013](https://www.drugbank.ca/drugs/DB05013) | Ingenol mebutate | PRKCA | 68 | 10 | 1 | 3 | 6 | 1 | 35 | 20 |
| [DB06595](https://www.drugbank.ca/drugs/DB06595) | Midostaurin | PRKCA | 68 | 10 | 1 | 3 | 6 | 1 | 35 | 20 |
| [DB14001](https://www.drugbank.ca/drugs/DB14001) | alpha-Tocopherol succinate | PRKCA | 68 | 10 | 1 | 3 | 6 | 1 | 35 | 20 |
| [DB14002](https://www.drugbank.ca/drugs/DB14002) | D-alpha-Tocopherol acetate | PRKCA | 68 | 10 | 1 | 3 | 6 | 1 | 35 | 20 |
| [DB14487](https://www.drugbank.ca/drugs/DB14487) | Zinc acetate | MT3 | 68 | 13 | 4 | 6 | 8 | 0 | 33 | 15 |
| [DB14533](https://www.drugbank.ca/drugs/DB14533) | Zinc chloride | MT3 | 68 | 13 | 4 | 6 | 8 | 0 | 33 | 15 |
| [DB00157](https://www.drugbank.ca/drugs/DB00157) | NADH | GPD1 | 67 | 10 | 17 | 12 | 18 | 5 | 18 | 3 |
| [DB00331](https://www.drugbank.ca/drugs/DB00331) | Metformin | GPD1 | 67 | 10 | 17 | 12 | 18 | 5 | 18 | 3 |
| [DB01169](https://www.drugbank.ca/drugs/DB01169) | Arsenic trioxide | HDAC1 | 67 | 19 | 2 | 5 | 8 | 3 | 44 | 10 |
| [DB01593](https://www.drugbank.ca/drugs/DB01593) | Zinc | HDAC1 | 67 | 19 | 2 | 5 | 8 | 3 | 44 | 10 |
| [DB02546](https://www.drugbank.ca/drugs/DB02546) | Vorinostat | HDAC1 | 67 | 19 | 2 | 5 | 8 | 3 | 44 | 10 |
| [DB05015](https://www.drugbank.ca/drugs/DB05015) | Belinostat | HDAC1 | 67 | 19 | 2 | 5 | 8 | 3 | 44 | 10 |
| [DB06176](https://www.drugbank.ca/drugs/DB06176) | Romidepsin | HDAC1 | 67 | 19 | 2 | 5 | 8 | 3 | 44 | 10 |
| [DB06603](https://www.drugbank.ca/drugs/DB06603) | Panobinostat | HDAC1 | 67 | 19 | 2 | 5 | 8 | 3 | 44 | 10 |
| [DB08868](https://www.drugbank.ca/drugs/DB08868) | Fingolimod | HDAC1 | 67 | 19 | 2 | 5 | 8 | 3 | 44 | 10 |
| [DB14487](https://www.drugbank.ca/drugs/DB14487) | Zinc acetate | HDAC1 | 67 | 19 | 2 | 5 | 8 | 3 | 44 | 10 |
| [DB14533](https://www.drugbank.ca/drugs/DB14533) | Zinc chloride | HDAC1 | 67 | 19 | 2 | 5 | 8 | 3 | 44 | 10 |
| [DB00482](https://www.drugbank.ca/drugs/DB00482) | Celecoxib | PDPK1 | 66 | 20 | 1 | 1 | 5 | 3 | 47 | 7 |
| [DB00898](https://www.drugbank.ca/drugs/DB00898) | Ethanol | KCNJ9 | 66 | 12 | 4 | 5 | 7 | 0 | 31 | 15 |
| [DB11633](https://www.drugbank.ca/drugs/DB11633) | Isavuconazole | KCNJ9 | 66 | 12 | 4 | 5 | 7 | 0 | 31 | 15 |
| [DB12010](https://www.drugbank.ca/drugs/DB12010) | Fostamatinib | PDPK1 | 66 | 20 | 1 | 1 | 5 | 3 | 47 | 7 |
| [DB00157](https://www.drugbank.ca/drugs/DB00157) | NADH | MTHFD1 | 64 | 13 | 5 | 6 | 10 | 3 | 33 | 12 |
| [DB00160](https://www.drugbank.ca/drugs/DB00160) | L-Alanine | AARS2 | 64 | 11 | 13 | 10 | 16 | 1 | 25 | 3 |
| [DB02659](https://www.drugbank.ca/drugs/DB02659) | Cholic Acid | COX6B1 | 64 | 13 | 12 | 12 | 17 | 3 | 21 | 4 |
| [DB01593](https://www.drugbank.ca/drugs/DB01593) | Zinc | HPR | 62 | 10 | 6 | 7 | 7 | 3 | 26 | 12 |
| [DB09130](https://www.drugbank.ca/drugs/DB09130) | Copper | HPR | 62 | 10 | 6 | 7 | 7 | 3 | 26 | 12 |
| [DB14487](https://www.drugbank.ca/drugs/DB14487) | Zinc acetate | HPR | 62 | 10 | 6 | 7 | 7 | 3 | 26 | 12 |
| [DB14533](https://www.drugbank.ca/drugs/DB14533) | Zinc chloride | HPR | 62 | 10 | 6 | 7 | 7 | 3 | 26 | 12 |
| [DB12010](https://www.drugbank.ca/drugs/DB12010) | Fostamatinib | SRMS | 61 | 18 | 6 | 10 | 13 | 0 | 47 | 7 |
| [DB00119](https://www.drugbank.ca/drugs/DB00119) | Pyruvic acid | SLC16A3 | 60 | 8 | 20 | 15 | 15 | 4 | 26 | 5 |
| [DB00586](https://www.drugbank.ca/drugs/DB00586) | Diclofenac | ASIC1 | 58 | 11 | 4 | 5 | 7 | 0 | 25 | 14 |
| [DB00594](https://www.drugbank.ca/drugs/DB00594) | Amiloride | ASIC1 | 58 | 11 | 4 | 5 | 7 | 0 | 25 | 14 |
| [DB00162](https://www.drugbank.ca/drugs/DB00162) | Vitamin A | RDH14 | 55 | 19 | 3 | 2 | 8 | 4 | 27 | 7 |
| [DB12010](https://www.drugbank.ca/drugs/DB12010) | Fostamatinib | HIPK2 | 54 | 11 | 7 | 3 | 7 | 6 | 14 | 7 |
| [DB09401](https://www.drugbank.ca/drugs/DB09401) | Isosorbide | MCL1 | 53 | 7 | 15 | 8 | 17 | 3 | 23 | 5 |
| [DB12010](https://www.drugbank.ca/drugs/DB12010) | Fostamatinib | CTSS | 52 | 8 | 18 | 10 | 13 | 5 | 14 | 4 |
| [DB00198](https://www.drugbank.ca/drugs/DB00198) | Oseltamivir | CES1 | 51 | 8 | 5 | 7 | 7 | 3 | 19 | 11 |
| [DB00201](https://www.drugbank.ca/drugs/DB00201) | Caffeine | PIK3CD | 51 | 18 | 8 | 7 | 12 | 4 | 22 | 2 |
| [DB00454](https://www.drugbank.ca/drugs/DB00454) | Meperidine | CES1 | 51 | 8 | 5 | 7 | 7 | 3 | 19 | 11 |
| [DB00583](https://www.drugbank.ca/drugs/DB00583) | Levocarnitine | CES1 | 51 | 8 | 5 | 7 | 7 | 3 | 19 | 11 |
| [DB00907](https://www.drugbank.ca/drugs/DB00907) | Cocaine | CES1 | 51 | 8 | 5 | 7 | 7 | 3 | 19 | 11 |
| [DB01183](https://www.drugbank.ca/drugs/DB01183) | Naloxone | CES1 | 51 | 8 | 5 | 7 | 7 | 3 | 19 | 11 |
| [DB01452](https://www.drugbank.ca/drugs/DB01452) | Diamorphine | CES1 | 51 | 8 | 5 | 7 | 7 | 3 | 19 | 11 |
| [DB01599](https://www.drugbank.ca/drugs/DB01599) | Probucol | CES1 | 51 | 8 | 5 | 7 | 7 | 3 | 19 | 11 |
| [DB02659](https://www.drugbank.ca/drugs/DB02659) | Cholic Acid | CES1 | 51 | 8 | 5 | 7 | 7 | 3 | 19 | 11 |
| [DB04838](https://www.drugbank.ca/drugs/DB04838) | Cyclandelate | CES1 | 51 | 8 | 5 | 7 | 7 | 3 | 19 | 11 |
| [DB11952](https://www.drugbank.ca/drugs/DB11952) | Duvelisib | PIK3CD | 51 | 18 | 8 | 7 | 12 | 4 | 22 | 2 |
| [DB12010](https://www.drugbank.ca/drugs/DB12010) | Fostamatinib | PIK3CD | 51 | 18 | 8 | 7 | 12 | 4 | 22 | 2 |
| [DB12483](https://www.drugbank.ca/drugs/DB12483) | Copanlisib | PIK3CD | 51 | 18 | 8 | 7 | 12 | 4 | 22 | 2 |
| [DB09130](https://www.drugbank.ca/drugs/DB09130) | Copper | EIF4A1 | 50 | 9 | 11 | 9 | 8 | 4 | 21 | 4 |
| [DB00028](https://www.drugbank.ca/drugs/DB00028) | Immune Globulin Human | C3 | 49 | 9 | 3 | 7 | 7 | 4 | 20 | 4 |
| [DB01593](https://www.drugbank.ca/drugs/DB01593) | Zinc | C3 | 49 | 9 | 3 | 7 | 7 | 4 | 20 | 4 |
| [DB09130](https://www.drugbank.ca/drugs/DB09130) | Copper | C3 | 49 | 9 | 3 | 7 | 7 | 4 | 20 | 4 |
| [DB14487](https://www.drugbank.ca/drugs/DB14487) | Zinc acetate | C3 | 49 | 9 | 3 | 7 | 7 | 4 | 20 | 4 |
| [DB14533](https://www.drugbank.ca/drugs/DB14533) | Zinc chloride | C3 | 49 | 9 | 3 | 7 | 7 | 4 | 20 | 4 |
| [DB00152](https://www.drugbank.ca/drugs/DB00152) | Thiamine | SLC19A2 | 47 | 12 | 3 | 5 | 8 | 0 | 29 | 3 |
| [DB12010](https://www.drugbank.ca/drugs/DB12010) | Fostamatinib | CLASP1 | 47 | 11 | 11 | 8 | 14 | 2 | 20 | 0 |
| [DB00143](https://www.drugbank.ca/drugs/DB00143) | Glutathione | GSTO1 | 45 | 11 | 12 | 5 | 13 | 4 | 17 | 0 |
| [DB00119](https://www.drugbank.ca/drugs/DB00119) | Pyruvic acid | PKM | 44 | 9 | 5 | 3 | 6 | 2 | 24 | 3 |
| [DB09130](https://www.drugbank.ca/drugs/DB09130) | Copper | PKM | 44 | 9 | 5 | 3 | 6 | 2 | 24 | 3 |
| [DB09130](https://www.drugbank.ca/drugs/DB09130) | Copper | HSPD1 | 42 | 9 | 4 | 5 | 6 | 2 | 23 | 5 |
| [DB00114](https://www.drugbank.ca/drugs/DB00114) | Pyridoxal phosphate | ALAS1 | 41 | 8 | 1 | 2 | 6 | 2 | 27 | 7 |
| [DB00114](https://www.drugbank.ca/drugs/DB00114) | Pyridoxal phosphate | ALAS1 | 41 | 8 | 1 | 2 | 6 | 2 | 27 | 7 |
| [DB00145](https://www.drugbank.ca/drugs/DB00145) | Glycine | ALAS1 | 41 | 8 | 1 | 2 | 6 | 2 | 27 | 7 |
| [DB00795](https://www.drugbank.ca/drugs/DB00795) | Sulfasalazine | ACAT1 | 41 | 6 | 7 | 4 | 7 | 4 | 11 | 3 |
| [DB00122](https://www.drugbank.ca/drugs/DB00122) | Choline | PHOSPHO1 | 40 | 12 | 4 | 1 | 4 | 4 | 22 | 8 |
| [DB14006](https://www.drugbank.ca/drugs/DB14006) | Choline salicylate | PHOSPHO1 | 40 | 12 | 4 | 1 | 4 | 4 | 22 | 8 |
| [DB00157](https://www.drugbank.ca/drugs/DB00157) | NADH | IDH3A | 39 | 6 | 11 | 6 | 9 | 1 | 16 | 2 |
| [DB06757](https://www.drugbank.ca/drugs/DB06757) | Manganese | IDH3A | 39 | 6 | 11 | 6 | 9 | 1 | 16 | 2 |
| [DB09130](https://www.drugbank.ca/drugs/DB09130) | Copper | IDH3A | 39 | 6 | 11 | 6 | 9 | 1 | 16 | 2 |
| [DB00123](https://www.drugbank.ca/drugs/DB00123) | L-Lysine | SLC7A1 | 38 | 8 | 1 | 4 | 7 | 2 | 17 | 10 |
| [DB00125](https://www.drugbank.ca/drugs/DB00125) | L-Arginine | SLC7A1 | 38 | 8 | 1 | 4 | 7 | 2 | 17 | 10 |
| [DB00129](https://www.drugbank.ca/drugs/DB00129) | Ornithine | SLC7A1 | 38 | 8 | 1 | 4 | 7 | 2 | 17 | 10 |
| [DB00215](https://www.drugbank.ca/drugs/DB00215) | Citalopram | HRH1 | 36 | 7 | 12 | 5 | 10 | 2 | 12 | 0 |
| [DB00245](https://www.drugbank.ca/drugs/DB00245) | Benzatropine | HRH1 | 36 | 7 | 12 | 5 | 10 | 2 | 12 | 0 |
| [DB00246](https://www.drugbank.ca/drugs/DB00246) | Ziprasidone | HRH1 | 36 | 7 | 12 | 5 | 10 | 2 | 12 | 0 |
| [DB00283](https://www.drugbank.ca/drugs/DB00283) | Clemastine | HRH1 | 36 | 7 | 12 | 5 | 10 | 2 | 12 | 0 |
| [DB00321](https://www.drugbank.ca/drugs/DB00321) | Amitriptyline | HRH1 | 36 | 7 | 12 | 5 | 10 | 2 | 12 | 0 |
| [DB00334](https://www.drugbank.ca/drugs/DB00334) | Olanzapine | HRH1 | 36 | 7 | 12 | 5 | 10 | 2 | 12 | 0 |
| [DB00341](https://www.drugbank.ca/drugs/DB00341) | Cetirizine | HRH1 | 36 | 7 | 12 | 5 | 10 | 2 | 12 | 0 |
| [DB00354](https://www.drugbank.ca/drugs/DB00354) | Buclizine | HRH1 | 36 | 7 | 12 | 5 | 10 | 2 | 12 | 0 |
| [DB00363](https://www.drugbank.ca/drugs/DB00363) | Clozapine | HRH1 | 36 | 7 | 12 | 5 | 10 | 2 | 12 | 0 |
| [DB00366](https://www.drugbank.ca/drugs/DB00366) | Doxylamine | HRH1 | 36 | 7 | 12 | 5 | 10 | 2 | 12 | 0 |
| [DB00370](https://www.drugbank.ca/drugs/DB00370) | Mirtazapine | HRH1 | 36 | 7 | 12 | 5 | 10 | 2 | 12 | 0 |
| [DB00405](https://www.drugbank.ca/drugs/DB00405) | Dexbrompheniramine | HRH1 | 36 | 7 | 12 | 5 | 10 | 2 | 12 | 0 |
| [DB00408](https://www.drugbank.ca/drugs/DB00408) | Loxapine | HRH1 | 36 | 7 | 12 | 5 | 10 | 2 | 12 | 0 |
| [DB00420](https://www.drugbank.ca/drugs/DB00420) | Promazine | HRH1 | 36 | 7 | 12 | 5 | 10 | 2 | 12 | 0 |
| [DB00427](https://www.drugbank.ca/drugs/DB00427) | Triprolidine | HRH1 | 36 | 7 | 12 | 5 | 10 | 2 | 12 | 0 |
| [DB00433](https://www.drugbank.ca/drugs/DB00433) | Prochlorperazine | HRH1 | 36 | 7 | 12 | 5 | 10 | 2 | 12 | 0 |
| [DB00434](https://www.drugbank.ca/drugs/DB00434) | Cyproheptadine | HRH1 | 36 | 7 | 12 | 5 | 10 | 2 | 12 | 0 |
| [DB00455](https://www.drugbank.ca/drugs/DB00455) | Loratadine | HRH1 | 36 | 7 | 12 | 5 | 10 | 2 | 12 | 0 |
| [DB00458](https://www.drugbank.ca/drugs/DB00458) | Imipramine | HRH1 | 36 | 7 | 12 | 5 | 10 | 2 | 12 | 0 |
| [DB00477](https://www.drugbank.ca/drugs/DB00477) | Chlorpromazine | HRH1 | 36 | 7 | 12 | 5 | 10 | 2 | 12 | 0 |
| [DB00540](https://www.drugbank.ca/drugs/DB00540) | Nortriptyline | HRH1 | 36 | 7 | 12 | 5 | 10 | 2 | 12 | 0 |
| [DB00543](https://www.drugbank.ca/drugs/DB00543) | Amoxapine | HRH1 | 36 | 7 | 12 | 5 | 10 | 2 | 12 | 0 |
| [DB00555](https://www.drugbank.ca/drugs/DB00555) | Lamotrigine | HRH1 | 36 | 7 | 12 | 5 | 10 | 2 | 12 | 0 |
| [DB00557](https://www.drugbank.ca/drugs/DB00557) | Hydroxyzine | HRH1 | 36 | 7 | 12 | 5 | 10 | 2 | 12 | 0 |
| [DB00568](https://www.drugbank.ca/drugs/DB00568) | Cinnarizine | HRH1 | 36 | 7 | 12 | 5 | 10 | 2 | 12 | 0 |
| [DB00656](https://www.drugbank.ca/drugs/DB00656) | Trazodone | HRH1 | 36 | 7 | 12 | 5 | 10 | 2 | 12 | 0 |
| [DB00719](https://www.drugbank.ca/drugs/DB00719) | Azatadine | HRH1 | 36 | 7 | 12 | 5 | 10 | 2 | 12 | 0 |
| [DB00726](https://www.drugbank.ca/drugs/DB00726) | Trimipramine | HRH1 | 36 | 7 | 12 | 5 | 10 | 2 | 12 | 0 |
| [DB00734](https://www.drugbank.ca/drugs/DB00734) | Risperidone | HRH1 | 36 | 7 | 12 | 5 | 10 | 2 | 12 | 0 |
| [DB00737](https://www.drugbank.ca/drugs/DB00737) | Meclizine | HRH1 | 36 | 7 | 12 | 5 | 10 | 2 | 12 | 0 |
| [DB00748](https://www.drugbank.ca/drugs/DB00748) | Carbinoxamine | HRH1 | 36 | 7 | 12 | 5 | 10 | 2 | 12 | 0 |
| [DB00751](https://www.drugbank.ca/drugs/DB00751) | Epinastine | HRH1 | 36 | 7 | 12 | 5 | 10 | 2 | 12 | 0 |
| [DB00768](https://www.drugbank.ca/drugs/DB00768) | Olopatadine | HRH1 | 36 | 7 | 12 | 5 | 10 | 2 | 12 | 0 |
| [DB00777](https://www.drugbank.ca/drugs/DB00777) | Propiomazine | HRH1 | 36 | 7 | 12 | 5 | 10 | 2 | 12 | 0 |
| [DB00792](https://www.drugbank.ca/drugs/DB00792) | Tripelennamine | HRH1 | 36 | 7 | 12 | 5 | 10 | 2 | 12 | 0 |
| [DB00797](https://www.drugbank.ca/drugs/DB00797) | Tolazoline | HRH1 | 36 | 7 | 12 | 5 | 10 | 2 | 12 | 0 |
| [DB00835](https://www.drugbank.ca/drugs/DB00835) | Brompheniramine | HRH1 | 36 | 7 | 12 | 5 | 10 | 2 | 12 | 0 |
| [DB00902](https://www.drugbank.ca/drugs/DB00902) | Methdilazine | HRH1 | 36 | 7 | 12 | 5 | 10 | 2 | 12 | 0 |
| [DB00920](https://www.drugbank.ca/drugs/DB00920) | Ketotifen | HRH1 | 36 | 7 | 12 | 5 | 10 | 2 | 12 | 0 |
| [DB00934](https://www.drugbank.ca/drugs/DB00934) | Maprotiline | HRH1 | 36 | 7 | 12 | 5 | 10 | 2 | 12 | 0 |
| [DB00950](https://www.drugbank.ca/drugs/DB00950) | Fexofenadine | HRH1 | 36 | 7 | 12 | 5 | 10 | 2 | 12 | 0 |
| [DB00967](https://www.drugbank.ca/drugs/DB00967) | Desloratadine | HRH1 | 36 | 7 | 12 | 5 | 10 | 2 | 12 | 0 |
| [DB00972](https://www.drugbank.ca/drugs/DB00972) | Azelastine | HRH1 | 36 | 7 | 12 | 5 | 10 | 2 | 12 | 0 |
| [DB00985](https://www.drugbank.ca/drugs/DB00985) | Dimenhydrinate | HRH1 | 36 | 7 | 12 | 5 | 10 | 2 | 12 | 0 |
| [DB01069](https://www.drugbank.ca/drugs/DB01069) | Promethazine | HRH1 | 36 | 7 | 12 | 5 | 10 | 2 | 12 | 0 |
| [DB01075](https://www.drugbank.ca/drugs/DB01075) | Diphenhydramine | HRH1 | 36 | 7 | 12 | 5 | 10 | 2 | 12 | 0 |
| [DB01084](https://www.drugbank.ca/drugs/DB01084) | Emedastine | HRH1 | 36 | 7 | 12 | 5 | 10 | 2 | 12 | 0 |
| [DB01106](https://www.drugbank.ca/drugs/DB01106) | Levocabastine | HRH1 | 36 | 7 | 12 | 5 | 10 | 2 | 12 | 0 |
| [DB01114](https://www.drugbank.ca/drugs/DB01114) | Chlorphenamine | HRH1 | 36 | 7 | 12 | 5 | 10 | 2 | 12 | 0 |
| [DB01142](https://www.drugbank.ca/drugs/DB01142) | Doxepin | HRH1 | 36 | 7 | 12 | 5 | 10 | 2 | 12 | 0 |
| [DB01146](https://www.drugbank.ca/drugs/DB01146) | Diphenylpyraline | HRH1 | 36 | 7 | 12 | 5 | 10 | 2 | 12 | 0 |
| [DB01151](https://www.drugbank.ca/drugs/DB01151) | Desipramine | HRH1 | 36 | 7 | 12 | 5 | 10 | 2 | 12 | 0 |
| [DB01173](https://www.drugbank.ca/drugs/DB01173) | Orphenadrine | HRH1 | 36 | 7 | 12 | 5 | 10 | 2 | 12 | 0 |
| [DB01175](https://www.drugbank.ca/drugs/DB01175) | Escitalopram | HRH1 | 36 | 7 | 12 | 5 | 10 | 2 | 12 | 0 |
| [DB01176](https://www.drugbank.ca/drugs/DB01176) | Cyclizine | HRH1 | 36 | 7 | 12 | 5 | 10 | 2 | 12 | 0 |
| [DB01224](https://www.drugbank.ca/drugs/DB01224) | Quetiapine | HRH1 | 36 | 7 | 12 | 5 | 10 | 2 | 12 | 0 |
| [DB01237](https://www.drugbank.ca/drugs/DB01237) | Bromodiphenhydramine | HRH1 | 36 | 7 | 12 | 5 | 10 | 2 | 12 | 0 |
| [DB01238](https://www.drugbank.ca/drugs/DB01238) | Aripiprazole | HRH1 | 36 | 7 | 12 | 5 | 10 | 2 | 12 | 0 |
| [DB01246](https://www.drugbank.ca/drugs/DB01246) | Alimemazine | HRH1 | 36 | 7 | 12 | 5 | 10 | 2 | 12 | 0 |
| [DB01267](https://www.drugbank.ca/drugs/DB01267) | Paliperidone | HRH1 | 36 | 7 | 12 | 5 | 10 | 2 | 12 | 0 |
| [DB01403](https://www.drugbank.ca/drugs/DB01403) | Methotrimeprazine | HRH1 | 36 | 7 | 12 | 5 | 10 | 2 | 12 | 0 |
| [DB01619](https://www.drugbank.ca/drugs/DB01619) | Phenindamine | HRH1 | 36 | 7 | 12 | 5 | 10 | 2 | 12 | 0 |
| [DB01620](https://www.drugbank.ca/drugs/DB01620) | Pheniramine | HRH1 | 36 | 7 | 12 | 5 | 10 | 2 | 12 | 0 |
| [DB01624](https://www.drugbank.ca/drugs/DB01624) | Zuclopenthixol | HRH1 | 36 | 7 | 12 | 5 | 10 | 2 | 12 | 0 |
| [DB04841](https://www.drugbank.ca/drugs/DB04841) | Flunarizine | HRH1 | 36 | 7 | 12 | 5 | 10 | 2 | 12 | 0 |
| [DB04890](https://www.drugbank.ca/drugs/DB04890) | Bepotastine | HRH1 | 36 | 7 | 12 | 5 | 10 | 2 | 12 | 0 |
| [DB04946](https://www.drugbank.ca/drugs/DB04946) | Iloperidone | HRH1 | 36 | 7 | 12 | 5 | 10 | 2 | 12 | 0 |
| [DB05381](https://www.drugbank.ca/drugs/DB05381) | Histamine | HRH1 | 36 | 7 | 12 | 5 | 10 | 2 | 12 | 0 |
| [DB06016](https://www.drugbank.ca/drugs/DB06016) | Cariprazine | HRH1 | 36 | 7 | 12 | 5 | 10 | 2 | 12 | 0 |
| [DB06148](https://www.drugbank.ca/drugs/DB06148) | Mianserin | HRH1 | 36 | 7 | 12 | 5 | 10 | 2 | 12 | 0 |
| [DB06153](https://www.drugbank.ca/drugs/DB06153) | Pizotifen | HRH1 | 36 | 7 | 12 | 5 | 10 | 2 | 12 | 0 |
| [DB06216](https://www.drugbank.ca/drugs/DB06216) | Asenapine | HRH1 | 36 | 7 | 12 | 5 | 10 | 2 | 12 | 0 |
| [DB06282](https://www.drugbank.ca/drugs/DB06282) | Levocetirizine | HRH1 | 36 | 7 | 12 | 5 | 10 | 2 | 12 | 0 |
| [DB06691](https://www.drugbank.ca/drugs/DB06691) | Mepyramine | HRH1 | 36 | 7 | 12 | 5 | 10 | 2 | 12 | 0 |
| [DB06698](https://www.drugbank.ca/drugs/DB06698) | Betahistine | HRH1 | 36 | 7 | 12 | 5 | 10 | 2 | 12 | 0 |
| [DB06766](https://www.drugbank.ca/drugs/DB06766) | Alcaftadine | HRH1 | 36 | 7 | 12 | 5 | 10 | 2 | 12 | 0 |
| [DB08799](https://www.drugbank.ca/drugs/DB08799) | Antazoline | HRH1 | 36 | 7 | 12 | 5 | 10 | 2 | 12 | 0 |
| [DB08801](https://www.drugbank.ca/drugs/DB08801) | Dimetindene | HRH1 | 36 | 7 | 12 | 5 | 10 | 2 | 12 | 0 |
| [DB08936](https://www.drugbank.ca/drugs/DB08936) | Chlorcyclizine | HRH1 | 36 | 7 | 12 | 5 | 10 | 2 | 12 | 0 |
| [DB09016](https://www.drugbank.ca/drugs/DB09016) | Butriptyline | HRH1 | 36 | 7 | 12 | 5 | 10 | 2 | 12 | 0 |
| [DB09167](https://www.drugbank.ca/drugs/DB09167) | Dosulepin | HRH1 | 36 | 7 | 12 | 5 | 10 | 2 | 12 | 0 |
| [DB09195](https://www.drugbank.ca/drugs/DB09195) | Lorpiprazole | HRH1 | 36 | 7 | 12 | 5 | 10 | 2 | 12 | 0 |
| [DB09488](https://www.drugbank.ca/drugs/DB09488) | Acrivastine | HRH1 | 36 | 7 | 12 | 5 | 10 | 2 | 12 | 0 |
| [DB09555](https://www.drugbank.ca/drugs/DB09555) | Dexchlorpheniramine maleate | HRH1 | 36 | 7 | 12 | 5 | 10 | 2 | 12 | 0 |
| [DB11235](https://www.drugbank.ca/drugs/DB11235) | Thonzylamine | HRH1 | 36 | 7 | 12 | 5 | 10 | 2 | 12 | 0 |
| [DB11591](https://www.drugbank.ca/drugs/DB11591) | Bilastine | HRH1 | 36 | 7 | 12 | 5 | 10 | 2 | 12 | 0 |
| [DB11614](https://www.drugbank.ca/drugs/DB11614) | Rupatadine | HRH1 | 36 | 7 | 12 | 5 | 10 | 2 | 12 | 0 |
| [DB12010](https://www.drugbank.ca/drugs/DB12010) | Fostamatinib | CAMK2A | 36 | 11 | 1 | 2 | 4 | 2 | 19 | 8 |
| [DB14185](https://www.drugbank.ca/drugs/DB14185) | Aripiprazole lauroxil | HRH1 | 36 | 7 | 12 | 5 | 10 | 2 | 12 | 0 |
| [DB12010](https://www.drugbank.ca/drugs/DB12010) | Fostamatinib | DAPK2 | 35 | 5 | 0 | 4 | 7 | 0 | 22 | 3 |
| [DB00231](https://www.drugbank.ca/drugs/DB00231) | Temazepam | GABRA5 | 34 | 9 | 2 | 1 | 4 | 0 | 20 | 6 |
| [DB00241](https://www.drugbank.ca/drugs/DB00241) | Butalbital | GABRA5 | 34 | 9 | 2 | 1 | 4 | 0 | 20 | 6 |
| [DB00306](https://www.drugbank.ca/drugs/DB00306) | Talbutal | GABRA5 | 34 | 9 | 2 | 1 | 4 | 0 | 20 | 6 |
| [DB00312](https://www.drugbank.ca/drugs/DB00312) | Pentobarbital | GABRA5 | 34 | 9 | 2 | 1 | 4 | 0 | 20 | 6 |
| [DB00371](https://www.drugbank.ca/drugs/DB00371) | Meprobamate | GABRA5 | 34 | 9 | 2 | 1 | 4 | 0 | 20 | 6 |
| [DB00395](https://www.drugbank.ca/drugs/DB00395) | Carisoprodol | GABRA5 | 34 | 9 | 2 | 1 | 4 | 0 | 20 | 6 |
| [DB00402](https://www.drugbank.ca/drugs/DB00402) | Eszopiclone | GABRA5 | 34 | 9 | 2 | 1 | 4 | 0 | 20 | 6 |
| [DB00404](https://www.drugbank.ca/drugs/DB00404) | Alprazolam | GABRA5 | 34 | 9 | 2 | 1 | 4 | 0 | 20 | 6 |
| [DB00418](https://www.drugbank.ca/drugs/DB00418) | Secobarbital | GABRA5 | 34 | 9 | 2 | 1 | 4 | 0 | 20 | 6 |
| [DB00599](https://www.drugbank.ca/drugs/DB00599) | Thiopental | GABRA5 | 34 | 9 | 2 | 1 | 4 | 0 | 20 | 6 |
| [DB00683](https://www.drugbank.ca/drugs/DB00683) | Midazolam | GABRA5 | 34 | 9 | 2 | 1 | 4 | 0 | 20 | 6 |
| [DB00690](https://www.drugbank.ca/drugs/DB00690) | Flurazepam | GABRA5 | 34 | 9 | 2 | 1 | 4 | 0 | 20 | 6 |
| [DB00794](https://www.drugbank.ca/drugs/DB00794) | Primidone | GABRA5 | 34 | 9 | 2 | 1 | 4 | 0 | 20 | 6 |
| [DB00829](https://www.drugbank.ca/drugs/DB00829) | Diazepam | GABRA5 | 34 | 9 | 2 | 1 | 4 | 0 | 20 | 6 |
| [DB00842](https://www.drugbank.ca/drugs/DB00842) | Oxazepam | GABRA5 | 34 | 9 | 2 | 1 | 4 | 0 | 20 | 6 |
| [DB00849](https://www.drugbank.ca/drugs/DB00849) | Methylphenobarbital | GABRA5 | 34 | 9 | 2 | 1 | 4 | 0 | 20 | 6 |
| [DB00897](https://www.drugbank.ca/drugs/DB00897) | Triazolam | GABRA5 | 34 | 9 | 2 | 1 | 4 | 0 | 20 | 6 |
| [DB00898](https://www.drugbank.ca/drugs/DB00898) | Ethanol | GABRA5 | 34 | 9 | 2 | 1 | 4 | 0 | 20 | 6 |
| [DB01198](https://www.drugbank.ca/drugs/DB01198) | Zopiclone | GABRA5 | 34 | 9 | 2 | 1 | 4 | 0 | 20 | 6 |
| [DB01205](https://www.drugbank.ca/drugs/DB01205) | Flumazenil | GABRA5 | 34 | 9 | 2 | 1 | 4 | 0 | 20 | 6 |
| [DB01215](https://www.drugbank.ca/drugs/DB01215) | Estazolam | GABRA5 | 34 | 9 | 2 | 1 | 4 | 0 | 20 | 6 |
| [DB01351](https://www.drugbank.ca/drugs/DB01351) | Amobarbital | GABRA5 | 34 | 9 | 2 | 1 | 4 | 0 | 20 | 6 |
| [DB01353](https://www.drugbank.ca/drugs/DB01353) | Butobarbital | GABRA5 | 34 | 9 | 2 | 1 | 4 | 0 | 20 | 6 |
| [DB01544](https://www.drugbank.ca/drugs/DB01544) | Flunitrazepam | GABRA5 | 34 | 9 | 2 | 1 | 4 | 0 | 20 | 6 |
| [DB01558](https://www.drugbank.ca/drugs/DB01558) | Bromazepam | GABRA5 | 34 | 9 | 2 | 1 | 4 | 0 | 20 | 6 |
| [DB01559](https://www.drugbank.ca/drugs/DB01559) | Clotiazepam | GABRA5 | 34 | 9 | 2 | 1 | 4 | 0 | 20 | 6 |
| [DB01588](https://www.drugbank.ca/drugs/DB01588) | Prazepam | GABRA5 | 34 | 9 | 2 | 1 | 4 | 0 | 20 | 6 |
| [DB01589](https://www.drugbank.ca/drugs/DB01589) | Quazepam | GABRA5 | 34 | 9 | 2 | 1 | 4 | 0 | 20 | 6 |
| [DB01595](https://www.drugbank.ca/drugs/DB01595) | Nitrazepam | GABRA5 | 34 | 9 | 2 | 1 | 4 | 0 | 20 | 6 |
| [DB03147](https://www.drugbank.ca/drugs/DB03147) | Flavin adenine dinucleotide | ERO1B | 34 | 9 | 10 | 4 | 11 | 2 | 14 | 3 |
| [DB13872](https://www.drugbank.ca/drugs/DB13872) | Lormetazepam | GABRA5 | 34 | 9 | 2 | 1 | 4 | 0 | 20 | 6 |
| [DB09130](https://www.drugbank.ca/drugs/DB09130) | Copper | HEPHL1 | 33 | 7 | 10 | 3 | 8 | 2 | 12 | 0 |
| [DB09130](https://www.drugbank.ca/drugs/DB09130) | Copper | LUM | 33 | 7 | 8 | 3 | 6 | 4 | 12 | 4 |
| [DB00114](https://www.drugbank.ca/drugs/DB00114) | Pyridoxal phosphate | GAD2 | 32 | 7 | 1 | 3 | 4 | 4 | 12 | 5 |
| [DB00142](https://www.drugbank.ca/drugs/DB00142) | Glutamic Acid | GAD2 | 32 | 7 | 1 | 3 | 4 | 4 | 12 | 5 |
| [DB00131](https://www.drugbank.ca/drugs/DB00131) | Adenosine phosphate | ACSL1 | 31 | 7 | 7 | 1 | 5 | 2 | 9 | 2 |
| [DB00158](https://www.drugbank.ca/drugs/DB00158) | Folic acid | FOLR1 | 30 | 7 | 2 | 5 | 5 | 0 | 13 | 3 |
| [DB00855](https://www.drugbank.ca/drugs/DB00855) | Aminolevulinic acid | ALAD | 30 | 5 | 1 | 1 | 3 | 0 | 16 | 9 |
| [DB00118](https://www.drugbank.ca/drugs/DB00118) | Ademetionine | MAT1A | 29 | 6 | 0 | 3 | 3 | 3 | 10 | 6 |
| [DB09130](https://www.drugbank.ca/drugs/DB09130) | Copper | CBX5 | 29 | 9 | 5 | 6 | 8 | 1 | 14 | 2 |
| [DB00210](https://www.drugbank.ca/drugs/DB00210) | Adapalene | RARA | 28 | 6 | 1 | 2 | 4 | 1 | 17 | 5 |
| [DB00459](https://www.drugbank.ca/drugs/DB00459) | Acitretin | RARA | 28 | 6 | 1 | 2 | 4 | 1 | 17 | 5 |
| [DB00523](https://www.drugbank.ca/drugs/DB00523) | Alitretinoin | RARA | 28 | 6 | 1 | 2 | 4 | 1 | 17 | 5 |
| [DB00755](https://www.drugbank.ca/drugs/DB00755) | Tretinoin | RARA | 28 | 6 | 1 | 2 | 4 | 1 | 17 | 5 |
| [DB00799](https://www.drugbank.ca/drugs/DB00799) | Tazarotene | RARA | 28 | 6 | 1 | 2 | 4 | 1 | 17 | 5 |
| [DB00982](https://www.drugbank.ca/drugs/DB00982) | Isotretinoin | RARA | 28 | 6 | 1 | 2 | 4 | 1 | 17 | 5 |
| [DB01373](https://www.drugbank.ca/drugs/DB01373) | Calcium | CAST | 27 | 3 | 7 | 5 | 6 | 0 | 11 | 1 |
| [DB08890](https://www.drugbank.ca/drugs/DB08890) | Linaclotide | GUCY2C | 27 | 6 | 2 | 3 | 3 | 0 | 12 | 2 |
| [DB00135](https://www.drugbank.ca/drugs/DB00135) | Tyrosine | YARS | 25 | 6 | 6 | 5 | 8 | 0 | 12 | 2 |
| [DB00834](https://www.drugbank.ca/drugs/DB00834) | Mifepristone | KLK3 | 25 | 5 | 6 | 2 | 3 | 0 | 8 | 1 |
| [DB02709](https://www.drugbank.ca/drugs/DB02709) | Resveratrol | YARS | 25 | 6 | 6 | 5 | 8 | 0 | 12 | 2 |
| [DB04839](https://www.drugbank.ca/drugs/DB04839) | Cyproterone acetate | KLK3 | 25 | 5 | 6 | 2 | 3 | 0 | 8 | 1 |
| [DB00153](https://www.drugbank.ca/drugs/DB00153) | Ergocalciferol | CACNG1 | 24 | 9 | 2 | 5 | 4 | 1 | 11 | 2 |
| [DB00157](https://www.drugbank.ca/drugs/DB00157) | NADH | NDUFA11 | 24 | 3 | 5 | 3 | 5 | 1 | 9 | 1 |
| [DB00201](https://www.drugbank.ca/drugs/DB00201) | Caffeine | PRKDC | 24 | 7 | 5 | 3 | 3 | 3 | 9 | 2 |
| [DB00308](https://www.drugbank.ca/drugs/DB00308) | Ibutilide | CACNG1 | 24 | 9 | 2 | 5 | 4 | 1 | 11 | 2 |
| [DB00343](https://www.drugbank.ca/drugs/DB00343) | Diltiazem | CACNG1 | 24 | 9 | 2 | 5 | 4 | 1 | 11 | 2 |
| [DB00381](https://www.drugbank.ca/drugs/DB00381) | Amlodipine | CACNG1 | 24 | 9 | 2 | 5 | 4 | 1 | 11 | 2 |
| [DB00421](https://www.drugbank.ca/drugs/DB00421) | Spironolactone | CACNG1 | 24 | 9 | 2 | 5 | 4 | 1 | 11 | 2 |
| [DB00528](https://www.drugbank.ca/drugs/DB00528) | Lercanidipine | CACNG1 | 24 | 9 | 2 | 5 | 4 | 1 | 11 | 2 |
| [DB00653](https://www.drugbank.ca/drugs/DB00653) | Magnesium sulfate | CACNG1 | 24 | 9 | 2 | 5 | 4 | 1 | 11 | 2 |
| [DB00898](https://www.drugbank.ca/drugs/DB00898) | Ethanol | CACNG1 | 24 | 9 | 2 | 5 | 4 | 1 | 11 | 2 |
| [DB01054](https://www.drugbank.ca/drugs/DB01054) | Nitrendipine | CACNG1 | 24 | 9 | 2 | 5 | 4 | 1 | 11 | 2 |
| [DB01254](https://www.drugbank.ca/drugs/DB01254) | Dasatinib | EPHB4 | 24 | 4 | 1 | 4 | 2 | 0 | 13 | 7 |
| [DB01272](https://www.drugbank.ca/drugs/DB01272) | Alglucosidase alfa | M6PR | 24 | 3 | 3 | 2 | 3 | 1 | 6 | 5 |
| [DB04842](https://www.drugbank.ca/drugs/DB04842) | Fluspirilene | CACNG1 | 24 | 9 | 2 | 5 | 4 | 1 | 11 | 2 |
| [DB12010](https://www.drugbank.ca/drugs/DB12010) | Fostamatinib | EPHB4 | 24 | 4 | 1 | 4 | 2 | 0 | 13 | 7 |
| [DB01254](https://www.drugbank.ca/drugs/DB01254) | Dasatinib | ABL2 | 23 | 4 | 9 | 2 | 7 | 4 | 5 | 0 |
| [DB12010](https://www.drugbank.ca/drugs/DB12010) | Fostamatinib | ABL2 | 23 | 4 | 9 | 2 | 7 | 4 | 5 | 0 |
| [DB01278](https://www.drugbank.ca/drugs/DB01278) | Pramlintide | RAMP1 | 22 | 5 | 6 | 2 | 5 | 1 | 6 | 0 |
| [DB03754](https://www.drugbank.ca/drugs/DB03754) | Tromethamine | CANT1 | 22 | 3 | 1 | 1 | 4 | 0 | 15 | 0 |
| [DB11626](https://www.drugbank.ca/drugs/DB11626) | Tasonermin | TNFRSF1A | 22 | 5 | 6 | 8 | 10 | 0 | 10 | 3 |
| [DB01593](https://www.drugbank.ca/drugs/DB01593) | Zinc | CFI | 21 | 3 | 8 | 1 | 5 | 2 | 5 | 1 |
| [DB09130](https://www.drugbank.ca/drugs/DB09130) | Copper | CFI | 21 | 3 | 8 | 1 | 5 | 2 | 5 | 1 |
| [DB12010](https://www.drugbank.ca/drugs/DB12010) | Fostamatinib | CAMK1 | 21 | 4 | 1 | 1 | 3 | 0 | 14 | 2 |
| [DB14487](https://www.drugbank.ca/drugs/DB14487) | Zinc acetate | CFI | 21 | 3 | 8 | 1 | 5 | 2 | 5 | 1 |
| [DB14533](https://www.drugbank.ca/drugs/DB14533) | Zinc chloride | CFI | 21 | 3 | 8 | 1 | 5 | 2 | 5 | 1 |
| [DB00151](https://www.drugbank.ca/drugs/DB00151) | L-Cysteine | CDO1 | 20 | 2 | 0 | 2 | 0 | 1 | 5 | 7 |
| [DB00157](https://www.drugbank.ca/drugs/DB00157) | NADH | MTHFD2 | 20 | 5 | 1 | 0 | 1 | 0 | 12 | 7 |
| [DB00157](https://www.drugbank.ca/drugs/DB00157) | NADH | CDO1 | 20 | 2 | 0 | 2 | 0 | 1 | 5 | 7 |
| [DB08889](https://www.drugbank.ca/drugs/DB08889) | Carfilzomib | PSMB10 | 19 | 5 | 3 | 3 | 3 | 2 | 8 | 2 |
| [DB12010](https://www.drugbank.ca/drugs/DB12010) | Fostamatinib | MERTK | 19 | 3 | 8 | 3 | 7 | 2 | 3 | 1 |
| [DB00244](https://www.drugbank.ca/drugs/DB00244) | Mesalazine | IKBKB | 18 | 4 | 1 | 1 | 1 | 0 | 18 | 2 |
| [DB00768](https://www.drugbank.ca/drugs/DB00768) | Olopatadine | S100A1 | 18 | 2 | 3 | 1 | 1 | 1 | 9 | 3 |
| [DB00795](https://www.drugbank.ca/drugs/DB00795) | Sulfasalazine | IKBKB | 18 | 4 | 1 | 1 | 1 | 0 | 18 | 2 |
| [DB00945](https://www.drugbank.ca/drugs/DB00945) | Acetylsalicylic acid | IKBKB | 18 | 4 | 1 | 1 | 1 | 0 | 18 | 2 |
| [DB00995](https://www.drugbank.ca/drugs/DB00995) | Auranofin | IKBKB | 18 | 4 | 1 | 1 | 1 | 0 | 18 | 2 |
| [DB01169](https://www.drugbank.ca/drugs/DB01169) | Arsenic trioxide | IKBKB | 18 | 4 | 1 | 1 | 1 | 0 | 18 | 2 |
| [DB06151](https://www.drugbank.ca/drugs/DB06151) | Acetylcysteine | IKBKB | 18 | 4 | 1 | 1 | 1 | 0 | 18 | 2 |
| [DB12010](https://www.drugbank.ca/drugs/DB12010) | Fostamatinib | IKBKB | 18 | 4 | 1 | 1 | 1 | 0 | 18 | 2 |
| [DB00142](https://www.drugbank.ca/drugs/DB00142) | Glutamic Acid | SLC25A22 | 17 | 3 | 0 | 0 | 1 | 0 | 9 | 3 |
| [DB01440](https://www.drugbank.ca/drugs/DB01440) | gamma-Hydroxybutyric acid | SLC52A2 | 17 | 4 | 1 | 2 | 2 | 1 | 7 | 5 |
| [DB02709](https://www.drugbank.ca/drugs/DB02709) | Resveratrol | SLC2A1 | 17 | 2 | 2 | 2 | 4 | 2 | 4 | 5 |
| [DB11059](https://www.drugbank.ca/drugs/DB11059) | Carboxymethylcellulose | SLC2A1 | 17 | 2 | 2 | 2 | 4 | 2 | 4 | 5 |
| [DB12010](https://www.drugbank.ca/drugs/DB12010) | Fostamatinib | MKNK2 | 17 | 4 | 0 | 2 | 2 | 2 | 5 | 4 |
| [DB00157](https://www.drugbank.ca/drugs/DB00157) | NADH | HMOX1 | 16 | 2 | 3 | 1 | 4 | 4 | 4 | 0 |
| [DB00159](https://www.drugbank.ca/drugs/DB00159) | Icosapent | ACSL4 | 16 | 2 | 1 | 2 | 4 | 0 | 8 | 3 |
| [DB00412](https://www.drugbank.ca/drugs/DB00412) | Rosiglitazone | ACSL4 | 16 | 2 | 1 | 2 | 4 | 0 | 8 | 3 |
| [DB00905](https://www.drugbank.ca/drugs/DB00905) | Bimatoprost | PTGER3 | 16 | 1 | 1 | 1 | 1 | 1 | 6 | 5 |
| [DB00917](https://www.drugbank.ca/drugs/DB00917) | Dinoprostone | PTGER3 | 16 | 1 | 1 | 1 | 1 | 1 | 6 | 5 |
| [DB00929](https://www.drugbank.ca/drugs/DB00929) | Misoprostol | PTGER3 | 16 | 1 | 1 | 1 | 1 | 1 | 6 | 5 |
| [DB00945](https://www.drugbank.ca/drugs/DB00945) | Acetylsalicylic acid | CCND1 | 16 | 1 | 0 | 0 | 1 | 0 | 9 | 8 |
| [DB01169](https://www.drugbank.ca/drugs/DB01169) | Arsenic trioxide | CCND1 | 16 | 1 | 0 | 0 | 1 | 0 | 9 | 8 |
| [DB02325](https://www.drugbank.ca/drugs/DB02325) | Isopropyl alcohol | DCXR | 16 | 1 | 2 | 2 | 5 | 0 | 6 | 0 |
| [DB11113](https://www.drugbank.ca/drugs/DB11113) | Castor oil | PTGER3 | 16 | 1 | 1 | 1 | 1 | 1 | 6 | 5 |
| [DB11718](https://www.drugbank.ca/drugs/DB11718) | Encorafenib | CCND1 | 16 | 1 | 0 | 0 | 1 | 0 | 9 | 8 |
| [DB00142](https://www.drugbank.ca/drugs/DB00142) | Glutamic Acid | SLC25A18 | 15 | 6 | 1 | 2 | 3 | 1 | 5 | 2 |
| [DB00159](https://www.drugbank.ca/drugs/DB00159) | Icosapent | PTGS2 | 15 | 1 | 4 | 1 | 3 | 3 | 2 | 1 |
| [DB00162](https://www.drugbank.ca/drugs/DB00162) | Vitamin A | RDH12 | 15 | 3 | 2 | 3 | 4 | 1 | 7 | 2 |
| [DB00210](https://www.drugbank.ca/drugs/DB00210) | Adapalene | PTGS2 | 15 | 1 | 4 | 1 | 3 | 3 | 2 | 1 |
| [DB00233](https://www.drugbank.ca/drugs/DB00233) | Aminosalicylic Acid | PTGS2 | 15 | 1 | 4 | 1 | 3 | 3 | 2 | 1 |
| [DB00244](https://www.drugbank.ca/drugs/DB00244) | Mesalazine | PTGS2 | 15 | 1 | 4 | 1 | 3 | 3 | 2 | 1 |
| [DB00316](https://www.drugbank.ca/drugs/DB00316) | Acetaminophen | PTGS2 | 15 | 1 | 4 | 1 | 3 | 3 | 2 | 1 |
| [DB00328](https://www.drugbank.ca/drugs/DB00328) | Indometacin | PTGS2 | 15 | 1 | 4 | 1 | 3 | 3 | 2 | 1 |
| [DB00428](https://www.drugbank.ca/drugs/DB00428) | Streptozocin | MGEA5 | 15 | 2 | 1 | 0 | 1 | 2 | 3 | 9 |
| [DB00461](https://www.drugbank.ca/drugs/DB00461) | Nabumetone | PTGS2 | 15 | 1 | 4 | 1 | 3 | 3 | 2 | 1 |
| [DB00465](https://www.drugbank.ca/drugs/DB00465) | Ketorolac | PTGS2 | 15 | 1 | 4 | 1 | 3 | 3 | 2 | 1 |
| [DB00469](https://www.drugbank.ca/drugs/DB00469) | Tenoxicam | PTGS2 | 15 | 1 | 4 | 1 | 3 | 3 | 2 | 1 |
| [DB00480](https://www.drugbank.ca/drugs/DB00480) | Lenalidomide | PTGS2 | 15 | 1 | 4 | 1 | 3 | 3 | 2 | 1 |
| [DB00482](https://www.drugbank.ca/drugs/DB00482) | Celecoxib | PTGS2 | 15 | 1 | 4 | 1 | 3 | 3 | 2 | 1 |
| [DB00500](https://www.drugbank.ca/drugs/DB00500) | Tolmetin | PTGS2 | 15 | 1 | 4 | 1 | 3 | 3 | 2 | 1 |
| [DB00554](https://www.drugbank.ca/drugs/DB00554) | Piroxicam | PTGS2 | 15 | 1 | 4 | 1 | 3 | 3 | 2 | 1 |
| [DB00573](https://www.drugbank.ca/drugs/DB00573) | Fenoprofen | PTGS2 | 15 | 1 | 4 | 1 | 3 | 3 | 2 | 1 |
| [DB00586](https://www.drugbank.ca/drugs/DB00586) | Diclofenac | PTGS2 | 15 | 1 | 4 | 1 | 3 | 3 | 2 | 1 |
| [DB00605](https://www.drugbank.ca/drugs/DB00605) | Sulindac | PTGS2 | 15 | 1 | 4 | 1 | 3 | 3 | 2 | 1 |
| [DB00712](https://www.drugbank.ca/drugs/DB00712) | Flurbiprofen | PTGS2 | 15 | 1 | 4 | 1 | 3 | 3 | 2 | 1 |
| [DB00749](https://www.drugbank.ca/drugs/DB00749) | Etodolac | PTGS2 | 15 | 1 | 4 | 1 | 3 | 3 | 2 | 1 |
| [DB00784](https://www.drugbank.ca/drugs/DB00784) | Mefenamic acid | PTGS2 | 15 | 1 | 4 | 1 | 3 | 3 | 2 | 1 |
| [DB00788](https://www.drugbank.ca/drugs/DB00788) | Naproxen | PTGS2 | 15 | 1 | 4 | 1 | 3 | 3 | 2 | 1 |
| [DB00795](https://www.drugbank.ca/drugs/DB00795) | Sulfasalazine | PTGS2 | 15 | 1 | 4 | 1 | 3 | 3 | 2 | 1 |
| [DB00812](https://www.drugbank.ca/drugs/DB00812) | Phenylbutazone | PTGS2 | 15 | 1 | 4 | 1 | 3 | 3 | 2 | 1 |
| [DB00814](https://www.drugbank.ca/drugs/DB00814) | Meloxicam | PTGS2 | 15 | 1 | 4 | 1 | 3 | 3 | 2 | 1 |
| [DB00861](https://www.drugbank.ca/drugs/DB00861) | Diflunisal | PTGS2 | 15 | 1 | 4 | 1 | 3 | 3 | 2 | 1 |
| [DB00936](https://www.drugbank.ca/drugs/DB00936) | Salicylic acid | PTGS2 | 15 | 1 | 4 | 1 | 3 | 3 | 2 | 1 |
| [DB00939](https://www.drugbank.ca/drugs/DB00939) | Meclofenamic acid | PTGS2 | 15 | 1 | 4 | 1 | 3 | 3 | 2 | 1 |
| [DB00945](https://www.drugbank.ca/drugs/DB00945) | Acetylsalicylic acid | PTGS2 | 15 | 1 | 4 | 1 | 3 | 3 | 2 | 1 |
| [DB00963](https://www.drugbank.ca/drugs/DB00963) | Bromfenac | PTGS2 | 15 | 1 | 4 | 1 | 3 | 3 | 2 | 1 |
| [DB00991](https://www.drugbank.ca/drugs/DB00991) | Oxaprozin | PTGS2 | 15 | 1 | 4 | 1 | 3 | 3 | 2 | 1 |
| [DB01009](https://www.drugbank.ca/drugs/DB01009) | Ketoprofen | PTGS2 | 15 | 1 | 4 | 1 | 3 | 3 | 2 | 1 |
| [DB01014](https://www.drugbank.ca/drugs/DB01014) | Balsalazide | PTGS2 | 15 | 1 | 4 | 1 | 3 | 3 | 2 | 1 |
| [DB01050](https://www.drugbank.ca/drugs/DB01050) | Ibuprofen | PTGS2 | 15 | 1 | 4 | 1 | 3 | 3 | 2 | 1 |
| [DB01283](https://www.drugbank.ca/drugs/DB01283) | Lumiracoxib | PTGS2 | 15 | 1 | 4 | 1 | 3 | 3 | 2 | 1 |
| [DB01399](https://www.drugbank.ca/drugs/DB01399) | Salsalate | PTGS2 | 15 | 1 | 4 | 1 | 3 | 3 | 2 | 1 |
| [DB01401](https://www.drugbank.ca/drugs/DB01401) | Choline magnesium trisalicylate | PTGS2 | 15 | 1 | 4 | 1 | 3 | 3 | 2 | 1 |
| [DB01404](https://www.drugbank.ca/drugs/DB01404) | Ginseng | PTGS2 | 15 | 1 | 4 | 1 | 3 | 3 | 2 | 1 |
| [DB01419](https://www.drugbank.ca/drugs/DB01419) | Antrafenine | PTGS2 | 15 | 1 | 4 | 1 | 3 | 3 | 2 | 1 |
| [DB01435](https://www.drugbank.ca/drugs/DB01435) | Antipyrine | PTGS2 | 15 | 1 | 4 | 1 | 3 | 3 | 2 | 1 |
| [DB01600](https://www.drugbank.ca/drugs/DB01600) | Tiaprofenic acid | PTGS2 | 15 | 1 | 4 | 1 | 3 | 3 | 2 | 1 |
| [DB01628](https://www.drugbank.ca/drugs/DB01628) | Etoricoxib | PTGS2 | 15 | 1 | 4 | 1 | 3 | 3 | 2 | 1 |
| [DB02266](https://www.drugbank.ca/drugs/DB02266) | Flufenamic Acid | PTGS2 | 15 | 1 | 4 | 1 | 3 | 3 | 2 | 1 |
| [DB02709](https://www.drugbank.ca/drugs/DB02709) | Resveratrol | PTGS2 | 15 | 1 | 4 | 1 | 3 | 3 | 2 | 1 |
| [DB06725](https://www.drugbank.ca/drugs/DB06725) | Lornoxicam | PTGS2 | 15 | 1 | 4 | 1 | 3 | 3 | 2 | 1 |
| [DB06736](https://www.drugbank.ca/drugs/DB06736) | Aceclofenac | PTGS2 | 15 | 1 | 4 | 1 | 3 | 3 | 2 | 1 |
| [DB06802](https://www.drugbank.ca/drugs/DB06802) | Nepafenac | PTGS2 | 15 | 1 | 4 | 1 | 3 | 3 | 2 | 1 |
| [DB08439](https://www.drugbank.ca/drugs/DB08439) | Parecoxib | PTGS2 | 15 | 1 | 4 | 1 | 3 | 3 | 2 | 1 |
| [DB08910](https://www.drugbank.ca/drugs/DB08910) | Pomalidomide | PTGS2 | 15 | 1 | 4 | 1 | 3 | 3 | 2 | 1 |
| [DB09213](https://www.drugbank.ca/drugs/DB09213) | Dexibuprofen | PTGS2 | 15 | 1 | 4 | 1 | 3 | 3 | 2 | 1 |
| [DB09214](https://www.drugbank.ca/drugs/DB09214) | Dexketoprofen | PTGS2 | 15 | 1 | 4 | 1 | 3 | 3 | 2 | 1 |
| [DB09216](https://www.drugbank.ca/drugs/DB09216) | Tolfenamic Acid | PTGS2 | 15 | 1 | 4 | 1 | 3 | 3 | 2 | 1 |
| [DB11071](https://www.drugbank.ca/drugs/DB11071) | Phenyl salicylate | PTGS2 | 15 | 1 | 4 | 1 | 3 | 3 | 2 | 1 |
| [DB11079](https://www.drugbank.ca/drugs/DB11079) | Trolamine salicylate | PTGS2 | 15 | 1 | 4 | 1 | 3 | 3 | 2 | 1 |
| [DB11201](https://www.drugbank.ca/drugs/DB11201) | Menthyl salicylate | PTGS2 | 15 | 1 | 4 | 1 | 3 | 3 | 2 | 1 |
| [DB11323](https://www.drugbank.ca/drugs/DB11323) | Glycol salicylate | PTGS2 | 15 | 1 | 4 | 1 | 3 | 3 | 2 | 1 |
| [DB11327](https://www.drugbank.ca/drugs/DB11327) | Dipyrithione | PTGS2 | 15 | 1 | 4 | 1 | 3 | 3 | 2 | 1 |
| [DB13783](https://www.drugbank.ca/drugs/DB13783) | Acemetacin | PTGS2 | 15 | 1 | 4 | 1 | 3 | 3 | 2 | 1 |
| [DB13961](https://www.drugbank.ca/drugs/DB13961) | Fish oil | PTGS2 | 15 | 1 | 4 | 1 | 3 | 3 | 2 | 1 |
| [DB00806](https://www.drugbank.ca/drugs/DB00806) | Pentoxifylline | NT5E | 14 | 2 | 0 | 2 | 3 | 2 | 3 | 4 |
| [DB01016](https://www.drugbank.ca/drugs/DB01016) | Glyburide | ABCC9 | 14 | 3 | 0 | 1 | 1 | 0 | 7 | 7 |
| [DB09220](https://www.drugbank.ca/drugs/DB09220) | Nicorandil | ABCC9 | 14 | 3 | 0 | 1 | 1 | 0 | 7 | 7 |
| [DB00552](https://www.drugbank.ca/drugs/DB00552) | Pentostatin | ADA | 13 | 3 | 5 | 3 | 3 | 0 | 5 | 0 |
| [DB00975](https://www.drugbank.ca/drugs/DB00975) | Dipyridamole | ADA | 13 | 3 | 5 | 3 | 3 | 0 | 5 | 0 |
| [DB06589](https://www.drugbank.ca/drugs/DB06589) | Pazopanib | ITK | 13 | 2 | 1 | 2 | 3 | 2 | 7 | 0 |
| [DB09401](https://www.drugbank.ca/drugs/DB09401) | Isosorbide | BCL2L1 | 13 | 2 | 1 | 1 | 1 | 4 | 2 | 2 |
| [DB12010](https://www.drugbank.ca/drugs/DB12010) | Fostamatinib | ITK | 13 | 2 | 1 | 2 | 3 | 2 | 7 | 0 |
| [DB12010](https://www.drugbank.ca/drugs/DB12010) | Fostamatinib | MAPK10 | 13 | 3 | 3 | 1 | 1 | 0 | 5 | 0 |
| [DB00039](https://www.drugbank.ca/drugs/DB00039) | Palifermin | FGFR1 | 12 | 1 | 1 | 1 | 4 | 0 | 5 | 6 |
| [DB00148](https://www.drugbank.ca/drugs/DB00148) | Creatine | GAMT | 12 | 6 | 0 | 3 | 3 | 0 | 8 | 2 |
| [DB00157](https://www.drugbank.ca/drugs/DB00157) | NADH | ALDH4A1 | 12 | 0 | 2 | 2 | 4 | 1 | 7 | 0 |
| [DB00398](https://www.drugbank.ca/drugs/DB00398) | Sorafenib | FGFR1 | 12 | 1 | 1 | 1 | 4 | 0 | 5 | 6 |
| [DB00536](https://www.drugbank.ca/drugs/DB00536) | Guanidine | GAMT | 12 | 6 | 0 | 3 | 3 | 0 | 8 | 2 |
| [DB01109](https://www.drugbank.ca/drugs/DB01109) | Heparin | FGFR1 | 12 | 1 | 1 | 1 | 4 | 0 | 5 | 6 |
| [DB08896](https://www.drugbank.ca/drugs/DB08896) | Regorafenib | FGFR1 | 12 | 1 | 1 | 1 | 4 | 0 | 5 | 6 |
| [DB08901](https://www.drugbank.ca/drugs/DB08901) | Ponatinib | FGFR1 | 12 | 1 | 1 | 1 | 4 | 0 | 5 | 6 |
| [DB09078](https://www.drugbank.ca/drugs/DB09078) | Lenvatinib | FGFR1 | 12 | 1 | 1 | 1 | 4 | 0 | 5 | 6 |
| [DB09079](https://www.drugbank.ca/drugs/DB09079) | Nintedanib | FGFR1 | 12 | 1 | 1 | 1 | 4 | 0 | 5 | 6 |
| [DB12010](https://www.drugbank.ca/drugs/DB12010) | Fostamatinib | FGFR1 | 12 | 1 | 1 | 1 | 4 | 0 | 5 | 6 |
| [DB12147](https://www.drugbank.ca/drugs/DB12147) | Erdafitinib | FGFR1 | 12 | 1 | 1 | 1 | 4 | 0 | 5 | 6 |
| [DB00025](https://www.drugbank.ca/drugs/DB00025) | Antihemophilic factor, human recombinant | CANX | 11 | 4 | 2 | 4 | 3 | 0 | 8 | 2 |
| [DB00031](https://www.drugbank.ca/drugs/DB00031) | Tenecteplase | CANX | 11 | 4 | 2 | 4 | 3 | 0 | 8 | 2 |
| [DB00042](https://www.drugbank.ca/drugs/DB00042) | Botulinum Toxin Type B | SYT2 | 11 | 1 | 1 | 3 | 3 | 2 | 0 | 3 |
| [DB00231](https://www.drugbank.ca/drugs/DB00231) | Temazepam | GABRA6 | 11 | 3 | 0 | 1 | 1 | 0 | 6 | 1 |
| [DB00241](https://www.drugbank.ca/drugs/DB00241) | Butalbital | GABRA6 | 11 | 3 | 0 | 1 | 1 | 0 | 6 | 1 |
| [DB00306](https://www.drugbank.ca/drugs/DB00306) | Talbutal | GABRA6 | 11 | 3 | 0 | 1 | 1 | 0 | 6 | 1 |
| [DB00312](https://www.drugbank.ca/drugs/DB00312) | Pentobarbital | GABRA6 | 11 | 3 | 0 | 1 | 1 | 0 | 6 | 1 |
| [DB00371](https://www.drugbank.ca/drugs/DB00371) | Meprobamate | GABRA6 | 11 | 3 | 0 | 1 | 1 | 0 | 6 | 1 |
| [DB00404](https://www.drugbank.ca/drugs/DB00404) | Alprazolam | GABRA6 | 11 | 3 | 0 | 1 | 1 | 0 | 6 | 1 |
| [DB00418](https://www.drugbank.ca/drugs/DB00418) | Secobarbital | GABRA6 | 11 | 3 | 0 | 1 | 1 | 0 | 6 | 1 |
| [DB00599](https://www.drugbank.ca/drugs/DB00599) | Thiopental | GABRA6 | 11 | 3 | 0 | 1 | 1 | 0 | 6 | 1 |
| [DB00683](https://www.drugbank.ca/drugs/DB00683) | Midazolam | GABRA6 | 11 | 3 | 0 | 1 | 1 | 0 | 6 | 1 |
| [DB00690](https://www.drugbank.ca/drugs/DB00690) | Flurazepam | GABRA6 | 11 | 3 | 0 | 1 | 1 | 0 | 6 | 1 |
| [DB00794](https://www.drugbank.ca/drugs/DB00794) | Primidone | GABRA6 | 11 | 3 | 0 | 1 | 1 | 0 | 6 | 1 |
| [DB00842](https://www.drugbank.ca/drugs/DB00842) | Oxazepam | GABRA6 | 11 | 3 | 0 | 1 | 1 | 0 | 6 | 1 |
| [DB00849](https://www.drugbank.ca/drugs/DB00849) | Methylphenobarbital | GABRA6 | 11 | 3 | 0 | 1 | 1 | 0 | 6 | 1 |
| [DB00897](https://www.drugbank.ca/drugs/DB00897) | Triazolam | GABRA6 | 11 | 3 | 0 | 1 | 1 | 0 | 6 | 1 |
| [DB00898](https://www.drugbank.ca/drugs/DB00898) | Ethanol | GABRA6 | 11 | 3 | 0 | 1 | 1 | 0 | 6 | 1 |
| [DB01351](https://www.drugbank.ca/drugs/DB01351) | Amobarbital | GABRA6 | 11 | 3 | 0 | 1 | 1 | 0 | 6 | 1 |
| [DB01353](https://www.drugbank.ca/drugs/DB01353) | Butobarbital | GABRA6 | 11 | 3 | 0 | 1 | 1 | 0 | 6 | 1 |
| [DB01544](https://www.drugbank.ca/drugs/DB01544) | Flunitrazepam | GABRA6 | 11 | 3 | 0 | 1 | 1 | 0 | 6 | 1 |
| [DB01558](https://www.drugbank.ca/drugs/DB01558) | Bromazepam | GABRA6 | 11 | 3 | 0 | 1 | 1 | 0 | 6 | 1 |
| [DB01595](https://www.drugbank.ca/drugs/DB01595) | Nitrazepam | GABRA6 | 11 | 3 | 0 | 1 | 1 | 0 | 6 | 1 |
| [DB05018](https://www.drugbank.ca/drugs/DB05018) | Migalastat | GLA | 11 | 2 | 0 | 3 | 5 | 0 | 9 | 0 |
| [DB08818](https://www.drugbank.ca/drugs/DB08818) | Hyaluronic acid | CD44 | 11 | 0 | 1 | 3 | 4 | 0 | 6 | 1 |
| [DB11093](https://www.drugbank.ca/drugs/DB11093) | Calcium Citrate | CANX | 11 | 4 | 2 | 4 | 3 | 0 | 8 | 2 |
| [DB11348](https://www.drugbank.ca/drugs/DB11348) | Calcium Phosphate | CANX | 11 | 4 | 2 | 4 | 3 | 0 | 8 | 2 |
| [DB12010](https://www.drugbank.ca/drugs/DB12010) | Fostamatinib | MAST1 | 11 | 2 | 0 | 1 | 3 | 2 | 1 | 3 |
| [DB12010](https://www.drugbank.ca/drugs/DB12010) | Fostamatinib | PLK2 | 11 | 3 | 0 | 0 | 0 | 0 | 9 | 6 |
| [DB13998](https://www.drugbank.ca/drugs/DB13998) | Lonoctocog alfa | CANX | 11 | 4 | 2 | 4 | 3 | 0 | 8 | 2 |
| [DB13999](https://www.drugbank.ca/drugs/DB13999) | Moroctocog alfa | CANX | 11 | 4 | 2 | 4 | 3 | 0 | 8 | 2 |
| [DB14481](https://www.drugbank.ca/drugs/DB14481) | Calcium phosphate dihydrate | CANX | 11 | 4 | 2 | 4 | 3 | 0 | 8 | 2 |
| [DB00002](https://www.drugbank.ca/drugs/DB00002) | Cetuximab | C1QA | 10 | 2 | 1 | 1 | 2 | 2 | 3 | 2 |
| [DB00005](https://www.drugbank.ca/drugs/DB00005) | Etanercept | C1QA | 10 | 2 | 1 | 1 | 2 | 2 | 3 | 2 |
| [DB00028](https://www.drugbank.ca/drugs/DB00028) | Immune Globulin Human | C4B | 10 | 2 | 4 | 3 | 5 | 1 | 3 | 1 |
| [DB00054](https://www.drugbank.ca/drugs/DB00054) | Abciximab | C1QA | 10 | 2 | 1 | 1 | 2 | 2 | 3 | 2 |
| [DB00056](https://www.drugbank.ca/drugs/DB00056) | Gemtuzumab ozogamicin | C1QA | 10 | 2 | 1 | 1 | 2 | 2 | 3 | 2 |
| [DB00072](https://www.drugbank.ca/drugs/DB00072) | Trastuzumab | C1QA | 10 | 2 | 1 | 1 | 2 | 2 | 3 | 2 |
| [DB00074](https://www.drugbank.ca/drugs/DB00074) | Basiliximab | C1QA | 10 | 2 | 1 | 1 | 2 | 2 | 3 | 2 |
| [DB00075](https://www.drugbank.ca/drugs/DB00075) | Muromonab | C1QA | 10 | 2 | 1 | 1 | 2 | 2 | 3 | 2 |
| [DB00078](https://www.drugbank.ca/drugs/DB00078) | Ibritumomab tiuxetan | C1QA | 10 | 2 | 1 | 1 | 2 | 2 | 3 | 2 |
| [DB00081](https://www.drugbank.ca/drugs/DB00081) | Tositumomab | C1QA | 10 | 2 | 1 | 1 | 2 | 2 | 3 | 2 |
| [DB00087](https://www.drugbank.ca/drugs/DB00087) | Alemtuzumab | C1QA | 10 | 2 | 1 | 1 | 2 | 2 | 3 | 2 |
| [DB00095](https://www.drugbank.ca/drugs/DB00095) | Efalizumab | C1QA | 10 | 2 | 1 | 1 | 2 | 2 | 3 | 2 |
| [DB00108](https://www.drugbank.ca/drugs/DB00108) | Natalizumab | C1QA | 10 | 2 | 1 | 1 | 2 | 2 | 3 | 2 |
| [DB00110](https://www.drugbank.ca/drugs/DB00110) | Palivizumab | C1QA | 10 | 2 | 1 | 1 | 2 | 2 | 3 | 2 |
| [DB00112](https://www.drugbank.ca/drugs/DB00112) | Bevacizumab | C1QA | 10 | 2 | 1 | 1 | 2 | 2 | 3 | 2 |
| [DB00142](https://www.drugbank.ca/drugs/DB00142) | Glutamic Acid | GRIK5 | 10 | 0 | 2 | 2 | 5 | 1 | 2 | 1 |
| [DB00144](https://www.drugbank.ca/drugs/DB00144) | Phosphatidyl serine | PISD | 10 | 1 | 2 | 4 | 2 | 3 | 0 | 3 |
| [DB01133](https://www.drugbank.ca/drugs/DB01133) | Tiludronic acid | PTPN1 | 10 | 2 | 0 | 0 | 0 | 0 | 6 | 3 |
| [DB01593](https://www.drugbank.ca/drugs/DB01593) | Zinc | C4B | 10 | 2 | 4 | 3 | 5 | 1 | 3 | 1 |
| [DB02659](https://www.drugbank.ca/drugs/DB02659) | Cholic Acid | FECH | 10 | 1 | 2 | 1 | 3 | 2 | 3 | 1 |
| [DB02659](https://www.drugbank.ca/drugs/DB02659) | Cholic Acid | DKFZp686P18130 | 10 | 1 | 2 | 1 | 3 | 2 | 3 | 1 |
| [DB03247](https://www.drugbank.ca/drugs/DB03247) | Flavin mononucleotide | SGK1 | 10 | 2 | 2 | 1 | 4 | 0 | 4 | 0 |
| [DB09130](https://www.drugbank.ca/drugs/DB09130) | Copper | C4B | 10 | 2 | 4 | 3 | 5 | 1 | 3 | 1 |
| [DB11614](https://www.drugbank.ca/drugs/DB11614) | Rupatadine | PTAFR | 10 | 1 | 2 | 2 | 4 | 0 | 2 | 1 |
| [DB12010](https://www.drugbank.ca/drugs/DB12010) | Fostamatinib | MAP3K12 | 10 | 3 | 1 | 1 | 3 | 0 | 5 | 1 |
| [DB14487](https://www.drugbank.ca/drugs/DB14487) | Zinc acetate | C4B | 10 | 2 | 4 | 3 | 5 | 1 | 3 | 1 |
| [DB14533](https://www.drugbank.ca/drugs/DB14533) | Zinc chloride | C4B | 10 | 2 | 4 | 3 | 5 | 1 | 3 | 1 |
| [DB00157](https://www.drugbank.ca/drugs/DB00157) | NADH | HSD17B10 | 9 | 2 | 4 | 2 | 3 | 0 | 4 | 0 |
| [DB00157](https://www.drugbank.ca/drugs/DB00157) | NADH | H6PD | 9 | 4 | 1 | 0 | 1 | 0 | 5 | 2 |
| [DB00176](https://www.drugbank.ca/drugs/DB00176) | Fluvoxamine | SLC6A4 | 9 | 2 | 0 | 3 | 3 | 1 | 3 | 3 |
| [DB00182](https://www.drugbank.ca/drugs/DB00182) | Amphetamine | SLC6A4 | 9 | 2 | 0 | 3 | 3 | 1 | 3 | 3 |
| [DB00191](https://www.drugbank.ca/drugs/DB00191) | Phentermine | SLC6A4 | 9 | 2 | 0 | 3 | 3 | 1 | 3 | 3 |
| [DB00193](https://www.drugbank.ca/drugs/DB00193) | Tramadol | SLC6A4 | 9 | 2 | 0 | 3 | 3 | 1 | 3 | 3 |
| [DB00206](https://www.drugbank.ca/drugs/DB00206) | Reserpine | SLC18A1 | 9 | 1 | 0 | 2 | 2 | 2 | 0 | 4 |
| [DB00215](https://www.drugbank.ca/drugs/DB00215) | Citalopram | SLC6A4 | 9 | 2 | 0 | 3 | 3 | 1 | 3 | 3 |
| [DB00245](https://www.drugbank.ca/drugs/DB00245) | Benzatropine | SLC6A4 | 9 | 2 | 0 | 3 | 3 | 1 | 3 | 3 |
| [DB00285](https://www.drugbank.ca/drugs/DB00285) | Venlafaxine | SLC6A4 | 9 | 2 | 0 | 3 | 3 | 1 | 3 | 3 |
| [DB00289](https://www.drugbank.ca/drugs/DB00289) | Atomoxetine | SLC6A4 | 9 | 2 | 0 | 3 | 3 | 1 | 3 | 3 |
| [DB00321](https://www.drugbank.ca/drugs/DB00321) | Amitriptyline | SLC6A4 | 9 | 2 | 0 | 3 | 3 | 1 | 3 | 3 |
| [DB00344](https://www.drugbank.ca/drugs/DB00344) | Protriptyline | SLC6A4 | 9 | 2 | 0 | 3 | 3 | 1 | 3 | 3 |
| [DB00368](https://www.drugbank.ca/drugs/DB00368) | Norepinephrine | SLC18A1 | 9 | 1 | 0 | 2 | 2 | 2 | 0 | 4 |
| [DB00408](https://www.drugbank.ca/drugs/DB00408) | Loxapine | SLC6A4 | 9 | 2 | 0 | 3 | 3 | 1 | 3 | 3 |
| [DB00454](https://www.drugbank.ca/drugs/DB00454) | Meperidine | SLC6A4 | 9 | 2 | 0 | 3 | 3 | 1 | 3 | 3 |
| [DB00458](https://www.drugbank.ca/drugs/DB00458) | Imipramine | SLC6A4 | 9 | 2 | 0 | 3 | 3 | 1 | 3 | 3 |
| [DB00472](https://www.drugbank.ca/drugs/DB00472) | Fluoxetine | SLC6A4 | 9 | 2 | 0 | 3 | 3 | 1 | 3 | 3 |
| [DB00476](https://www.drugbank.ca/drugs/DB00476) | Duloxetine | SLC6A4 | 9 | 2 | 0 | 3 | 3 | 1 | 3 | 3 |
| [DB00514](https://www.drugbank.ca/drugs/DB00514) | Dextromethorphan | SLC6A4 | 9 | 2 | 0 | 3 | 3 | 1 | 3 | 3 |
| [DB00540](https://www.drugbank.ca/drugs/DB00540) | Nortriptyline | SLC6A4 | 9 | 2 | 0 | 3 | 3 | 1 | 3 | 3 |
| [DB00543](https://www.drugbank.ca/drugs/DB00543) | Amoxapine | SLC6A4 | 9 | 2 | 0 | 3 | 3 | 1 | 3 | 3 |
| [DB00579](https://www.drugbank.ca/drugs/DB00579) | Mazindol | SLC6A4 | 9 | 2 | 0 | 3 | 3 | 1 | 3 | 3 |
| [DB00656](https://www.drugbank.ca/drugs/DB00656) | Trazodone | SLC6A4 | 9 | 2 | 0 | 3 | 3 | 1 | 3 | 3 |
| [DB00661](https://www.drugbank.ca/drugs/DB00661) | Verapamil | SLC6A4 | 9 | 2 | 0 | 3 | 3 | 1 | 3 | 3 |
| [DB00715](https://www.drugbank.ca/drugs/DB00715) | Paroxetine | SLC6A4 | 9 | 2 | 0 | 3 | 3 | 1 | 3 | 3 |
| [DB00724](https://www.drugbank.ca/drugs/DB00724) | Imiquimod | TLR7 | 9 | 0 | 2 | 3 | 2 | 1 | 2 | 3 |
| [DB00726](https://www.drugbank.ca/drugs/DB00726) | Trimipramine | SLC6A4 | 9 | 2 | 0 | 3 | 3 | 1 | 3 | 3 |
| [DB00805](https://www.drugbank.ca/drugs/DB00805) | Minaprine | SLC6A4 | 9 | 2 | 0 | 3 | 3 | 1 | 3 | 3 |
| [DB00852](https://www.drugbank.ca/drugs/DB00852) | Pseudoephedrine | SLC6A4 | 9 | 2 | 0 | 3 | 3 | 1 | 3 | 3 |
| [DB00907](https://www.drugbank.ca/drugs/DB00907) | Cocaine | SLC6A4 | 9 | 2 | 0 | 3 | 3 | 1 | 3 | 3 |
| [DB00909](https://www.drugbank.ca/drugs/DB00909) | Zonisamide | SCN2B | 9 | 3 | 0 | 2 | 3 | 0 | 7 | 3 |
| [DB00988](https://www.drugbank.ca/drugs/DB00988) | Dopamine | SLC6A4 | 9 | 2 | 0 | 3 | 3 | 1 | 3 | 3 |
| [DB01104](https://www.drugbank.ca/drugs/DB01104) | Sertraline | SLC6A4 | 9 | 2 | 0 | 3 | 3 | 1 | 3 | 3 |
| [DB01114](https://www.drugbank.ca/drugs/DB01114) | Chlorphenamine | SLC6A4 | 9 | 2 | 0 | 3 | 3 | 1 | 3 | 3 |
| [DB01142](https://www.drugbank.ca/drugs/DB01142) | Doxepin | SLC6A4 | 9 | 2 | 0 | 3 | 3 | 1 | 3 | 3 |
| [DB01151](https://www.drugbank.ca/drugs/DB01151) | Desipramine | SLC6A4 | 9 | 2 | 0 | 3 | 3 | 1 | 3 | 3 |
| [DB01175](https://www.drugbank.ca/drugs/DB01175) | Escitalopram | SLC6A4 | 9 | 2 | 0 | 3 | 3 | 1 | 3 | 3 |
| [DB01242](https://www.drugbank.ca/drugs/DB01242) | Clomipramine | SLC6A4 | 9 | 2 | 0 | 3 | 3 | 1 | 3 | 3 |
| [DB01577](https://www.drugbank.ca/drugs/DB01577) | Metamfetamine | SLC6A4 | 9 | 2 | 0 | 3 | 3 | 1 | 3 | 3 |
| [DB01577](https://www.drugbank.ca/drugs/DB01577) | Metamfetamine | SLC18A1 | 9 | 1 | 0 | 2 | 2 | 2 | 0 | 4 |
| [DB01611](https://www.drugbank.ca/drugs/DB01611) | Hydroxychloroquine | TLR7 | 9 | 0 | 2 | 3 | 2 | 1 | 2 | 3 |
| [DB04896](https://www.drugbank.ca/drugs/DB04896) | Milnacipran | SLC6A4 | 9 | 2 | 0 | 3 | 3 | 1 | 3 | 3 |
| [DB06148](https://www.drugbank.ca/drugs/DB06148) | Mianserin | SLC6A4 | 9 | 2 | 0 | 3 | 3 | 1 | 3 | 3 |
| [DB06204](https://www.drugbank.ca/drugs/DB06204) | Tapentadol | SLC6A4 | 9 | 2 | 0 | 3 | 3 | 1 | 3 | 3 |
| [DB06684](https://www.drugbank.ca/drugs/DB06684) | Vilazodone | SLC6A4 | 9 | 2 | 0 | 3 | 3 | 1 | 3 | 3 |
| [DB06700](https://www.drugbank.ca/drugs/DB06700) | Desvenlafaxine | SLC6A4 | 9 | 2 | 0 | 3 | 3 | 1 | 3 | 3 |
| [DB06701](https://www.drugbank.ca/drugs/DB06701) | Dexmethylphenidate | SLC6A4 | 9 | 2 | 0 | 3 | 3 | 1 | 3 | 3 |
| [DB08918](https://www.drugbank.ca/drugs/DB08918) | Levomilnacipran | SLC6A4 | 9 | 2 | 0 | 3 | 3 | 1 | 3 | 3 |
| [DB09016](https://www.drugbank.ca/drugs/DB09016) | Butriptyline | SLC6A4 | 9 | 2 | 0 | 3 | 3 | 1 | 3 | 3 |
| [DB09068](https://www.drugbank.ca/drugs/DB09068) | Vortioxetine | SLC6A4 | 9 | 2 | 0 | 3 | 3 | 1 | 3 | 3 |
| [DB09167](https://www.drugbank.ca/drugs/DB09167) | Dosulepin | SLC6A4 | 9 | 2 | 0 | 3 | 3 | 1 | 3 | 3 |
| [DB09195](https://www.drugbank.ca/drugs/DB09195) | Lorpiprazole | SLC6A4 | 9 | 2 | 0 | 3 | 3 | 1 | 3 | 3 |
| [DB09568](https://www.drugbank.ca/drugs/DB09568) | Omega-3-carboxylic acids | HSD17B10 | 9 | 2 | 4 | 2 | 3 | 0 | 4 | 0 |
| [DB12010](https://www.drugbank.ca/drugs/DB12010) | Fostamatinib | MAPK4 | 9 | 2 | 0 | 0 | 0 | 0 | 3 | 4 |
| [DB00184](https://www.drugbank.ca/drugs/DB00184) | Nicotine | CHRNA3 | 8 | 1 | 0 | 2 | 2 | 2 | 0 | 3 |
| [DB00472](https://www.drugbank.ca/drugs/DB00472) | Fluoxetine | CHRNA3 | 8 | 1 | 0 | 2 | 2 | 2 | 0 | 3 |
| [DB00514](https://www.drugbank.ca/drugs/DB00514) | Dextromethorphan | CHRNA3 | 8 | 1 | 0 | 2 | 2 | 2 | 0 | 3 |
| [DB00674](https://www.drugbank.ca/drugs/DB00674) | Galantamine | CHRNA3 | 8 | 1 | 0 | 2 | 2 | 2 | 0 | 3 |
| [DB00898](https://www.drugbank.ca/drugs/DB00898) | Ethanol | CHRNA3 | 8 | 1 | 0 | 2 | 2 | 2 | 0 | 3 |
| [DB00915](https://www.drugbank.ca/drugs/DB00915) | Amantadine | CHRNA3 | 8 | 1 | 0 | 2 | 2 | 2 | 0 | 3 |
| [DB01090](https://www.drugbank.ca/drugs/DB01090) | Pentolinium | CHRNA3 | 8 | 1 | 0 | 2 | 2 | 2 | 0 | 3 |
| [DB01156](https://www.drugbank.ca/drugs/DB01156) | Bupropion | CHRNA3 | 8 | 1 | 0 | 2 | 2 | 2 | 0 | 3 |
| [DB01227](https://www.drugbank.ca/drugs/DB01227) | Levacetylmethadol | CHRNA3 | 8 | 1 | 0 | 2 | 2 | 2 | 0 | 3 |
| [DB01273](https://www.drugbank.ca/drugs/DB01273) | Varenicline | CHRNA3 | 8 | 1 | 0 | 2 | 2 | 2 | 0 | 3 |
| [DB02325](https://www.drugbank.ca/drugs/DB02325) | Isopropyl alcohol | PRDX6 | 8 | 1 | 0 | 0 | 1 | 0 | 4 | 2 |
| [DB09130](https://www.drugbank.ca/drugs/DB09130) | Copper | PRDX6 | 8 | 1 | 0 | 0 | 1 | 0 | 4 | 2 |
| [DB12010](https://www.drugbank.ca/drugs/DB12010) | Fostamatinib | CAMKK1 | 8 | 1 | 0 | 2 | 2 | 1 | 1 | 2 |
| [DB12010](https://www.drugbank.ca/drugs/DB12010) | Fostamatinib | PAK3 | 8 | 1 | 0 | 1 | 1 | 2 | 1 | 1 |
| [DB00091](https://www.drugbank.ca/drugs/DB00091) | Ciclosporin | PPIF | 7 | 1 | 2 | 1 | 3 | 0 | 1 | 1 |
| [DB00142](https://www.drugbank.ca/drugs/DB00142) | Glutamic Acid | AASS | 7 | 1 | 2 | 0 | 0 | 0 | 1 | 2 |
| [DB00148](https://www.drugbank.ca/drugs/DB00148) | Creatine | SLC6A8 | 7 | 1 | 1 | 1 | 1 | 0 | 3 | 0 |
| [DB00157](https://www.drugbank.ca/drugs/DB00157) | NADH | AASS | 7 | 1 | 2 | 0 | 0 | 0 | 1 | 2 |
| [DB00157](https://www.drugbank.ca/drugs/DB00157) | NADH | NDUFA4L2 | 7 | 2 | 1 | 1 | 1 | 1 | 3 | 0 |
| [DB00172](https://www.drugbank.ca/drugs/DB00172) | Proline | PPIF | 7 | 1 | 2 | 1 | 3 | 0 | 1 | 1 |
| [DB00252](https://www.drugbank.ca/drugs/DB00252) | Phenytoin | SCN3A | 7 | 0 | 3 | 0 | 2 | 0 | 0 | 1 |
| [DB00290](https://www.drugbank.ca/drugs/DB00290) | Bleomycin | LIG3 | 7 | 2 | 0 | 1 | 1 | 2 | 0 | 2 |
| [DB00562](https://www.drugbank.ca/drugs/DB00562) | Benzthiazide | CA12 | 7 | 2 | 2 | 4 | 2 | 0 | 5 | 0 |
| [DB00755](https://www.drugbank.ca/drugs/DB00755) | Tretinoin | RARRES1 | 7 | 3 | 4 | 2 | 3 | 0 | 2 | 0 |
| [DB00774](https://www.drugbank.ca/drugs/DB00774) | Hydroflumethiazide | CA12 | 7 | 2 | 2 | 4 | 2 | 0 | 5 | 0 |
| [DB00819](https://www.drugbank.ca/drugs/DB00819) | Acetazolamide | CA12 | 7 | 2 | 2 | 4 | 2 | 0 | 5 | 0 |
| [DB00909](https://www.drugbank.ca/drugs/DB00909) | Zonisamide | SCN3A | 7 | 0 | 3 | 0 | 2 | 0 | 0 | 1 |
| [DB00909](https://www.drugbank.ca/drugs/DB00909) | Zonisamide | CA12 | 7 | 2 | 2 | 4 | 2 | 0 | 5 | 0 |
| [DB02772](https://www.drugbank.ca/drugs/DB02772) | Sucrose | RTCB | 7 | 2 | 1 | 0 | 1 | 0 | 3 | 1 |
| [DB06218](https://www.drugbank.ca/drugs/DB06218) | Lacosamide | SCN3A | 7 | 0 | 3 | 0 | 2 | 0 | 0 | 1 |
| [DB00132](https://www.drugbank.ca/drugs/DB00132) | Alpha-Linolenic Acid | RXRA | 6 | 0 | 1 | 0 | 3 | 0 | 2 | 0 |
| [DB00157](https://www.drugbank.ca/drugs/DB00157) | NADH | HPGD | 6 | 1 | 0 | 1 | 1 | 2 | 0 | 1 |
| [DB00210](https://www.drugbank.ca/drugs/DB00210) | Adapalene | RXRA | 6 | 0 | 1 | 0 | 3 | 0 | 2 | 0 |
| [DB00307](https://www.drugbank.ca/drugs/DB00307) | Bexarotene | RXRA | 6 | 0 | 1 | 0 | 3 | 0 | 2 | 0 |
| [DB00412](https://www.drugbank.ca/drugs/DB00412) | Rosiglitazone | RXRA | 6 | 0 | 1 | 0 | 3 | 0 | 2 | 0 |
| [DB00459](https://www.drugbank.ca/drugs/DB00459) | Acitretin | RXRA | 6 | 0 | 1 | 0 | 3 | 0 | 2 | 0 |
| [DB00472](https://www.drugbank.ca/drugs/DB00472) | Fluoxetine | CKS1B | 6 | 2 | 2 | 2 | 2 | 0 | 2 | 0 |
| [DB00523](https://www.drugbank.ca/drugs/DB00523) | Alitretinoin | RXRA | 6 | 0 | 1 | 0 | 3 | 0 | 2 | 0 |
| [DB00749](https://www.drugbank.ca/drugs/DB00749) | Etodolac | RXRA | 6 | 0 | 1 | 0 | 3 | 0 | 2 | 0 |
| [DB00755](https://www.drugbank.ca/drugs/DB00755) | Tretinoin | RXRA | 6 | 0 | 1 | 0 | 3 | 0 | 2 | 0 |
| [DB01254](https://www.drugbank.ca/drugs/DB01254) | Dasatinib | STAT5B | 6 | 1 | 0 | 1 | 1 | 2 | 0 | 2 |
| [DB01393](https://www.drugbank.ca/drugs/DB01393) | Bezafibrate | RXRA | 6 | 0 | 1 | 0 | 3 | 0 | 2 | 0 |
| [DB01436](https://www.drugbank.ca/drugs/DB01436) | Alfacalcidol | RXRA | 6 | 0 | 1 | 0 | 3 | 0 | 2 | 0 |
| [DB03756](https://www.drugbank.ca/drugs/DB03756) | Doconexent | RXRA | 6 | 0 | 1 | 0 | 3 | 0 | 2 | 0 |
| [DB04224](https://www.drugbank.ca/drugs/DB04224) | Oleic Acid | RXRA | 6 | 0 | 1 | 0 | 3 | 0 | 2 | 0 |
| [DB12010](https://www.drugbank.ca/drugs/DB12010) | Fostamatinib | STK38L | 6 | 0 | 1 | 2 | 3 | 1 | 1 | 1 |
| [DB00144](https://www.drugbank.ca/drugs/DB00144) | Phosphatidyl serine | PTDSS2 | 5 | 1 | 1 | 0 | 2 | 0 | 0 | 1 |
| [DB00231](https://www.drugbank.ca/drugs/DB00231) | Temazepam | GABRD | 5 | 1 | 1 | 0 | 1 | 0 | 3 | 1 |
| [DB00690](https://www.drugbank.ca/drugs/DB00690) | Flurazepam | GABRD | 5 | 1 | 1 | 0 | 1 | 0 | 3 | 1 |
| [DB00829](https://www.drugbank.ca/drugs/DB00829) | Diazepam | GABRD | 5 | 1 | 1 | 0 | 1 | 0 | 3 | 1 |
| [DB00842](https://www.drugbank.ca/drugs/DB00842) | Oxazepam | GABRD | 5 | 1 | 1 | 0 | 1 | 0 | 3 | 1 |
| [DB00897](https://www.drugbank.ca/drugs/DB00897) | Triazolam | GABRD | 5 | 1 | 1 | 0 | 1 | 0 | 3 | 1 |
| [DB00898](https://www.drugbank.ca/drugs/DB00898) | Ethanol | GABRD | 5 | 1 | 1 | 0 | 1 | 0 | 3 | 1 |
| [DB01215](https://www.drugbank.ca/drugs/DB01215) | Estazolam | GABRD | 5 | 1 | 1 | 0 | 1 | 0 | 3 | 1 |
| [DB01558](https://www.drugbank.ca/drugs/DB01558) | Bromazepam | GABRD | 5 | 1 | 1 | 0 | 1 | 0 | 3 | 1 |
| [DB01559](https://www.drugbank.ca/drugs/DB01559) | Clotiazepam | GABRD | 5 | 1 | 1 | 0 | 1 | 0 | 3 | 1 |
| [DB01587](https://www.drugbank.ca/drugs/DB01587) | Ketazolam | GABRD | 5 | 1 | 1 | 0 | 1 | 0 | 3 | 1 |
| [DB01588](https://www.drugbank.ca/drugs/DB01588) | Prazepam | GABRD | 5 | 1 | 1 | 0 | 1 | 0 | 3 | 1 |
| [DB01589](https://www.drugbank.ca/drugs/DB01589) | Quazepam | GABRD | 5 | 1 | 1 | 0 | 1 | 0 | 3 | 1 |
| [DB01593](https://www.drugbank.ca/drugs/DB01593) | Zinc | APLP2 | 5 | 0 | 1 | 0 | 2 | 0 | 1 | 1 |
| [DB01595](https://www.drugbank.ca/drugs/DB01595) | Nitrazepam | GABRD | 5 | 1 | 1 | 0 | 1 | 0 | 3 | 1 |
| [DB01839](https://www.drugbank.ca/drugs/DB01839) | Propylene glycol | F13A1 | 5 | 2 | 2 | 2 | 3 | 0 | 2 | 0 |
| [DB04209](https://www.drugbank.ca/drugs/DB04209) | Dequalinium | XIAP | 5 | 2 | 0 | 1 | 1 | 0 | 4 | 0 |
| [DB08877](https://www.drugbank.ca/drugs/DB08877) | Ruxolitinib | JAK1 | 5 | 0 | 1 | 1 | 1 | 0 | 1 | 0 |
| [DB08895](https://www.drugbank.ca/drugs/DB08895) | Tofacitinib | JAK1 | 5 | 0 | 1 | 1 | 1 | 0 | 1 | 0 |
| [DB09130](https://www.drugbank.ca/drugs/DB09130) | Copper | APOH | 5 | 0 | 0 | 1 | 0 | 0 | 0 | 3 |
| [DB09462](https://www.drugbank.ca/drugs/DB09462) | Glycerin | TGFBR2 | 5 | 2 | 0 | 0 | 1 | 0 | 2 | 0 |
| [DB10770](https://www.drugbank.ca/drugs/DB10770) | Foreskin fibroblast (neonatal) | TGFBR2 | 5 | 2 | 0 | 0 | 1 | 0 | 2 | 0 |
| [DB10772](https://www.drugbank.ca/drugs/DB10772) | Foreskin keratinocyte (neonatal) | TGFBR2 | 5 | 2 | 0 | 0 | 1 | 0 | 2 | 0 |
| [DB11300](https://www.drugbank.ca/drugs/DB11300) | Thrombin | F13A1 | 5 | 2 | 2 | 2 | 3 | 0 | 2 | 0 |
| [DB11311](https://www.drugbank.ca/drugs/DB11311) | Prothrombin | F13A1 | 5 | 2 | 2 | 2 | 3 | 0 | 2 | 0 |
| [DB11571](https://www.drugbank.ca/drugs/DB11571) | Human Thrombin | F13A1 | 5 | 2 | 2 | 2 | 3 | 0 | 2 | 0 |
| [DB11572](https://www.drugbank.ca/drugs/DB11572) | Thrombin alfa | F13A1 | 5 | 2 | 2 | 2 | 3 | 0 | 2 | 0 |
| [DB11817](https://www.drugbank.ca/drugs/DB11817) | Baricitinib | JAK1 | 5 | 0 | 1 | 1 | 1 | 0 | 1 | 0 |
| [DB12010](https://www.drugbank.ca/drugs/DB12010) | Fostamatinib | JAK1 | 5 | 0 | 1 | 1 | 1 | 0 | 1 | 0 |
| [DB12010](https://www.drugbank.ca/drugs/DB12010) | Fostamatinib | TGFBR2 | 5 | 2 | 0 | 0 | 1 | 0 | 2 | 0 |
| [DB12010](https://www.drugbank.ca/drugs/DB12010) | Fostamatinib | PKN1 | 5 | 1 | 0 | 0 | 0 | 0 | 2 | 0 |
| [DB12010](https://www.drugbank.ca/drugs/DB12010) | Fostamatinib | PRKD1 | 5 | 1 | 1 | 0 | 0 | 0 | 2 | 0 |
| [DB13151](https://www.drugbank.ca/drugs/DB13151) | Anti-inhibitor coagulant complex | F13A1 | 5 | 2 | 2 | 2 | 3 | 0 | 2 | 0 |
| [DB14487](https://www.drugbank.ca/drugs/DB14487) | Zinc acetate | APLP2 | 5 | 0 | 1 | 0 | 2 | 0 | 1 | 1 |
| [DB14533](https://www.drugbank.ca/drugs/DB14533) | Zinc chloride | APLP2 | 5 | 0 | 1 | 0 | 2 | 0 | 1 | 1 |
| [DB00039](https://www.drugbank.ca/drugs/DB00039) | Palifermin | NRP1 | 4 | 1 | 0 | 0 | 0 | 2 | 0 | 0 |
| [DB00039](https://www.drugbank.ca/drugs/DB00039) | Palifermin | FGFR3 | 4 | 1 | 0 | 0 | 0 | 2 | 0 | 0 |
| [DB00075](https://www.drugbank.ca/drugs/DB00075) | Muromonab | CD3E | 4 | 0 | 0 | 1 | 1 | 2 | 0 | 1 |
| [DB00126](https://www.drugbank.ca/drugs/DB00126) | Ascorbic acid | P4HTM | 4 | 0 | 2 | 1 | 2 | 0 | 1 | 1 |
| [DB00142](https://www.drugbank.ca/drugs/DB00142) | Glutamic Acid | NAGS | 4 | 0 | 0 | 0 | 1 | 1 | 1 | 0 |
| [DB00157](https://www.drugbank.ca/drugs/DB00157) | NADH | CYP17A1 | 4 | 1 | 0 | 1 | 1 | 0 | 1 | 0 |
| [DB00281](https://www.drugbank.ca/drugs/DB00281) | Lidocaine | SCN4A | 4 | 1 | 0 | 0 | 0 | 0 | 4 | 0 |
| [DB00396](https://www.drugbank.ca/drugs/DB00396) | Progesterone | CYP17A1 | 4 | 1 | 0 | 1 | 1 | 0 | 1 | 0 |
| [DB00586](https://www.drugbank.ca/drugs/DB00586) | Diclofenac | SCN4A | 4 | 1 | 0 | 0 | 0 | 0 | 4 | 0 |
| [DB00818](https://www.drugbank.ca/drugs/DB00818) | Propofol | SCN4A | 4 | 1 | 0 | 0 | 0 | 0 | 4 | 0 |
| [DB00909](https://www.drugbank.ca/drugs/DB00909) | Zonisamide | SCN4A | 4 | 1 | 0 | 0 | 0 | 0 | 4 | 0 |
| [DB01254](https://www.drugbank.ca/drugs/DB01254) | Dasatinib | EPHA5 | 4 | 1 | 0 | 0 | 2 | 0 | 2 | 1 |
| [DB01373](https://www.drugbank.ca/drugs/DB01373) | Calcium | S100A8 | 4 | 2 | 0 | 0 | 0 | 0 | 2 | 0 |
| [DB01593](https://www.drugbank.ca/drugs/DB01593) | Zinc | ENO1 | 4 | 0 | 2 | 1 | 0 | 1 | 1 | 1 |
| [DB01593](https://www.drugbank.ca/drugs/DB01593) | Zinc | S100A8 | 4 | 2 | 0 | 0 | 0 | 0 | 2 | 0 |
| [DB04895](https://www.drugbank.ca/drugs/DB04895) | Pegaptanib | NRP1 | 4 | 1 | 0 | 0 | 0 | 2 | 0 | 0 |
| [DB05812](https://www.drugbank.ca/drugs/DB05812) | Abiraterone | CYP17A1 | 4 | 1 | 0 | 1 | 1 | 0 | 1 | 0 |
| [DB06589](https://www.drugbank.ca/drugs/DB06589) | Pazopanib | FGFR3 | 4 | 1 | 0 | 0 | 0 | 2 | 0 | 0 |
| [DB08901](https://www.drugbank.ca/drugs/DB08901) | Ponatinib | FGFR3 | 4 | 1 | 0 | 0 | 0 | 2 | 0 | 0 |
| [DB09078](https://www.drugbank.ca/drugs/DB09078) | Lenvatinib | FGFR3 | 4 | 1 | 0 | 0 | 0 | 2 | 0 | 0 |
| [DB09079](https://www.drugbank.ca/drugs/DB09079) | Nintedanib | FGFR3 | 4 | 1 | 0 | 0 | 0 | 2 | 0 | 0 |
| [DB09130](https://www.drugbank.ca/drugs/DB09130) | Copper | S100A8 | 4 | 2 | 0 | 0 | 0 | 0 | 2 | 0 |
| [DB09130](https://www.drugbank.ca/drugs/DB09130) | Copper | ENO1 | 4 | 0 | 2 | 1 | 0 | 1 | 1 | 1 |
| [DB11093](https://www.drugbank.ca/drugs/DB11093) | Calcium Citrate | CALM2 | 4 | 0 | 1 | 0 | 1 | 0 | 2 | 0 |
| [DB11094](https://www.drugbank.ca/drugs/DB11094) | Vitamin D | GC | 4 | 0 | 0 | 1 | 0 | 0 | 0 | 2 |
| [DB11300](https://www.drugbank.ca/drugs/DB11300) | Thrombin | FGB | 4 | 0 | 0 | 1 | 0 | 0 | 0 | 2 |
| [DB11311](https://www.drugbank.ca/drugs/DB11311) | Prothrombin | FGB | 4 | 0 | 0 | 1 | 0 | 0 | 0 | 2 |
| [DB11348](https://www.drugbank.ca/drugs/DB11348) | Calcium Phosphate | CALM2 | 4 | 0 | 1 | 0 | 1 | 0 | 2 | 0 |
| [DB11571](https://www.drugbank.ca/drugs/DB11571) | Human Thrombin | FGB | 4 | 0 | 0 | 1 | 0 | 0 | 0 | 2 |
| [DB11572](https://www.drugbank.ca/drugs/DB11572) | Thrombin alfa | FGB | 4 | 0 | 0 | 1 | 0 | 0 | 0 | 2 |
| [DB12010](https://www.drugbank.ca/drugs/DB12010) | Fostamatinib | EPHA5 | 4 | 1 | 0 | 0 | 2 | 0 | 2 | 1 |
| [DB12010](https://www.drugbank.ca/drugs/DB12010) | Fostamatinib | FGFR3 | 4 | 1 | 0 | 0 | 0 | 2 | 0 | 0 |
| [DB12147](https://www.drugbank.ca/drugs/DB12147) | Erdafitinib | FGFR3 | 4 | 1 | 0 | 0 | 0 | 2 | 0 | 0 |
| [DB13151](https://www.drugbank.ca/drugs/DB13151) | Anti-inhibitor coagulant complex | FGB | 4 | 0 | 0 | 1 | 0 | 0 | 0 | 2 |
| [DB13154](https://www.drugbank.ca/drugs/DB13154) | Parachlorophenol | SCN4A | 4 | 1 | 0 | 0 | 0 | 0 | 4 | 0 |
| [DB14481](https://www.drugbank.ca/drugs/DB14481) | Calcium phosphate dihydrate | CALM2 | 4 | 0 | 1 | 0 | 1 | 0 | 2 | 0 |
| [DB14487](https://www.drugbank.ca/drugs/DB14487) | Zinc acetate | ENO1 | 4 | 0 | 2 | 1 | 0 | 1 | 1 | 1 |
| [DB14487](https://www.drugbank.ca/drugs/DB14487) | Zinc acetate | S100A8 | 4 | 2 | 0 | 0 | 0 | 0 | 2 | 0 |
| [DB14533](https://www.drugbank.ca/drugs/DB14533) | Zinc chloride | ENO1 | 4 | 0 | 2 | 1 | 0 | 1 | 1 | 1 |
| [DB14533](https://www.drugbank.ca/drugs/DB14533) | Zinc chloride | S100A8 | 4 | 2 | 0 | 0 | 0 | 0 | 2 | 0 |
| [DB00004](https://www.drugbank.ca/drugs/DB00004) | Denileukin diftitox | IL2RA | 3 | 0 | 0 | 0 | 2 | 0 | 0 | 1 |
| [DB00041](https://www.drugbank.ca/drugs/DB00041) | Aldesleukin | IL2RA | 3 | 0 | 0 | 0 | 2 | 0 | 0 | 1 |
| [DB00074](https://www.drugbank.ca/drugs/DB00074) | Basiliximab | IL2RA | 3 | 0 | 0 | 0 | 2 | 0 | 0 | 1 |
| [DB00083](https://www.drugbank.ca/drugs/DB00083) | Botulinum toxin type A | RHOB | 3 | 0 | 2 | 2 | 1 | 1 | 1 | 0 |
| [DB00123](https://www.drugbank.ca/drugs/DB00123) | L-Lysine | SLC7A2 | 3 | 0 | 2 | 0 | 1 | 0 | 0 | 0 |
| [DB00129](https://www.drugbank.ca/drugs/DB00129) | Ornithine | SLC7A2 | 3 | 0 | 2 | 0 | 1 | 0 | 0 | 0 |
| [DB00157](https://www.drugbank.ca/drugs/DB00157) | NADH | LDHA | 3 | 0 | 1 | 1 | 1 | 0 | 2 | 0 |
| [DB00157](https://www.drugbank.ca/drugs/DB00157) | NADH | ALDH2 | 3 | 0 | 0 | 0 | 0 | 0 | 1 | 1 |
| [DB00157](https://www.drugbank.ca/drugs/DB00157) | NADH | NDUFS4 | 3 | 0 | 2 | 2 | 3 | 0 | 0 | 0 |
| [DB00170](https://www.drugbank.ca/drugs/DB00170) | Menadione | PROS1 | 3 | 1 | 0 | 1 | 1 | 1 | 0 | 1 |
| [DB00398](https://www.drugbank.ca/drugs/DB00398) | Sorafenib | BRAF | 3 | 1 | 0 | 0 | 0 | 0 | 2 | 0 |
| [DB00464](https://www.drugbank.ca/drugs/DB00464) | Sodium Tetradecyl Sulfate | PROS1 | 3 | 1 | 0 | 1 | 1 | 1 | 0 | 1 |
| [DB00536](https://www.drugbank.ca/drugs/DB00536) | Guanidine | ALDH2 | 3 | 0 | 0 | 0 | 0 | 0 | 1 | 1 |
| [DB00783](https://www.drugbank.ca/drugs/DB00783) | Estradiol | BECN1 | 3 | 0 | 0 | 0 | 0 | 2 | 0 | 1 |
| [DB00822](https://www.drugbank.ca/drugs/DB00822) | Disulfiram | ALDH2 | 3 | 0 | 0 | 0 | 0 | 0 | 1 | 1 |
| [DB00904](https://www.drugbank.ca/drugs/DB00904) | Ondansetron | HTR4 | 3 | 0 | 0 | 0 | 0 | 0 | 3 | 0 |
| [DB01233](https://www.drugbank.ca/drugs/DB01233) | Metoclopramide | HTR4 | 3 | 0 | 0 | 0 | 0 | 0 | 3 | 0 |
| [DB01373](https://www.drugbank.ca/drugs/DB01373) | Calcium | COMP | 3 | 1 | 2 | 0 | 0 | 0 | 1 | 0 |
| [DB02701](https://www.drugbank.ca/drugs/DB02701) | Nicotinamide | LDHA | 3 | 0 | 1 | 1 | 1 | 0 | 2 | 0 |
| [DB06372](https://www.drugbank.ca/drugs/DB06372) | Rilonacept | IL1A | 3 | 1 | 0 | 1 | 1 | 0 | 1 | 0 |
| [DB06480](https://www.drugbank.ca/drugs/DB06480) | Prucalopride | HTR4 | 3 | 0 | 0 | 0 | 0 | 0 | 3 | 0 |
| [DB08818](https://www.drugbank.ca/drugs/DB08818) | Hyaluronic acid | C1QBP | 3 | 0 | 0 | 0 | 2 | 0 | 0 | 1 |
| [DB08818](https://www.drugbank.ca/drugs/DB08818) | Hyaluronic acid | HAPLN4 | 3 | 0 | 1 | 0 | 1 | 0 | 2 | 0 |
| [DB08881](https://www.drugbank.ca/drugs/DB08881) | Vemurafenib | BRAF | 3 | 1 | 0 | 0 | 0 | 0 | 2 | 0 |
| [DB08896](https://www.drugbank.ca/drugs/DB08896) | Regorafenib | BRAF | 3 | 1 | 0 | 0 | 0 | 0 | 2 | 0 |
| [DB08912](https://www.drugbank.ca/drugs/DB08912) | Dabrafenib | BRAF | 3 | 1 | 0 | 0 | 0 | 0 | 2 | 0 |
| [DB09118](https://www.drugbank.ca/drugs/DB09118) | Stiripentol | LDHA | 3 | 0 | 1 | 1 | 1 | 0 | 2 | 0 |
| [DB09130](https://www.drugbank.ca/drugs/DB09130) | Copper | LDHA | 3 | 0 | 1 | 1 | 1 | 0 | 2 | 0 |
| [DB09130](https://www.drugbank.ca/drugs/DB09130) | Copper | C1QBP | 3 | 0 | 0 | 0 | 2 | 0 | 0 | 1 |
| [DB09332](https://www.drugbank.ca/drugs/DB09332) | Kappadione | PROS1 | 3 | 1 | 0 | 1 | 1 | 1 | 0 | 1 |
| [DB09462](https://www.drugbank.ca/drugs/DB09462) | Glycerin | ARF1 | 3 | 0 | 1 | 0 | 0 | 0 | 1 | 0 |
| [DB11300](https://www.drugbank.ca/drugs/DB11300) | Thrombin | F2RL3 | 3 | 0 | 1 | 2 | 2 | 0 | 0 | 0 |
| [DB11718](https://www.drugbank.ca/drugs/DB11718) | Encorafenib | BRAF | 3 | 1 | 0 | 0 | 0 | 0 | 2 | 0 |
| [DB12010](https://www.drugbank.ca/drugs/DB12010) | Fostamatinib | ACVR1B | 3 | 0 | 2 | 0 | 1 | 0 | 0 | 0 |
| [DB12010](https://www.drugbank.ca/drugs/DB12010) | Fostamatinib | BMPR1B | 3 | 2 | 0 | 0 | 0 | 0 | 1 | 0 |
| [DB12010](https://www.drugbank.ca/drugs/DB12010) | Fostamatinib | BRAF | 3 | 1 | 0 | 0 | 0 | 0 | 2 | 0 |
| [DB12010](https://www.drugbank.ca/drugs/DB12010) | Fostamatinib | MAPK13 | 3 | 0 | 2 | 0 | 1 | 0 | 0 | 0 |
| [DB12010](https://www.drugbank.ca/drugs/DB12010) | Fostamatinib | RIPK1 | 3 | 1 | 0 | 0 | 0 | 0 | 1 | 1 |
| [DB13952](https://www.drugbank.ca/drugs/DB13952) | Estradiol acetate | BECN1 | 3 | 0 | 0 | 0 | 0 | 2 | 0 | 1 |
| [DB13953](https://www.drugbank.ca/drugs/DB13953) | Estradiol benzoate | BECN1 | 3 | 0 | 0 | 0 | 0 | 2 | 0 | 1 |
| [DB13954](https://www.drugbank.ca/drugs/DB13954) | Estradiol cypionate | BECN1 | 3 | 0 | 0 | 0 | 0 | 2 | 0 | 1 |
| [DB13955](https://www.drugbank.ca/drugs/DB13955) | Estradiol dienanthate | BECN1 | 3 | 0 | 0 | 0 | 0 | 2 | 0 | 1 |
| [DB13956](https://www.drugbank.ca/drugs/DB13956) | Estradiol valerate | BECN1 | 3 | 0 | 0 | 0 | 0 | 2 | 0 | 1 |
| [DB00025](https://www.drugbank.ca/drugs/DB00025) | Antihemophilic factor, human recombinant | PHYH | 2 | 0 | 1 | 0 | 0 | 0 | 1 | 0 |
| [DB00030](https://www.drugbank.ca/drugs/DB00030) | Insulin Human | RB1 | 2 | 1 | 0 | 0 | 0 | 0 | 1 | 0 |
| [DB00071](https://www.drugbank.ca/drugs/DB00071) | Insulin Pork | RB1 | 2 | 1 | 0 | 0 | 0 | 0 | 1 | 0 |
| [DB00126](https://www.drugbank.ca/drugs/DB00126) | Ascorbic acid | PHYH | 2 | 0 | 1 | 0 | 0 | 0 | 1 | 0 |
| [DB00129](https://www.drugbank.ca/drugs/DB00129) | Ornithine | OAZ3 | 2 | 0 | 1 | 0 | 0 | 0 | 1 | 0 |
| [DB00131](https://www.drugbank.ca/drugs/DB00131) | Adenosine phosphate | PRKAB2 | 2 | 0 | 0 | 0 | 0 | 0 | 0 | 1 |
| [DB00132](https://www.drugbank.ca/drugs/DB00132) | Alpha-Linolenic Acid | ELOVL4 | 2 | 0 | 0 | 0 | 0 | 2 | 0 | 0 |
| [DB00136](https://www.drugbank.ca/drugs/DB00136) | Calcitriol | VDR | 2 | 0 | 1 | 1 | 1 | 0 | 0 | 0 |
| [DB00146](https://www.drugbank.ca/drugs/DB00146) | Calcifediol | VDR | 2 | 0 | 1 | 1 | 1 | 0 | 0 | 0 |
| [DB00151](https://www.drugbank.ca/drugs/DB00151) | L-Cysteine | SLC19A3 | 2 | 0 | 1 | 0 | 0 | 0 | 1 | 0 |
| [DB00153](https://www.drugbank.ca/drugs/DB00153) | Ergocalciferol | VDR | 2 | 0 | 1 | 1 | 1 | 0 | 0 | 0 |
| [DB00157](https://www.drugbank.ca/drugs/DB00157) | NADH | NDUFA3 | 2 | 0 | 0 | 0 | 0 | 0 | 0 | 2 |
| [DB00169](https://www.drugbank.ca/drugs/DB00169) | Cholecalciferol | VDR | 2 | 0 | 1 | 1 | 1 | 0 | 0 | 0 |
| [DB00200](https://www.drugbank.ca/drugs/DB00200) | Hydroxocobalamin | CUBN | 2 | 0 | 1 | 1 | 0 | 0 | 2 | 0 |
| [DB00279](https://www.drugbank.ca/drugs/DB00279) | Liothyronine | THRB | 2 | 0 | 0 | 0 | 0 | 0 | 1 | 0 |
| [DB00390](https://www.drugbank.ca/drugs/DB00390) | Digoxin | ATP1A1 | 2 | 0 | 1 | 0 | 0 | 0 | 1 | 0 |
| [DB00451](https://www.drugbank.ca/drugs/DB00451) | Levothyroxine | THRB | 2 | 0 | 0 | 0 | 0 | 0 | 1 | 0 |
| [DB00509](https://www.drugbank.ca/drugs/DB00509) | Dextrothyroxine | THRB | 2 | 0 | 0 | 0 | 0 | 0 | 1 | 0 |
| [DB00511](https://www.drugbank.ca/drugs/DB00511) | Acetyldigitoxin | ATP1A1 | 2 | 0 | 1 | 0 | 0 | 0 | 1 | 0 |
| [DB00697](https://www.drugbank.ca/drugs/DB00697) | Tizanidine | NISCH | 2 | 0 | 0 | 0 | 1 | 0 | 0 | 0 |
| [DB00774](https://www.drugbank.ca/drugs/DB00774) | Hydroflumethiazide | ATP1A1 | 2 | 0 | 1 | 0 | 0 | 0 | 1 | 0 |
| [DB00903](https://www.drugbank.ca/drugs/DB00903) | Etacrynic acid | ATP1A1 | 2 | 0 | 1 | 0 | 0 | 0 | 1 | 0 |
| [DB00910](https://www.drugbank.ca/drugs/DB00910) | Paricalcitol | VDR | 2 | 0 | 1 | 1 | 1 | 0 | 0 | 0 |
| [DB00975](https://www.drugbank.ca/drugs/DB00975) | Dipyridamole | RCAN1 | 2 | 1 | 1 | 0 | 0 | 0 | 1 | 0 |
| [DB01021](https://www.drugbank.ca/drugs/DB01021) | Trichlormethiazide | ATP1A1 | 2 | 0 | 1 | 0 | 0 | 0 | 1 | 0 |
| [DB01070](https://www.drugbank.ca/drugs/DB01070) | Dihydrotachysterol | VDR | 2 | 0 | 1 | 1 | 1 | 0 | 0 | 0 |
| [DB01078](https://www.drugbank.ca/drugs/DB01078) | Deslanoside | ATP1A1 | 2 | 0 | 1 | 0 | 0 | 0 | 1 | 0 |
| [DB01092](https://www.drugbank.ca/drugs/DB01092) | Ouabain | ATP1A1 | 2 | 0 | 1 | 0 | 0 | 0 | 1 | 0 |
| [DB01106](https://www.drugbank.ca/drugs/DB01106) | Levocabastine | NTSR2 | 2 | 1 | 0 | 0 | 0 | 0 | 0 | 1 |
| [DB01110](https://www.drugbank.ca/drugs/DB01110) | Miconazole | KCNMB1 | 2 | 1 | 0 | 0 | 0 | 0 | 2 | 0 |
| [DB01118](https://www.drugbank.ca/drugs/DB01118) | Amiodarone | THRB | 2 | 0 | 0 | 0 | 0 | 0 | 1 | 0 |
| [DB01119](https://www.drugbank.ca/drugs/DB01119) | Diazoxide | ATP1A1 | 2 | 0 | 1 | 0 | 0 | 0 | 1 | 0 |
| [DB01158](https://www.drugbank.ca/drugs/DB01158) | Bretylium | ATP1A1 | 2 | 0 | 1 | 0 | 0 | 0 | 1 | 0 |
| [DB01188](https://www.drugbank.ca/drugs/DB01188) | Ciclopirox | ATP1A1 | 2 | 0 | 1 | 0 | 0 | 0 | 1 | 0 |
| [DB01345](https://www.drugbank.ca/drugs/DB01345) | Potassium cation | ATP1A1 | 2 | 0 | 1 | 0 | 0 | 0 | 1 | 0 |
| [DB01370](https://www.drugbank.ca/drugs/DB01370) | Aluminium | ATP1A1 | 2 | 0 | 1 | 0 | 0 | 0 | 1 | 0 |
| [DB01378](https://www.drugbank.ca/drugs/DB01378) | Magnesium cation | ATP1A1 | 2 | 0 | 1 | 0 | 0 | 0 | 1 | 0 |
| [DB01396](https://www.drugbank.ca/drugs/DB01396) | Digitoxin | ATP1A1 | 2 | 0 | 1 | 0 | 0 | 0 | 1 | 0 |
| [DB01430](https://www.drugbank.ca/drugs/DB01430) | Almitrine | ATP1A1 | 2 | 0 | 1 | 0 | 0 | 0 | 1 | 0 |
| [DB01436](https://www.drugbank.ca/drugs/DB01436) | Alfacalcidol | VDR | 2 | 0 | 1 | 1 | 1 | 0 | 0 | 0 |
| [DB01583](https://www.drugbank.ca/drugs/DB01583) | Liotrix | THRB | 2 | 0 | 0 | 0 | 0 | 0 | 1 | 0 |
| [DB01593](https://www.drugbank.ca/drugs/DB01593) | Zinc | EEF1A1 | 2 | 1 | 0 | 0 | 0 | 1 | 1 | 0 |
| [DB02300](https://www.drugbank.ca/drugs/DB02300) | Calcipotriol | VDR | 2 | 0 | 1 | 1 | 1 | 0 | 0 | 0 |
| [DB02659](https://www.drugbank.ca/drugs/DB02659) | Cholic Acid | COX6A2 | 2 | 0 | 1 | 1 | 0 | 0 | 1 | 1 |
| [DB03147](https://www.drugbank.ca/drugs/DB03147) | Flavin adenine dinucleotide | FDXR | 2 | 1 | 0 | 0 | 0 | 0 | 0 | 1 |
| [DB04540](https://www.drugbank.ca/drugs/DB04540) | Cholesterol | VDR | 2 | 0 | 1 | 1 | 1 | 0 | 0 | 0 |
| [DB06410](https://www.drugbank.ca/drugs/DB06410) | Doxercalciferol | VDR | 2 | 0 | 1 | 1 | 1 | 0 | 0 | 0 |
| [DB09130](https://www.drugbank.ca/drugs/DB09130) | Copper | EEF1A1 | 2 | 1 | 0 | 0 | 0 | 1 | 1 | 0 |
| [DB09130](https://www.drugbank.ca/drugs/DB09130) | Copper | SERPING1 | 2 | 0 | 0 | 0 | 2 | 0 | 0 | 0 |
| [DB09242](https://www.drugbank.ca/drugs/DB09242) | Moxonidine | NISCH | 2 | 0 | 0 | 0 | 1 | 0 | 0 | 0 |
| [DB09568](https://www.drugbank.ca/drugs/DB09568) | Omega-3-carboxylic acids | ELOVL4 | 2 | 0 | 0 | 0 | 0 | 2 | 0 | 0 |
| [DB11094](https://www.drugbank.ca/drugs/DB11094) | Vitamin D | VDR | 2 | 0 | 1 | 1 | 1 | 0 | 0 | 0 |
| [DB11672](https://www.drugbank.ca/drugs/DB11672) | Curcumin | VDR | 2 | 0 | 1 | 1 | 1 | 0 | 0 | 0 |
| [DB12010](https://www.drugbank.ca/drugs/DB12010) | Fostamatinib | CASK | 2 | 1 | 0 | 0 | 0 | 0 | 0 | 1 |
| [DB12010](https://www.drugbank.ca/drugs/DB12010) | Fostamatinib | EIF2AK1 | 2 | 0 | 0 | 0 | 0 | 0 | 0 | 1 |
| [DB12010](https://www.drugbank.ca/drugs/DB12010) | Fostamatinib | EPHA8 | 2 | 1 | 0 | 0 | 0 | 0 | 0 | 0 |
| [DB12010](https://www.drugbank.ca/drugs/DB12010) | Fostamatinib | LIMK2 | 2 | 0 | 1 | 1 | 1 | 0 | 1 | 0 |
| [DB12010](https://www.drugbank.ca/drugs/DB12010) | Fostamatinib | MAP4K5 | 2 | 0 | 0 | 0 | 0 | 0 | 0 | 2 |
| [DB12010](https://www.drugbank.ca/drugs/DB12010) | Fostamatinib | PRKCI | 2 | 1 | 0 | 0 | 0 | 0 | 0 | 1 |
| [DB13996](https://www.drugbank.ca/drugs/DB13996) | Magnesium acetate | ATP1A1 | 2 | 0 | 1 | 0 | 0 | 0 | 1 | 0 |
| [DB13998](https://www.drugbank.ca/drugs/DB13998) | Lonoctocog alfa | PHYH | 2 | 0 | 1 | 0 | 0 | 0 | 1 | 0 |
| [DB13999](https://www.drugbank.ca/drugs/DB13999) | Moroctocog alfa | PHYH | 2 | 0 | 1 | 0 | 0 | 0 | 1 | 0 |
| [DB14487](https://www.drugbank.ca/drugs/DB14487) | Zinc acetate | EEF1A1 | 2 | 1 | 0 | 0 | 0 | 1 | 1 | 0 |
| [DB14498](https://www.drugbank.ca/drugs/DB14498) | Potassium acetate | ATP1A1 | 2 | 0 | 1 | 0 | 0 | 0 | 1 | 0 |
| [DB14499](https://www.drugbank.ca/drugs/DB14499) | Potassium sulfate | ATP1A1 | 2 | 0 | 1 | 0 | 0 | 0 | 1 | 0 |
| [DB14517](https://www.drugbank.ca/drugs/DB14517) | Aluminium phosphate | ATP1A1 | 2 | 0 | 1 | 0 | 0 | 0 | 1 | 0 |
| [DB14518](https://www.drugbank.ca/drugs/DB14518) | Aluminum acetate | ATP1A1 | 2 | 0 | 1 | 0 | 0 | 0 | 1 | 0 |
| [DB14533](https://www.drugbank.ca/drugs/DB14533) | Zinc chloride | EEF1A1 | 2 | 1 | 0 | 0 | 0 | 1 | 1 | 0 |
| [DB00119](https://www.drugbank.ca/drugs/DB00119) | Pyruvic acid | SLC16A7 | 1 | 0 | 0 | 0 | 0 | 0 | 1 | 0 |
| [DB00125](https://www.drugbank.ca/drugs/DB00125) | L-Arginine | ASL | 1 | 0 | 0 | 0 | 0 | 0 | 0 | 1 |
| [DB00128](https://www.drugbank.ca/drugs/DB00128) | Aspartic acid | ASNS | 1 | 0 | 0 | 0 | 1 | 0 | 0 | 0 |
| [DB00141](https://www.drugbank.ca/drugs/DB00141) | N-Acetylglucosamine | B4GALT2 | 1 | 0 | 0 | 0 | 0 | 0 | 0 | 0 |
| [DB00142](https://www.drugbank.ca/drugs/DB00142) | Glutamic Acid | ASNS | 1 | 0 | 0 | 0 | 1 | 0 | 0 | 0 |
| [DB00145](https://www.drugbank.ca/drugs/DB00145) | Glycine | BAAT | 1 | 0 | 0 | 0 | 0 | 0 | 0 | 1 |
| [DB00157](https://www.drugbank.ca/drugs/DB00157) | NADH | NQO2 | 1 | 0 | 0 | 0 | 0 | 0 | 0 | 1 |
| [DB00159](https://www.drugbank.ca/drugs/DB00159) | Icosapent | ACSL3 | 1 | 1 | 0 | 0 | 0 | 0 | 0 | 0 |
| [DB00162](https://www.drugbank.ca/drugs/DB00162) | Vitamin A | LRAT | 1 | 0 | 0 | 0 | 0 | 0 | 0 | 1 |
| [DB00163](https://www.drugbank.ca/drugs/DB00163) | Vitamin E | PPP2CA | 1 | 0 | 0 | 0 | 0 | 0 | 1 | 0 |
| [DB00170](https://www.drugbank.ca/drugs/DB00170) | Menadione | NQO2 | 1 | 0 | 0 | 0 | 0 | 0 | 0 | 1 |
| [DB00172](https://www.drugbank.ca/drugs/DB00172) | Proline | PPIC | 1 | 0 | 1 | 0 | 0 | 0 | 0 | 0 |
| [DB00174](https://www.drugbank.ca/drugs/DB00174) | Asparagine | ASNS | 1 | 0 | 0 | 0 | 1 | 0 | 0 | 0 |
| [DB00184](https://www.drugbank.ca/drugs/DB00184) | Nicotine | CHRNA9 | 1 | 0 | 0 | 0 | 0 | 0 | 0 | 0 |
| [DB00184](https://www.drugbank.ca/drugs/DB00184) | Nicotine | CHRNB3 | 1 | 0 | 0 | 0 | 0 | 0 | 0 | 0 |
| [DB00206](https://www.drugbank.ca/drugs/DB00206) | Reserpine | BIRC5 | 1 | 0 | 0 | 0 | 0 | 0 | 0 | 1 |
| [DB00337](https://www.drugbank.ca/drugs/DB00337) | Pimecrolimus | MTOR | 1 | 1 | 0 | 0 | 0 | 0 | 1 | 0 |
| [DB00674](https://www.drugbank.ca/drugs/DB00674) | Galantamine | CHRNA9 | 1 | 0 | 0 | 0 | 0 | 0 | 0 | 0 |
| [DB00674](https://www.drugbank.ca/drugs/DB00674) | Galantamine | CHRNB3 | 1 | 0 | 0 | 0 | 0 | 0 | 0 | 0 |
| [DB00755](https://www.drugbank.ca/drugs/DB00755) | Tretinoin | GPRC5A | 1 | 0 | 0 | 0 | 0 | 0 | 0 | 1 |
| [DB00762](https://www.drugbank.ca/drugs/DB00762) | Irinotecan | TOP1 | 1 | 0 | 0 | 0 | 0 | 0 | 0 | 1 |
| [DB00877](https://www.drugbank.ca/drugs/DB00877) | Sirolimus | MTOR | 1 | 1 | 0 | 0 | 0 | 0 | 1 | 0 |
| [DB00898](https://www.drugbank.ca/drugs/DB00898) | Ethanol | CHRNA9 | 1 | 0 | 0 | 0 | 0 | 0 | 0 | 0 |
| [DB00898](https://www.drugbank.ca/drugs/DB00898) | Ethanol | CHRNB3 | 1 | 0 | 0 | 0 | 0 | 0 | 0 | 0 |
| [DB00945](https://www.drugbank.ca/drugs/DB00945) | Acetylsalicylic acid | RPS6KA3 | 1 | 0 | 0 | 0 | 0 | 0 | 1 | 0 |
| [DB01030](https://www.drugbank.ca/drugs/DB01030) | Topotecan | TOP1 | 1 | 0 | 0 | 0 | 0 | 0 | 0 | 1 |
| [DB01065](https://www.drugbank.ca/drugs/DB01065) | Melatonin | NQO2 | 1 | 0 | 0 | 0 | 0 | 0 | 0 | 1 |
| [DB01076](https://www.drugbank.ca/drugs/DB01076) | Atorvastatin | DPP4 | 1 | 0 | 0 | 0 | 1 | 0 | 0 | 0 |
| [DB01087](https://www.drugbank.ca/drugs/DB01087) | Primaquine | NQO2 | 1 | 0 | 0 | 0 | 0 | 0 | 0 | 1 |
| [DB01197](https://www.drugbank.ca/drugs/DB01197) | Captopril | MMP2 | 1 | 0 | 0 | 0 | 0 | 0 | 0 | 0 |
| [DB01254](https://www.drugbank.ca/drugs/DB01254) | Dasatinib | BTK | 1 | 0 | 0 | 0 | 1 | 0 | 0 | 0 |
| [DB01254](https://www.drugbank.ca/drugs/DB01254) | Dasatinib | HSPA8 | 1 | 1 | 0 | 0 | 0 | 0 | 1 | 0 |
| [DB01261](https://www.drugbank.ca/drugs/DB01261) | Sitagliptin | DPP4 | 1 | 0 | 0 | 0 | 1 | 0 | 0 | 0 |
| [DB01278](https://www.drugbank.ca/drugs/DB01278) | Pramlintide | RAMP2 | 1 | 0 | 0 | 0 | 0 | 0 | 1 | 0 |
| [DB01370](https://www.drugbank.ca/drugs/DB01370) | Aluminium | KLK1 | 1 | 0 | 0 | 0 | 0 | 0 | 0 | 0 |
| [DB01590](https://www.drugbank.ca/drugs/DB01590) | Everolimus | MTOR | 1 | 1 | 0 | 0 | 0 | 0 | 1 | 0 |
| [DB01592](https://www.drugbank.ca/drugs/DB01592) | Iron | FEN1 | 1 | 1 | 0 | 0 | 0 | 0 | 1 | 0 |
| [DB01593](https://www.drugbank.ca/drugs/DB01593) | Zinc | APOA2 | 1 | 0 | 0 | 0 | 0 | 0 | 0 | 1 |
| [DB01708](https://www.drugbank.ca/drugs/DB01708) | Prasterone | SULT2B1 | 1 | 1 | 0 | 0 | 0 | 0 | 1 | 0 |
| [DB02709](https://www.drugbank.ca/drugs/DB02709) | Resveratrol | NQO2 | 1 | 0 | 0 | 0 | 0 | 0 | 0 | 1 |
| [DB02709](https://www.drugbank.ca/drugs/DB02709) | Resveratrol | PI4K2B | 1 | 0 | 0 | 0 | 0 | 0 | 0 | 0 |
| [DB02709](https://www.drugbank.ca/drugs/DB02709) | Resveratrol | KHSRP | 1 | 1 | 0 | 0 | 0 | 0 | 1 | 0 |
| [DB02789](https://www.drugbank.ca/drugs/DB02789) | Pregnenolone | SULT2B1 | 1 | 1 | 0 | 0 | 0 | 0 | 1 | 0 |
| [DB03147](https://www.drugbank.ca/drugs/DB03147) | Flavin adenine dinucleotide | NQO2 | 1 | 0 | 0 | 0 | 0 | 0 | 0 | 1 |
| [DB04115](https://www.drugbank.ca/drugs/DB04115) | Berberine | BIRC5 | 1 | 0 | 0 | 0 | 0 | 0 | 0 | 1 |
| [DB04876](https://www.drugbank.ca/drugs/DB04876) | Vildagliptin | DPP4 | 1 | 0 | 0 | 0 | 1 | 0 | 0 | 0 |
| [DB05630](https://www.drugbank.ca/drugs/DB05630) | Sodium stibogluconate | TOP1 | 1 | 0 | 0 | 0 | 0 | 0 | 0 | 1 |
| [DB06203](https://www.drugbank.ca/drugs/DB06203) | Alogliptin | DPP4 | 1 | 0 | 0 | 0 | 1 | 0 | 0 | 0 |
| [DB06287](https://www.drugbank.ca/drugs/DB06287) | Temsirolimus | MTOR | 1 | 1 | 0 | 0 | 0 | 0 | 1 | 0 |
| [DB06335](https://www.drugbank.ca/drugs/DB06335) | Saxagliptin | DPP4 | 1 | 0 | 0 | 0 | 1 | 0 | 0 | 0 |
| [DB07776](https://www.drugbank.ca/drugs/DB07776) | Flavone | CYP1B1 | 1 | 0 | 0 | 1 | 1 | 0 | 0 | 0 |
| [DB08882](https://www.drugbank.ca/drugs/DB08882) | Linagliptin | DPP4 | 1 | 0 | 0 | 0 | 1 | 0 | 0 | 0 |
| [DB08885](https://www.drugbank.ca/drugs/DB08885) | Aflibercept | PGF | 1 | 0 | 1 | 1 | 0 | 0 | 1 | 0 |
| [DB08895](https://www.drugbank.ca/drugs/DB08895) | Tofacitinib | JAK3 | 1 | 0 | 0 | 0 | 0 | 0 | 1 | 0 |
| [DB08916](https://www.drugbank.ca/drugs/DB08916) | Afatinib | ERBB4 | 1 | 0 | 1 | 0 | 0 | 0 | 0 | 0 |
| [DB09053](https://www.drugbank.ca/drugs/DB09053) | Ibrutinib | BTK | 1 | 0 | 0 | 0 | 1 | 0 | 0 | 0 |
| [DB09069](https://www.drugbank.ca/drugs/DB09069) | Trimetazidine | ACAA1 | 1 | 0 | 0 | 0 | 0 | 0 | 1 | 0 |
| [DB09105](https://www.drugbank.ca/drugs/DB09105) | Asfotase alfa | S1PR1 | 1 | 1 | 0 | 0 | 0 | 0 | 1 | 0 |
| [DB09130](https://www.drugbank.ca/drugs/DB09130) | Copper | HNRNPH1 | 1 | 0 | 0 | 0 | 0 | 1 | 0 | 0 |
| [DB09130](https://www.drugbank.ca/drugs/DB09130) | Copper | HSPA8 | 1 | 1 | 0 | 0 | 0 | 0 | 1 | 0 |
| [DB09130](https://www.drugbank.ca/drugs/DB09130) | Copper | HDGF | 1 | 0 | 0 | 0 | 0 | 0 | 0 | 1 |
| [DB09130](https://www.drugbank.ca/drugs/DB09130) | Copper | CLIC1 | 1 | 0 | 0 | 0 | 0 | 0 | 1 | 0 |
| [DB09130](https://www.drugbank.ca/drugs/DB09130) | Copper | B2M | 1 | 0 | 0 | 0 | 0 | 0 | 0 | 0 |
| [DB09130](https://www.drugbank.ca/drugs/DB09130) | Copper | APOA2 | 1 | 0 | 0 | 0 | 0 | 0 | 0 | 1 |
| [DB11254](https://www.drugbank.ca/drugs/DB11254) | Hexylresorcinol | TOP1 | 1 | 0 | 0 | 0 | 0 | 0 | 0 | 1 |
| [DB11703](https://www.drugbank.ca/drugs/DB11703) | Acalabrutinib | BTK | 1 | 0 | 0 | 0 | 1 | 0 | 0 | 0 |
| [DB11817](https://www.drugbank.ca/drugs/DB11817) | Baricitinib | JAK3 | 1 | 0 | 0 | 0 | 0 | 0 | 1 | 0 |
| [DB12010](https://www.drugbank.ca/drugs/DB12010) | Fostamatinib | TAOK1 | 1 | 0 | 0 | 0 | 0 | 0 | 1 | 0 |
| [DB12010](https://www.drugbank.ca/drugs/DB12010) | Fostamatinib | BTK | 1 | 0 | 0 | 0 | 1 | 0 | 0 | 0 |
| [DB12010](https://www.drugbank.ca/drugs/DB12010) | Fostamatinib | CAMK1G | 1 | 0 | 0 | 0 | 0 | 0 | 1 | 0 |
| [DB12010](https://www.drugbank.ca/drugs/DB12010) | Fostamatinib | EPHB2 | 1 | 0 | 0 | 0 | 0 | 0 | 1 | 0 |
| [DB12010](https://www.drugbank.ca/drugs/DB12010) | Fostamatinib | EPHB6 | 1 | 1 | 0 | 0 | 0 | 0 | 1 | 0 |
| [DB12010](https://www.drugbank.ca/drugs/DB12010) | Fostamatinib | ERBB4 | 1 | 0 | 1 | 0 | 0 | 0 | 0 | 0 |
| [DB12010](https://www.drugbank.ca/drugs/DB12010) | Fostamatinib | MTOR | 1 | 1 | 0 | 0 | 0 | 0 | 1 | 0 |
| [DB12010](https://www.drugbank.ca/drugs/DB12010) | Fostamatinib | JAK3 | 1 | 0 | 0 | 0 | 0 | 0 | 1 | 0 |
| [DB12010](https://www.drugbank.ca/drugs/DB12010) | Fostamatinib | PLK4 | 1 | 1 | 0 | 0 | 0 | 0 | 1 | 0 |
| [DB12010](https://www.drugbank.ca/drugs/DB12010) | Fostamatinib | RPS6KA3 | 1 | 0 | 0 | 0 | 0 | 0 | 1 | 0 |
| [DB12267](https://www.drugbank.ca/drugs/DB12267) | Brigatinib | ERBB4 | 1 | 0 | 1 | 0 | 0 | 0 | 0 | 0 |
| [DB12371](https://www.drugbank.ca/drugs/DB12371) | Siponimod | S1PR1 | 1 | 1 | 0 | 0 | 0 | 0 | 1 | 0 |
| [DB14001](https://www.drugbank.ca/drugs/DB14001) | alpha-Tocopherol succinate | PPP2CA | 1 | 0 | 0 | 0 | 0 | 0 | 1 | 0 |
| [DB14002](https://www.drugbank.ca/drugs/DB14002) | D-alpha-Tocopherol acetate | PPP2CA | 1 | 0 | 0 | 0 | 0 | 0 | 1 | 0 |
| [DB14487](https://www.drugbank.ca/drugs/DB14487) | Zinc acetate | APOA2 | 1 | 0 | 0 | 0 | 0 | 0 | 0 | 1 |
| [DB14488](https://www.drugbank.ca/drugs/DB14488) | Ferrous gluconate | FEN1 | 1 | 1 | 0 | 0 | 0 | 0 | 1 | 0 |
| [DB14489](https://www.drugbank.ca/drugs/DB14489) | Ferrous succinate | FEN1 | 1 | 1 | 0 | 0 | 0 | 0 | 1 | 0 |
| [DB14490](https://www.drugbank.ca/drugs/DB14490) | Ferrous ascorbate | FEN1 | 1 | 1 | 0 | 0 | 0 | 0 | 1 | 0 |
| [DB14491](https://www.drugbank.ca/drugs/DB14491) | Ferrous fumarate | FEN1 | 1 | 1 | 0 | 0 | 0 | 0 | 1 | 0 |
| [DB14501](https://www.drugbank.ca/drugs/DB14501) | Ferrous glycine sulfate | FEN1 | 1 | 1 | 0 | 0 | 0 | 0 | 1 | 0 |
| [DB14517](https://www.drugbank.ca/drugs/DB14517) | Aluminium phosphate | KLK1 | 1 | 0 | 0 | 0 | 0 | 0 | 0 | 0 |
| [DB14518](https://www.drugbank.ca/drugs/DB14518) | Aluminum acetate | KLK1 | 1 | 0 | 0 | 0 | 0 | 0 | 0 | 0 |
| [DB14533](https://www.drugbank.ca/drugs/DB14533) | Zinc chloride | APOA2 | 1 | 0 | 0 | 0 | 0 | 0 | 0 | 1 |

Table S3.

Drugs currently in clinical trials ^39^ and predictions of the model. Antivirals that target viral proteins are not included in the model which is trained with gene expression datasets that only include human genes. Drugs with unknown targets of interventions that are not drugs cannot be modeled either.

| **Therapeutic intervention** | **Countries** | **No. of trials** | **No. of RCTs (% of trials per intervention)** | **Additional agents tested in the same interventional arm** | **Relevant in the model** | **Included (**✓**) / not in the model** |
| --- | --- | --- | --- | --- | --- | --- |
| TCM | China | 92 | 61 (66.3) | IFNα, LPV/r, ribavirin, chloroquine, umifenovir, ulinastatin | Ribavirin, chloroquine | TCM, umifenovir (do not have  a known or human target) |
| Antimalaria drugs (hydroxychloroquine, chloroquine, dihydroartemisinin piperaquine) | China, France, Germany, Mexico, Norway, Spain, Brazil, Canada | 35 | 30 (85.7) | Azithromycin, LPV/r, DRV/r or c, favipiravir, oseltamivir, umifenovir, IFNα | Hydroxychloroquine, chloroquine,  Oseltamivir | ✓ |
| Lopinavir ± ritonavir (LPV ± r) | China, Thailand, Hong Kong, UK, Europe, South Korea, Canada | 32 | 30 (93.8) | Chloroquine, IFNα, IFNβ, novaferon, thymosin a1, FTC/TAF, ribavirin, ebastine, oseltamivir, favipiravir, TCM | Ritonavir, Chloroquine, ribavirin, oseltamivir | Lopinavir (do not have a human target) |
| Cell therapy | China, USA, Brazil, Jordan | 30 | 19 (63.3) | Ruxolitinib | Ruxolitinib | Cell therapy (do not have a known target) |
| Interferon (IFNα, IFNα2β, IFNβ, rSIFN-co) | China, UK, Hong Kong, Europe | 22 | 21 (95.4) | Umifenovir, dihydroartemisinine piperaquine, TCM, bromhexine, favipiravir, LPV/r, ribavirin, ebastine, danoprevir/r, thalidomide, methylprednisolone, TTF2 | Ribavirin | Piperaquine,TCM  (do not have a known target) Favipiravir ,Umifenovir (do not have a human target) |
| Anti-IL-6 mAb (tocilizumab, sarilumab) | China, Italy, France, Switzerland, Denmark, USA, Canada, Global | 13 | 8 (61.5) | Favipiravir, adalimumab | Tocilizumab, sarilumab,  Adalimumab | Favipiravir (do not have a human Target) |
| Dietary/Supportive | China, Egypt | 14 | 12 (85.7) | N/A |  | Not a drug |
| Corticosteroids | China, UK, Italy | 11 | 9 (81.8) | IFNα umifenovir, thalidomide |  | Umifenovir (do not have a human Target) |
| Umifenovir | China | 11 | 11 (100) | novaferon, IFNα, IFNα2β, dihydroartemisinine piperaquine, bromhexine, favipiravir, thalidomide, methylprednisolone |  | Umifenovir (do not have a human  Target) |
| Convalescent plasma | China, Italy | 10 | 6 (60) | N/A |  | Not a drug |
| Adjuvant device | China | 10 | 3 (30) | N/A |  | Not a drug |
| Favipiravir | China, Thailand | 9 | 9 (100) | Tocilizumab, bromhexine, IFNα, umifenovir, (hydroxy)chloroquine, LPV/r, DRV/r | Tocilizumab, hydroxychloroquine,  Chloroquine | Favipiravir (do not have a human Target) |
| Remdesivir | Global, Europe, USA, China | 8 | 7 (87.5) | Hydroxychloroquine | Hydroxychloroquine | Remdesivir (do not have a human target) |
| Ventilation/Oxygenation strategies | China | 7 | 0 (0) | N/A |  | Not a drug |
| Macrolides (azithromycin, carrimycin) | China, Brazil, Denmark | 4 | 4 (100) | Hydroxychloroquine | Hydroxychloroquine | ✓ |
| Inhaled gases | China, USA, Italy | 4 | 4 (100) | N/A |  | Not a drug |
| JAK inhibitors (jakotinib, ruxolitinib, baricitinib) | China, Italy, Canada | 4 | 2 (50) | Cell therapy, ritonavir | Ruxolitinib, baricitinib | Cell therapy, ritonavir (do not have a human target) |
| ACE inhibitor/ARBs | China, USA | 3 | 2 (66.7) | N/A |  | N/A |
| Azvudine | China | 3 | 1 (33.3) | N/A |  | Azvudine (investigational drug,  not approved) |
| Danoprevir/ritonavir | China | 3 | 3 (100) | IFN |  | Danoprevir/ritonavir (do not have a human target) |
| Darunavir/cobicistat (DRV/r) | China, Thailand | 3 | 3 (100) | Thymosin a1, oseltamivir, hydroxychloroquine, favipiravir | Oseltamivir, hydroxychloroquine | Darunavir/ favipiravir (do not have a human target) |
| Pirfenidone | China | 3 | 3 (100) | N/A |  | ✓ |
| Anti-PD-1 mAb | China | 3 | 3 (100) | N/A |  | ✓ |
| Oseltamivir | Thailand, China | 3 | 3 (100) | Hydroxychloroquine, DRV/r, LPV/r | Oseltamivir, hydroxychloroquine | ✓ |
| Thymosin a1 | China | 3 | 3 (100) | DRV/c, LPV/r |  | Thymosin a1 (not in Drugbank DB) |
| Adalimumab | China | 2 | 2 (100) | Tocilizumab | Tocilizumab, adalimumab | ✓ |
| Bevazicumab | China, Italy | 2 | 1 (50) | N/A | Bevacizumab | ✓ |
| Colchicine | Italy, Canada | 2 | 2 (100) | N/A |  | ✓ |
| Novaferon | China | 2 | 2 (100) | LPV/r, umifenovir |  | LPV/r, umifenovir (do not have a human target) |
| Ribavirin | China, Hong Kong | 2 | 2 (100) | IFN, LPV/r |  | ✓ |
| Thalidomide | China | 2 | 2 (100) | IFNα, umifenovir, methylprednisolone |  | umifenovir (do not have a human target) |
| Ulinastatin | China | 2 | 1 (50) | TCM |  | Ulinastatin (investigational drug, not approved yet; target unknown) |
| ASC09/ritonavir | China | 2 | 2 (100) | N/A |  | ✓ |
| Intravenous Immunoglobulin | China | 2 | 2 (100) | N/A | Intravenous Immunoglobulin | ✓ |
| Acetylcysteine | China | 1 | 1 (100) | N/A | Acetylcysteine | ✓ |
| Antiviral peptide LL-37 | China | 1 | 0 (0) | N/A |  | Antiviral peptide LL-37 (do not have a human target) |
| Baloxavir marboxil | China | 1 | 1 (100) | N/A |  | Baloxavir marboxil (do not have a human target) |
| Bismuth potassium | China | 1 | 1 (100) | N/A |  | Bismuth potassium (do not have a human target) |
| Bronchoscopic alveolar lavage | China | 1 | 0 (0) | N/A |  | Not a drug |
| CD24Fc | USA | 1 | 1 (100) | N/A |  | Not a drug |
| Dipyridamole | China | 1 | 1 (100) | N/A | Dipyridamole | ✓ |
| Ebastine | China | 1 | 1 (100) | IFNα, LPV/r |  | Ebastine (do not have a known target) |
| Eculizumab | N/A | 1 | 0 (0) | N/A |  | ✓ |
| Enoxaparin | China | 1 | 1 (100) | N/A |  | ✓ |
| Escin | Italy | 1 | 0 (0) | N/A |  | Escin (not in Drugbank DB) |
| Fingolimod | China | 1 | 0 (0) | N/A | Fingolimod | ✓ |
| FTC/TAF | China | 1 | 1 (100) | LPV/r |  | FTC/TAF (do not have a human target) |
| hrIL-2 | China | 1 | 1 (100) | N/A |  | HrIL-2 (investigational drug, not approved yet; unknown target) |
| Inactivated mycobacterial vaccine | China | 1 | 1 (100) | N/A |  | Not a drug |
| Itraconazole | Belgium | 1 | 1 (100) | N/A |  | ✓ |
| Ixekizumab | China | 1 | 1 (100) | IFNα, ribavirin, chloroquine, LPV/r, umifenovir | Chloroquine | ✓ / umifenovir (do not have a human target) |
| Leflunomide | China | 1 | 1 (100) | N/A |  | ✓ |
| M1 | China | 1 | 1 (100) | N/A |  | M1 (not in Drugbank DB) |
| Polyinosinic/polycytidylic acid | China | 1 | 1 (100) | N/A |  | Polyinosinic/polycytidylic acid (not in DrugBank DB) |
| rhG-CSF | China | 1 | 1 (100) | N/A |  | ✓ |
| Sargramostin (GM-CSF) | Belgium | 1 | 1 (100) | N/A |  | ✓ |
| Sildenafil | China | 1 | 0 (0) | N/A |  | ✓ |
| Siltuximab | Italy | 1 | 0 (0) | N/A | Siltuximab | ✓ |
| Suramin | China | 1 | 0 (0) | N/A |  | Suramin (investigational drug, not approved yet; unknown target) |
| T89 | N/A | 1 | 1 (100) | N/A |  | T89 (not in Drugbank DB) |
| TFF-2 | China | 1 | 1 (100) | IFN-κ |  | transcription termination factor 2 (not in Drugbank DB) |
| TMPRSS2 inhibitor (camostat mesylate) | Denmark | 1 | 1 (100) | N/A |  | Camostat mesylate (investigational drug, not approved yet) |
| Tranilast | China | 1 | 1 (100) | N/A |  | Tranilast (investigational drug, not approved yet) |
| VIP | USA, Israel | 1 | 1 (100) | N/A |  | VIP (not in Drugbank DB) |
| vMIP | China | 1 | 0 (0) | N/A |  | vMIP (investigational drug, not approved yet) |
| Meplazumab (anti-CD147) | China | 1 | 1 (100) | N/A |  | ✓ |
| Sodium Aescinate | China | 1 | 1 (100) | N/A |  | Sodium Aescinate (do not have a known target) |
| Triazavirin | China | 1 | 1 (100) | N/A |  | Triazavirin (investigational drug, not approved yet) |

Table S4.

Selection of drugs targeting KDT with high relevance values.

| **DrugBank ID** | **Drug name** | ***Target full name*** | **Target** | **Target entrez ID** | **Associated conditions** | **Total.circuits.rel (277)** | **Host-virus interaction (61)** | **Inflammatory response (35)** | **Immune activity (32)** | **Antiviral defensa (48)** | **Endocytosis (16)** | **Replication (137)** | **Energetics (36)** |
| --- | --- | --- | --- | --- | --- | --- | --- | --- | --- | --- | --- | --- | --- |
| [DB00005](https://www.drugbank.ca/drugs/DB00005) | Etanercept | Tumor necrosis factor receptor superfamily member 1B | TNFRSF1B | 7133 | Ankylosing Spondylitis (AS); Graft Versus Host Disease (GVHD); Hidradenitis Suppurativa (HS); Polyarticular Juvenile Idiopathic Arthritis; Psoriasis Vulgaris (Plaque Psoriasis); Psoriatic Arthritis; Pyoderma Gangrenosum; Rheumatoid Arthritis | 271 | 59 | 35 | 32 | 47 | 16 | 133 | 34 |
| [DB00005](https://www.drugbank.ca/drugs/DB00005) | Etanercept | Tumor necrosis factor | TNF | 7124 | Ankylosing Spondylitis (AS); Graft Versus Host Disease (GVHD); Hidradenitis Suppurativa (HS); Polyarticular Juvenile Idiopathic Arthritis; Psoriasis Vulgaris (Plaque Psoriasis); Psoriatic Arthritis; Pyoderma Gangrenosum; Rheumatoid Arthritis | 267 | 59 | 35 | 30 | 46 | 14 | 135 | 33 |
| [DB00005](https://www.drugbank.ca/drugs/DB00005) | Etanercept | Lymphotoxin-alpha | LTA | 4049 | Ankylosing Spondylitis (AS); Graft Versus Host Disease (GVHD); Hidradenitis Suppurativa (HS); Polyarticular Juvenile Idiopathic Arthritis; Psoriasis Vulgaris (Plaque Psoriasis); Psoriatic Arthritis; Pyoderma Gangrenosum; Rheumatoid Arthritis | 246 | 57 | 31 | 27 | 41 | 13 | 127 | 32 |
| [DB00877](https://www.drugbank.ca/drugs/DB00877) | Sirolimus | Fibroblast growth factor 2 | FGF2 | 2247 | Chordomas; Graft Versus Host Disease (GVHD); Heart Transplant Rejection; Liver Transplant Rejection; Lung Transplant Rejection; Lymphangioleiomyomatosis; Renal Angiomyolipomas | 189 | 45 | 22 | 24 | 30 | 10 | 102 | 25 |
| [DB12159](https://www.drugbank.ca/drugs/DB12159) | Dupilumab | Interleukin-4 receptor subunit alpha | IL4R | 3566 | Moderate to Severe Asthma; Severe Atopic Dermatitis | 178 | 35 | 22 | 15 | 27 | 11 | 84 | 27 |
| [DB00471](https://www.drugbank.ca/drugs/DB00471) | Montelukast | Cysteinyl leukotriene receptor 1 | CYSLTR1 | 10800 | Asthma; Exercise-Induced Bronchoconstriction (EIB); Perennial Allergic Rhinitis (PAR) | 136 | 27 | 15 | 14 | 24 | 5 | 71 | 23 |
| [DB01234](https://www.drugbank.ca/drugs/DB01234) | Dexamethasone | Nuclear receptor subfamily 1 group I member 2 | NR1I2 | 8856 | Acne Rosacea; Acute Gouty Arthritis; Adrenal cortical hypofunctions; Adrenocortical Hyperfunction; Alopecia Areata (AA); Ankylosing Spondylitis (AS); Anterior Segment Inflammation; Aspiration Pneumonitis; Asthma; Atopic Dermatitis (AD); Berylliosis; Bullous dermatitis herpetiformis; Bursitis; Choroiditis; Congenital Adrenal Hyperplasia (CAH); Congenital Hypoplastic Anemia; Conjunctivitis, Seasonal Allergic; Corneal Inflammation; Cushing’s Syndrome; Dermatitis exfoliative generalised; Dermatitis, Contact; Diabetic Macular Edema (DME); Discoid Lupus Erythematosus (DLE); Drug hypersensitivity reaction; Edema of the cerebrum; Epicondylitis; Erythroblastopenia; Hypercalcemia; Idiopathic Thrombocytopenic Purpura; Inflammatory Reaction; Iridocyclitis; Iritis; Keloid Scars; Leukemia, Acute; Lichen Planus (LP); Lichen simplex chronicus; Loeffler&#39;s syndrome; Macular Edema (ME); Malignant Lymphomas; Mycosis Fungoides (MF); Necrobiosis lipoidica diabeticorum; Ophthalmia, Sympathetic; Optic Neuritis; Pemphigus; Perennial Allergic Rhinitis (PAR); Post-traumatic Osteoarthritis; Regional Enteritis; Rheumatoid Arthritis; Rheumatoid Arthritis, Juvenile; Sarcoidosis; Seasonal Allergic Rhinitis (SAR); Secondary thrombocytopenia; Serum Sickness; Severe Seborrheic Dermatitis; Stevens-Johnson Syndrome; Synovitis; Systemic Lupus Erythematosus (SLE); Trichinosis; Tuberculosis Infection; Tuberculosis Meningitis; Ulcerative Colitis; Uveitis; Acquired immune hemolytic anemia; Acute nonspecific tenosynovitis; Acute rheumatic carditis; Diffuse posterior uveitis; Granuloma annulare lesions; Non-suppurative Thyroiditis; Severe Psoriasis | 85 | 12 | 16 | 12 | 16 | 8 | 33 | 9 |
| [DB00198](https://www.drugbank.ca/drugs/DB00198) | Oseltamivir | Liver carboxylesterase 1 | CES1 | 1066 | Influenza A Virus Infection; Influenza A, Influenza B | 51 | 8 | 5 | 7 | 7 | 3 | 19 | 11 |
| [DB00005](https://www.drugbank.ca/drugs/DB00005) | Etanercept | Complement C1q subcomponent subunit A | C1QA | 712 | Ankylosing Spondylitis (AS); Graft Versus Host Disease (GVHD); Hidradenitis Suppurativa (HS); Polyarticular Juvenile Idiopathic Arthritis; Psoriasis Vulgaris (Plaque Psoriasis); Psoriatic Arthritis; Pyoderma Gangrenosum; Rheumatoid Arthritis | 10 | 2 | 1 | 1 | 2 | 2 | 3 | 2 |
| [DB00074](https://www.drugbank.ca/drugs/DB00074) | Basiliximab | Complement C1q subcomponent subunit A | C1QA | 712 |  | 10 | 2 | 1 | 1 | 2 | 2 | 3 | 2 |
| [DB01611](https://www.drugbank.ca/drugs/DB01611) | Hydroxychloroquine | Toll-like receptor 7 | TLR7 | 51284 | Discoid Lupus Erythematosus (DLE); Plasmodium Infections; Porphyria Cutanea Tarda; Q Fever; Rheumatoid Arthritis; Sjögren’s Syndrome; Systemic Lupus Erythematosus (SLE); Uncomplicated Malaria caused by Plasmodium Vivax; Uncomplicated Malaria caused by Plasmodium malariae; Uncomplicated Malaria caused by Plasmodium ovale | 9 | 0 | 2 | 3 | 2 | 1 | 2 | 3 |
| [DB00091](https://www.drugbank.ca/drugs/DB00091) | Ciclosporin | Peptidyl-prolyl cis-trans isomerase F, mitochondrial | PPIF | 10105 | Atopic Dermatitis (AD); Bone Marrow Transplant Rejection; Connective Tissue Disorders; Glomerulonephritis minimal lesion; Glomerulosclerosis, Focal Segmental; Graft Versus Host Disease (GVHD); Heart Transplant Rejection; Idiopathic Thrombocytopenic Purpura; Interstitial Cystitis; Juvenile Idiopathic Arthritis (JIA); Kidney Transplant Rejection; Liver Transplant Rejection; Nephritis, Lupus; Ocular Rosacea; Psoriasis; Severe Ulcerative Colitis; Uveitis; Blistering disorder; Refractory Ulcerative colitis; Severe, active Rheumatoid arthritis; Severe, recalcitrant Plaque psoriasis | 7 | 1 | 2 | 1 | 3 | 0 | 1 | 1 |
| [DB00074](https://www.drugbank.ca/drugs/DB00074) | Basiliximab | Interleukin-2 receptor subunit alpha | IL2RA | 3559 |  | 3 | 0 | 0 | 0 | 2 | 0 | 0 | 1 |
| [DB00877](https://www.drugbank.ca/drugs/DB00877) | Sirolimus | Serine/threonine-protein kinase mTOR | MTOR | 2475 | Chordomas; Graft Versus Host Disease (GVHD); Heart Transplant Rejection; Liver Transplant Rejection; Lung Transplant Rejection; Lymphangioleiomyomatosis; Renal Angiomyolipomas | 1 | 1 | 0 | 0 | 0 | 0 | 1 | 0 |
